# Design, Synthesis, and Antiproliferative Evaluation of C3/C12-Modified Panaxadiol Derivatives Against Gastric Cancer Cells: Integrated Transcriptomic and Metabolomic Analyses

**DOI:** 10.3390/molecules31183225

**Published:** 2026-09-12

**Authors:** Yueru Zhang, Hongqing Xie, Chenggang Shan, Jinlong Han, Fangzhou Zhao, Xianchang Wang, Yinan Qi, Xiaoyang Li, Jianhua Zhang, Feng Zhang, Yun Zhou

**Affiliations:** 1 Institute of Industrial Crops, Shandong Academy of Agricultural Sciences, Jinan 250100, China; 13553169667@163.com (Y.Z.); xiehongqing@zju.edu.cn (H.X.); shanchenggang@126.com (C.S.); goldendragonh@163.com (J.H.); 19508465248@163.com (F.Z.); archangw@163.com (X.W.); 19862613161@163.com (Y.Q.); zhangjianhua198904@163.com (J.Z.); zhangfeng0723@126.com (F.Z.); 2 School of Pharmacy, Shandong University of Traditional Chinese Medicine, Jinan 250355, China; 3 Department of Applied Chemistry, China Agricultural University, Beijing 100193, China; b20233100844@cau.edu.cn

**Keywords:** panaxadiol, C3/C12 site-differentiated diversification, gastric cancer, integrated multi-omics, structure–activity relationship

## Abstract

Panaxadiol (PD) is a bioactive dammarane triterpenoid with limited antiproliferative potency, and systematic optimization of its C3 and C12 positions remains underexplored. Here, a stepwise, site-differentiated strategy was established by combining selective C3 esterification with late-stage C12 diversification through a chloroacetyl–piperazine linker, affording 23 PD derivatives and enabling complementary C3/C12 structure–activity analysis. Antiproliferative screening in AGS gastric cancer cells identified compounds **6**, **11**, and **17** as the most active analogues, with IC_50_ values of 8.41, 5.08, and 6.78 μM, respectively, all outperforming 5-fluorouracil under identical conditions. Compound **6** provided a relatively favorable balance between low-micromolar activity and preservation of non-malignant GES-1 cells within a defined concentration range and was therefore selected for mechanistic investigation. Transcriptomic profiling identified 298 differentially expressed genes associated with cellular stress, cytokine signaling, epithelial growth regulation, and extracellular remodeling, whereas metabolomic analysis revealed marked perturbation of glycerophospholipid, choline, polyunsaturated fatty acid, and glutathione metabolism. Integrated analysis highlighted membrane-lipid remodeling, redox dysregulation, and ferroptosis-related processes as central features of the response. These findings establish a modular platform for PD optimization and identify compound **6** as a promising lead for further anti-gastric cancer development.

## 1. Introduction

Gastric cancer remains a major global health burden. According to the latest GLOBOCAN estimates, approximately 980,000 new cases and 642,000 deaths were attributable to gastric cancer worldwide in 2024, ranking it fifth in both cancer incidence and cancer-related mortality [1]. Although advances in surgery, cytotoxic chemotherapy, molecularly targeted therapy, and immune checkpoint blockade have improved the management of selected patient populations, the prognosis of advanced gastric cancer remains unsatisfactory. The effectiveness of chemotherapy is frequently compromised by intrinsic or acquired resistance involving reduced drug uptake, enhanced drug efflux, altered drug activation or inactivation, changes in therapeutic targets, increased DNA-damage repair, and evasion of apoptosis [2]. Treatment-related toxicity further limits dose intensity and long-term clinical benefit. These challenges underscore the continuing need for structurally novel anticancer agents with improved potency, cellular selectivity, and drug-like properties.

Natural products provide structurally diverse and biologically relevant scaffolds for anticancer drug discovery. *Panax ginseng* and its ginsenosides have attracted particular attention because of their broad pharmacological activities and potential value in cancer-related applications. Experimental and clinical evidence indicates that ginseng-derived preparations and individual ginsenosides may alleviate chemotherapy-associated nephrotoxicity, hepatotoxicity, cardiotoxicity, immunotoxicity, and hematopoietic injury through antioxidant, anti-inflammatory, antiapoptotic, and tissue-protective mechanisms [3]. Representative ginsenosides also display direct antitumor activity; for example, ginsenoside Rg_5_ induced apoptosis and DNA damage in human cervical cancer cells [4], while ginsenoside Rh_2_ has been reported to inhibit tumor-cell proliferation, invasion, migration, and metastasis, induce cell-cycle arrest and apoptosis, and modulate multidrug resistance [5]. In addition, *Panax ginseng* extracts and selected ginsenoside fractions exhibited radioprotective effects in irradiated mice [6]. These observations collectively establish ginseng-derived dammarane scaffolds as a pharmacologically productive source of lead structures. Nevertheless, natural origin does not necessarily confer sufficient potency, selectivity, or favorable pharmaceutical properties, and rational structural optimization is commonly required for further drug development.

Panaxadiol (PD) is a dammarane-type pseudoaglycone generated from protopanaxadiol-type ginsenosides through acid hydrolysis or metabolic transformation. Importantly, PD is structurally distinct from protopanaxadiol and should not be regarded as an interchangeable form of the latter. Previous studies have demonstrated that PD possesses direct antiproliferative activity and can enhance the effects of both conventional chemotherapeutic agents and naturally derived polyphenols. PD potentiated 5-fluorouracil-induced cell-cycle arrest and apoptosis in HCT-116 colorectal cancer cells [7]. Its combination with epicatechin markedly enhanced growth inhibition and apoptosis in HCT-116 cells [8], while co-treatment with epigallocatechin gallate produced enhanced antiproliferative and proapoptotic effects in both HCT-116 and SW-480 cells [9]. These findings establish PD as a pharmacologically active anticancer scaffold. However, they were predominantly obtained in colorectal cancer models, and the moderate activity of PD as a single agent indicates substantial scope for structural optimization and evaluation in other tumor types, including gastric cancer.

Structural modification is an established strategy for expanding the chemical space and improving the biological and physicochemical properties of dammarane-type compounds [10]. Previous approaches have included esterification, amino acid conjugation, sulfation, PEGylation, oxidation, glycosylation, and incorporation of nitrogen-containing heterocycles. However, the outcomes of these studies must be interpreted according to the specific parent scaffold. Sulfation of 20(*S*)-ginsenoside Rh_2_ increased its aqueous solubility and enhanced its anti-inflammatory activity [11], whereas PEGylation substantially improved the gastric stability of ginsenoside Rg_1_ [12]. Amino acid esterification of 25-hydroxyprotopanaxadiol generated analogues with enhanced antiproliferative activity, with the most active derivative showing an approximately tenfold improvement over the parent compound [13]. Although Rh_2_, Rg_1_, and 25-hydroxyprotopanaxadiol are structurally related to PD, they are distinct compounds; therefore, these studies provide a general medicinal chemistry rationale for introducing polar or ionizable groups into dammarane scaffolds, rather than direct evidence that the same modifications necessarily improve the solubility or activity of PD.

The C3 and C12 hydroxyl groups of PD provide chemically accessible positions for semisynthetic diversification. Previous PD studies have predominantly focused on C3 functionalization. Introduction of 1,2,4-triazole-containing groups at C3 yielded derivatives with substantially enhanced antiproliferative activity. The most active compound inhibited HepG-2 cell proliferation with an IC_50_ value of 4.21 μM, approximately 15-fold more potently than PD, and induced mitochondria-associated apoptosis [14]. That study also suggested that, within the examined series, C3-substituted compounds were generally more active than the corresponding C12-substituted analogues. This finding supports C3 as a productive modification site, but it does not exclude the potential value of C12 functionalization when combined with a preoptimized C3 substituent.

Piperazine is a widely used medicinal chemistry motif because its two nitrogen atoms provide tunable basicity, potential salt-forming capacity, and convenient sites for further structural diversification. Piperazine-containing derivatives of PD and related dammarane scaffolds have demonstrated low-micromolar antiproliferative activity. In one representative study, the most active derivative inhibited PC-3 cell proliferation with an IC_50_ value of 1.98 μM and induced reactive oxygen species accumulation, G1-phase arrest, Bax translocation, cytochrome *c* release, and caspase-dependent apoptosis [15]. More broadly, piperazine-containing compounds have been reported to inhibit tumor-cell proliferation and migration through oxidative stress-associated mitochondrial apoptosis [16]. A piperazine alkyl compound also inhibited NUGC-3 gastric carcinoma cells and induced apoptosis through ROS-mediated c-Abl/p38 MAPK/RhoB signaling [17]. These studies support the pharmacological utility of piperazine as a functional motif in anticancer compound design. Nevertheless, because the latter compounds are not PD derivatives, they do not directly demonstrate that piperazine incorporation improves the aqueous solubility or gastric cancer-cell selectivity of PD.

Despite these advances, several important questions remain unresolved. Most reported PD derivatives have been evaluated in colorectal, hepatic, prostate, breast, lung, or other non-gastric cancer models, whereas systematic studies in gastric cancer cells remain limited. Previous investigations have also largely emphasized single-site C3 modification or parallel derivatization at individual hydroxyl groups. The relative superiority of C3 modification observed in some compound series has led to a greater focus on this position; however, the medicinal chemistry potential of C12 functionalization after prior optimization of the C3 substituent remains insufficiently explored. It is therefore unclear whether an activity-guided strategy combining initial C3 optimization with subsequent C12 diversification can broaden the accessible chemical space and improve activity against gastric cancer cells. Furthermore, the transcriptional and metabolic responses induced by structurally optimized PD derivatives in gastric cancer cells have not been comprehensively characterized.

**Figure 1:**
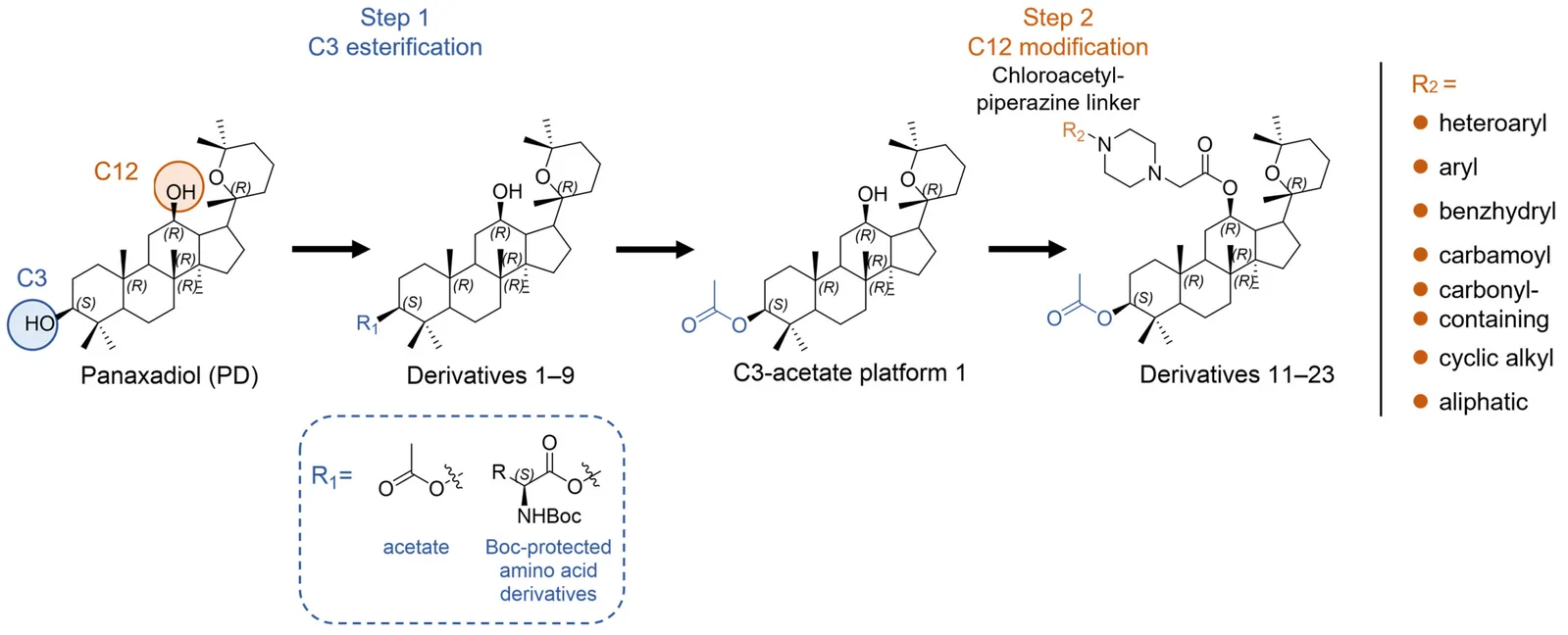
Conceptual design of the activity-guided, stepwise, site-differentiated diversification of panaxadiol (PD).

In the present study, an activity-guided, stepwise C3/C12 modification strategy was developed to optimize the biological properties of PD. Acetyl and amino acid-derived acyl groups were introduced at the C3 hydroxyl group to generate a structurally diverse series of C3-modified derivatives. Following comparative activity evaluation, a sterically accessible C3-modified scaffold was selected for C12 chloroacetylation and subsequent diversification with piperazine-containing substituents. A total of 23 PD derivatives were synthesized and evaluated for their antiproliferative effects in human AGS gastric cancer cells, with nonmalignant human gastric epithelial GES-1 cells used to assess preliminary cellular selectivity. Compounds **6**, **11**, and **17** exhibited lower IC_50_ values against AGS cells than 5-fluorouracil (5-Fu), while compound **6** showed a comparatively favorable balance between AGS-cell inhibition and GES-1-cell response. Integrated transcriptomic and metabolomic analyses further associated the cellular effects of compound **6** with stress- and cytokine-related transcriptional responses and extensive perturbation of lipid metabolic networks, particularly glycerophospholipid and cancer-associated choline metabolism. Collectively, these findings demonstrate the value of sequential structural diversification of the PD scaffold, identify both C3- and C3/C12-modified derivatives with enhanced activity against gastric cancer cells, and provide a lead compound and molecular framework for further mechanistic and preclinical investigation. The overall site-differentiated modification strategy and library-generation logic are summarized in Figure 1.

## 2. Results

### 2.1. Design and Synthesis of Panaxadiol Derivatives

#### 2.1.1. Synthesis of C3-Modified Panaxadiol Derivatives

**Scheme 1:**
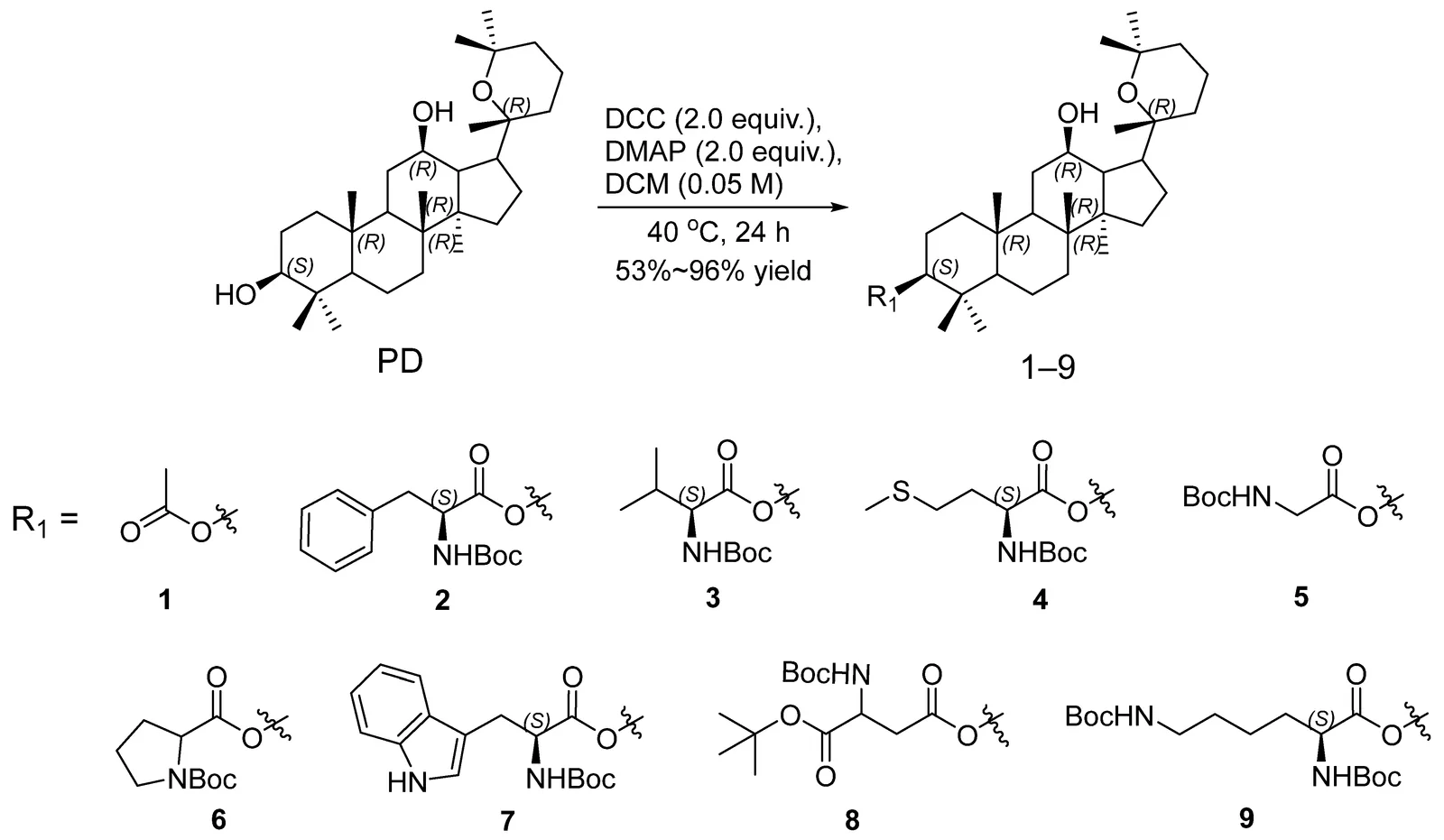
Synthetic Route for PD C-3 Site Modification.

A two-stage, site-differentiated derivatization strategy was employed to expand the chemical space of panaxadiol (PD). As shown in Scheme 1, the first-stage library was generated by selective esterification of the C3 hydroxyl group with acetic acid or Boc-protected amino acid-derived carboxylic acids, while the C12 hydroxyl group was retained for subsequent functionalization. Under N, N’-Dicyclohexylcarbodiimide (DCC)/4-(dimethylamino)pyridine (DMAP)-mediated esterification conditions in dichloromethane at 40 °C for 24 h, compounds **1**–**9** were obtained, with most isolated yields reaching approximately 70%.

The resulting series incorporated acetyl and glycine-, valine-, methionine-, proline-, phenylalanine-, tryptophan-, aspartate-, and lysine-derived substituents, thereby systematically varying steric bulk, conformational rigidity, aromaticity, side-chain length, and hydrogen-bonding characteristics within a common PD scaffold. Because the amino groups remained Boc-protected, this series primarily evaluated the influence of substituent topology and overall lipophilic–polar balance rather than that of freely ionizable amino groups. Retention of the C12 hydroxyl group also preserved an orthogonal reactive site for the subsequent construction of C3/C12 dual-modified derivatives. The structures of all C3-modified products were confirmed by ^1^H NMR, ^13^C NMR, and HRMS, and the complete characterization data are provided in the Supplementary Materials.

#### 2.1.2. Synthesis of C3/C12 Dual-Modified Derivatives

**Scheme 2:**
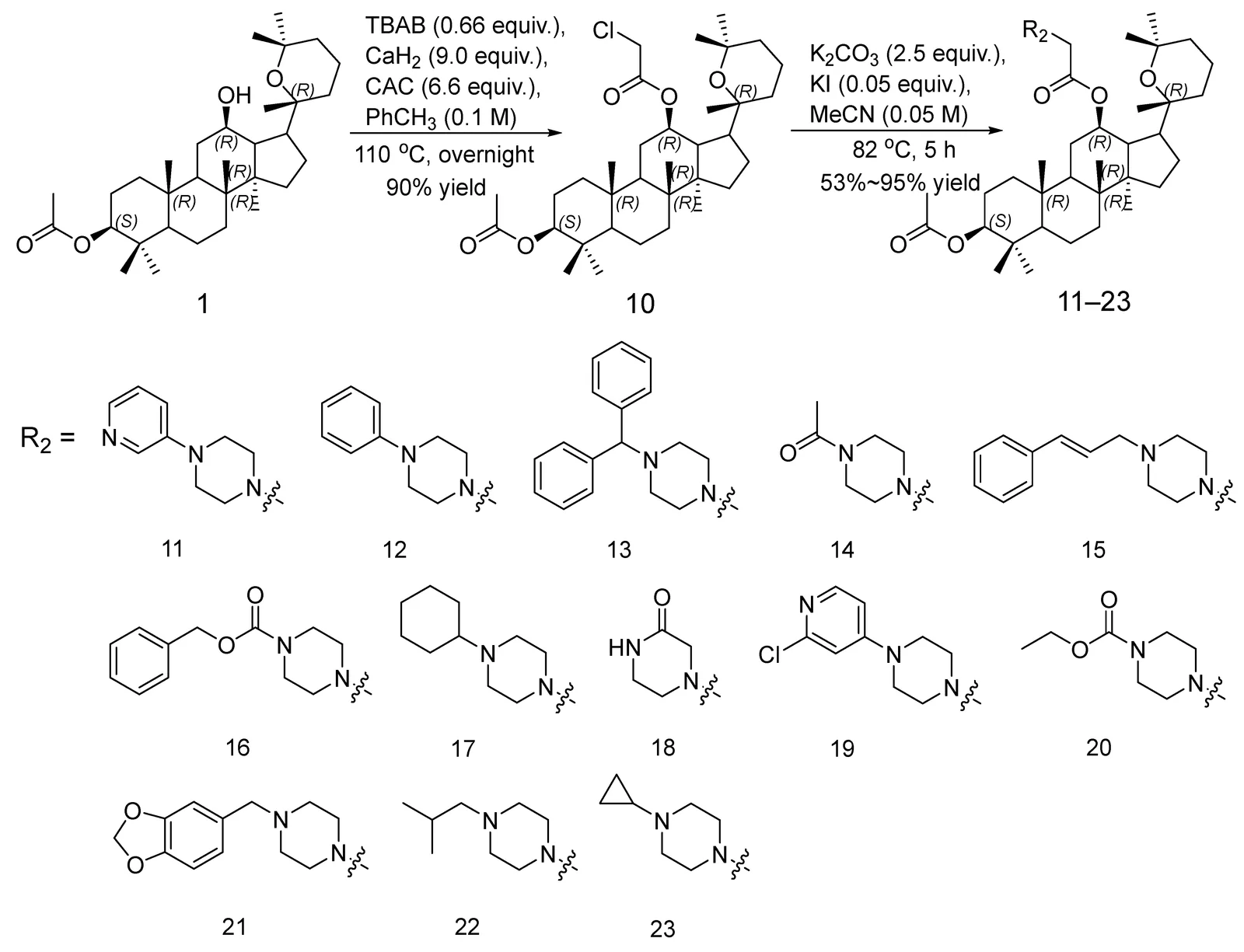
Synthetic Route for C-12 Modification of Compound **1**.

In the second stage, compound **1** was selected as the common C3-acetylated scaffold for C12 diversification because of its convenient synthetic accessibility, low steric demand, and improved antiproliferative activity relative to the parent PD. In contrast to the more accessible C3 hydroxyl group, the C12 hydroxyl group is embedded within a sterically congested region of the rigid dammarane skeleton and forms a stable intramolecular hydrogen bond with the ether oxygen atom of the tetrahydropyran ring in the C17 aliphatic side chain. This hydrogen bonding further reduces the nucleophilicity of the C12 hydroxyl group, resulting in relatively low chemical reactivity and limited reaction controllability [18]. To address this synthetic challenge, a stepwise activation–diversification strategy was developed (Scheme 2). Compound **1** was first reacted with chloroacetyl chloride in the presence of calcium hydride (CaH_2_) and tetrabutylammonium bromide (TBAB) in toluene at 110 °C, affording the electrophilic chloroacetate intermediate 10. This transformation converted the poorly reactive C12 hydroxyl group into a synthetically accessible functional handle while preserving the C3 acetate and the stereochemically defined PD framework.

Intermediate 10 was subsequently subjected to nucleophilic substitution with a structurally diverse panel of piperazine derivatives using potassium carbonate (K_2_CO_3_) and potassium iodide (KI) in acetonitrile at 82 °C, furnishing compounds **11**–**23** in moderate-to-excellent isolated yields. The resulting 13-compound congeneric series retained a common C3-acetylated PD scaffold and C12 oxyacetyl–piperazine linker while incorporating diverse heteroaryl, aryl, benzhydryl, carbamoyl, carbonyl-containing, cyclic alkyl, and flexible aliphatic terminal substituents. Because the C3 acetate and C12 oxyacetyl–piperazine linker were maintained across the entire series, differences in biological activity could be more directly related to the steric, electronic, conformational, and lipophilic properties of the distal piperazine substituents. This modular late-stage diversification strategy therefore overcame the intrinsic difficulty of C12 functionalization and enabled efficient construction of a focused analogue library for delineating C12-directed structure–activity relationships. All products were fully characterized by ^1^H NMR, ^13^C NMR, and HRMS, and the complete analytical data are provided in the Supplementary Materials.

### 2.2. Antiproliferative Screening, Structure–Activity Relationships, and Lead Selection

#### 2.2.1. Primary Screening and Hit Identification

The antiproliferative activities of PD and derivatives **1**–**23** were evaluated in AGS human gastric cancer cells using the Cell Counting Kit-8 (CCK-8, Puxitang Biotech Co., Ltd., Beijing, China) assay, with 5-fluorouracil (5-Fu) as the positive control (Table 1). PD showed moderate activity, with an IC_50_ value of 38.29 ± 6.31 μM. Thirteen of the 23 derivatives (56.5%) were more active than PD, including six of nine C3-only derivatives (66.7%) and seven of thirteen terminal C3/C12 piperazine derivatives (53.8%). Nine derivatives achieved IC_50_ values below 25 μM, and three entered the sub-10-μM range. Compounds **6**, **11**, and **17** were more potent than 5-Fu under the same assay conditions (IC_50_ = 14.42 ± 1.52 μM). Compound **6**, the N-Boc-L-proline ester, inhibited AGS cells with an IC_50_ of 8.41 ± 0.61 μM; compound **11**, bearing a pyridylpiperazine substituent, had an IC_50_ of 5.08 ± 0.13 μM; and compound **17**, bearing a cyclohexylpiperazine substituent, had an IC_50_ of 6.78 ± 1.07 μM. Relative to PD, these values corresponded to approximately 4.6-, 7.5-, and 5.6-fold increases in potency, respectively.

#### 2.2.2. Structure–Activity Relationships Across the C3 and C12 Series

The activity distribution revealed marked, non-linear structure–activity relationships (Table 1). In the C3 series, the compact acetate derivative **1** (19.54 ± 1.66 μM) and glycine derivative **5** (17.14 ± 2.76 μM) provided approximately twofold improvements over PD, whereas conformationally constrained compound **6** was approximately 2.3-fold more potent than compound **1** and was the strongest C3-only analogue. Valine-, methionine-, and aspartate-derived compounds **3**, **4**, and **8** retained moderate activity, but the bulky aromatic phenylalanine and tryptophan derivatives **2** and **7** were less active than PD. The extended lysine derivative **9** did not reach 50% inhibition at the highest tested concentration of 64 μM. These results show that attachment of an amino acid-derived residue was not intrinsically advantageous; in this protected series, a compact or conformationally organized substituent was more favorable than an extended or highly aromatic side chain. In the C12 branch, intermediate 10 showed no 50% inhibitory effect within the tested concentration range up to 64 μM, whereas terminal substitution with pyridylpiperazine (11) or cyclohexylpiperazine (17) increased potency by at least 19.7-fold and 14.7-fold, respectively. Phenyl and benzhydryl derivatives **12** and **13** similarly failed to achieve 50% growth inhibition at 64 μM; accordingly, their IC_50_ values were inferred to be greater than 100 μM based on nonlinear regression fitting of the dose–response curves. In contrast, the carbonyl-containing analogues 14, 18, and 20 showed intermediate activity (IC_50_ = 15.27–17.26 μM). Chlorination of the pyridyl ring decreased potency from 5.08 μM for compound **11** to 26.06 μM for compound **19**, a 5.1-fold loss. The retained activity of the small cyclopropyl analogue 23 (24.24 ± 2.64 μM), together with the weak activity of several bulky hydrophobic analogues, further indicated that neither molecular size nor lipophilicity alone explained the AGS-cell response. The most informative result of the C12 series was therefore not a general advantage of dual-site modification, but the discovery of discrete activity cliffs controlled by the identity and substitution pattern of the distal piperazine group.

#### 2.2.3. Preliminary Cellular Selectivity and Mechanistic-Lead Nomination

To obtain an initial assessment of cellular safety and selectivity, the concentration–response profiles of the three prioritized derivatives (compounds **6**, **11**, and **17**) were evaluated in AGS gastric cancer cells and non-malignant GES-1 gastric epithelial cells, with the clinical first-line anticancer drug 5-fluorouracil (5-Fu) included as a positive control for benchmark comparison. The IC_50_ values of compounds **6**, **11**, and **17** against AGS cells were 8.41 ± 0.61 μM, 5.08 ± 0.13 μM, and 6.78 ± 1.07 μM, respectively (detailed data with 95% confidence intervals (CIs) are provided in Table 1). The corresponding IC_50_ values against non-malignant GES-1 cells were determined to be 8.35 ± 0.10 μM (95% CI: 8.25–8.45 μM), 2.63 ± 0.09 μM (95% CI: 2.54–2.72 μM), and 4.78 ± 0.13 μM (95% CI: 4.65–4.91 μM), respectively. For the positive control 5-Fu, the IC_50_ value against AGS cells was 14.42 ± 1.52 μM (95% CI: 12.90–15.94 μM), and the IC_50_ value against GES-1 cells was 16.65 ± 1.04 μM (95% CI: 15.61–17.68 μM). The selectivity index (SI), defined as the ratio of the IC_50_ value in GES-1 normal epithelial cells to that in AGS tumor cells, was calculated to be 0.99, 0.52, and 0.70 for compounds **6**, **11**, and **17**, respectively.

**Figure 2:**
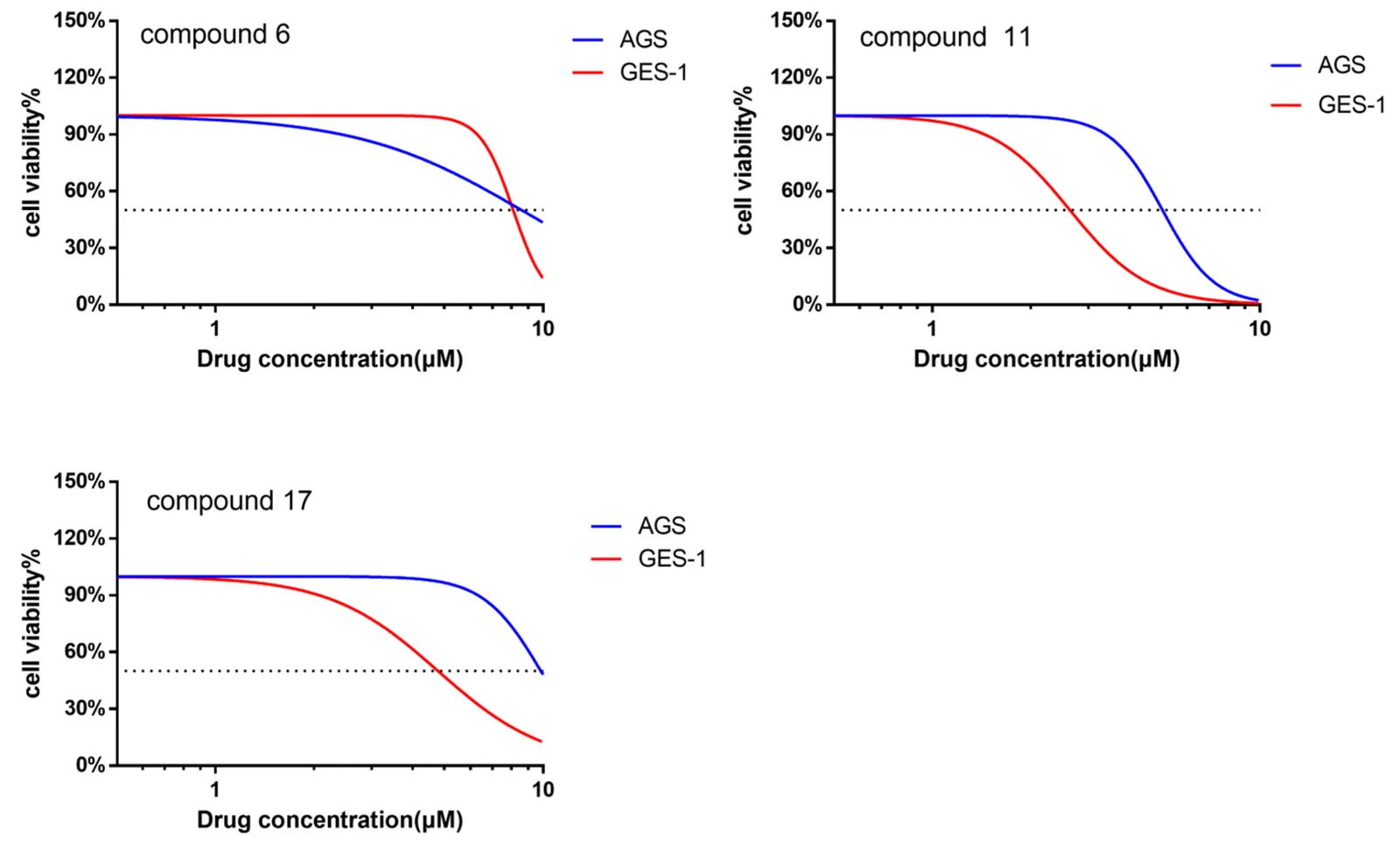
Concentration-response profiles of compounds **6**, **11**, and **17** in AGS gastric cancer cells and GES-1 nonmalignant gastric epithelial cells. The dashed line represents 50% cell viability. Data are presented as mean ± SD from three independent biological experiments, each with three technical replicates.

As shown in Figure 2, none of the three compounds exhibited unambiguous tumor-selective antiproliferative activity, as all SI values were below 1. Compound **6**, however, displayed the most favorable differential response profile between the two cell lines. At concentrations up to approximately 8 μM, compound **6** markedly inhibited AGS cell proliferation while only marginally affecting GES-1 cell viability, forming a narrow concentration window with partial preferential activity against gastric cancer cells. At higher concentrations, however, GES-1 viability declined substantially and the two response curves gradually converged, indicating that this partial advantage did not extend across the full concentration range. In contrast, compounds **11** and **17** induced pronounced reductions in GES-1 viability at concentrations comparable to, or even lower than, those required to inhibit AGS cell proliferation, indicating less favorable preliminary safety profiles.

Taken together, among the three candidate compounds, compound **6** achieved the best balance between low-micromolar antiproliferative potency against tumor cells and relative preservation of non-malignant gastric epithelial cells, making it the most promising lead candidate for further development. Although its current selectivity index is 0.99 and it does not yet show significant tumor selectivity, this well-balanced biological profile provides a favorable starting point for subsequent structural optimization and targeted modification. The tumor targeting ability and therapeutic window of compound **6** can be further improved in follow-up work through strategies such as side chain structural modification and construction of targeted delivery systems. Based on these considerations, we selected compound **6** for subsequent transcriptomic and metabolomic mechanistic studies.

It should be explicitly noted that the current selectivity assessment is limited to a single non-malignant gastric epithelial cell line (GES-1), and the results represent only a preliminary cellular safety profile. Evaluation in additional normal cell models and in vivo toxicity studies will be required to comprehensively characterize the safety and selectivity of these derivatives.

### 2.3. Effects of Compound ***6*** on Gene Transcription Levels in Gastric Cancer Cells

**Figure 3:**
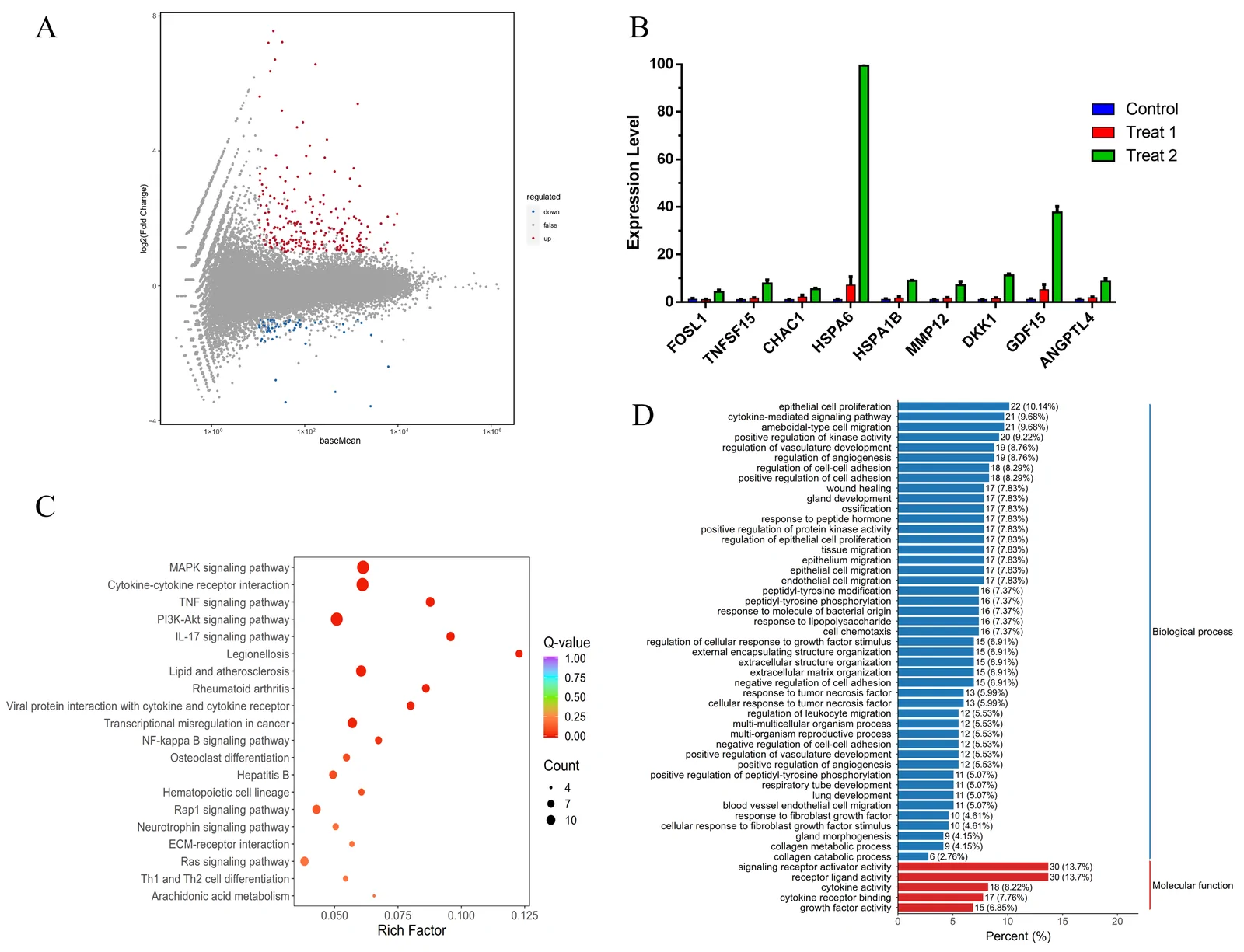
Transcriptomic profiling analysis of differentially expressed genes (DEGs) in compound **6**-treated AGS gastric cancer cells. (**A**) MA plot of DEGs. Red dots represent upregulated genes, blue dots indicate downregulated genes, and gray dots represent genes with no significant differential expression. (**B**) qPCR validation bar plot of nine representative DEGs under two treatment concentrations (Treat 1: 5 μM; Treat 2: 8 μM). (**C**) Bubble plot of KEGG pathway enrichment for DEGs. A larger Rich factor indicates a higher enrichment degree. Larger dots represent pathways enriched with more differentially expressed genes, while redder dots indicate more significant enrichment. (**D**) GO enrichment bar plot of DEGs. The numbers in the figure represent the counts of differentially expressed genes annotated to each GO term. The values in parentheses indicate the ratio of annotated DEGs in each term to the total annotated DEGs, and the labels on the far right represent the classification of each GO term.

To characterize the transcriptional alterations associated with the antiproliferative effect of compound **6**, RNA sequencing was performed in AGS gastric cancer cells following compound treatment. Differential expression analysis identified 298 differentially expressed genes (DEGs), including 236 upregulated genes (79.2%) and 62 downregulated genes (20.8%), corresponding to an upregulated-to-downregulated ratio of approximately 3.8:1 (Figure 3A). The predominance of upregulated transcripts indicated that compound **6** elicited an active transcriptional response rather than nonspecific global transcriptional suppression. The MA plot further showed that significant changes occurred across a broad range of transcript abundance, demonstrating that the response was not restricted to a small subset of highly expressed genes.

Nine representative DEGs—*FOSL1*, *TNFSF15*, *CHAC1*, *HSPA6*, *HSPA1B*, *MMP12*, *DKK1*, *GDF15*, and *ANGPTL4*—were selected for qPCR validation based on their associations with cellular stress, cytokine signaling, epithelial growth regulation, extracellular remodeling, and metabolic adaptation. All nine genes exhibited expression trends consistent with those observed in the RNA-sequencing dataset (Figure 3B), supporting the reliability of the transcriptomic analysis. Moreover, the higher treatment concentration generally produced greater changes in gene expression, indicating a concentration-responsive transcriptional effect. Among the validated genes, *HSPA6* and *GDF15* showed the most pronounced induction.

KEGG enrichment analysis revealed that the DEGs were significantly represented in several interconnected signaling networks, including the MAPK signaling pathway, cytokine–cytokine receptor interaction, TNF signaling pathway, PI3K–Akt signaling pathway, IL-17 signaling pathway, and NF-κB signaling pathway (Figure 3C). These pathways are broadly involved in cellular stress responses, inflammatory and cytokine signaling, proliferation, survival, and intercellular communication. The enrichment results were consistent with the altered expression of stress-responsive genes such as *HSPA6*, *HSPA1B*, *CHAC1*, and *GDF15*, as well as signaling-related genes including *TNFSF15* and *FOSL1*.

GO enrichment analysis (Figure 3D) showed that compound **6** reshaped transcription programs linked to gastric cancer malignant phenotypes, with all enriched GO terms closely correlated with tumor proliferation, metastasis and pro-tumor inflammatory microenvironment. Two proliferation-related biological processes were highly enriched: epithelial cell proliferation (22 DEGs, 10.14%) and regulation of epithelial cell proliferation (19 DEGs, 8.76%). Consistent with global downregulation of proliferative driver genes, this signature verified that compound **6** strongly inhibits abnormal hyperproliferation of AGS cells. Amoeboidal-type cell migration (21 DEGs, 9.68%), a key pathway governing gastric cancer invasion and metastasis, was also markedly enriched, implying an anti-metastatic transcriptional effect of compound **6**. The cytokine-mediated signaling pathway (21 DEGs, 9.68%) connected upstream inflammatory cascades (TNF, IL-17, NF-κB, MAPK) and downstream malignant phenotypes, revealing that compound **6** blocks pro-tumor cytokine crosstalk in AGS cells. Positive regulation of kinase activity (20 DEGs, 9.22%) covered oncogenic PI3K-Akt and MAPK cascades; altered expression of kinase regulators further confirmed the suppression of oncogenic signal transduction by compound **6**. Moderately enriched terms including angiogenesis, extracellular matrix remodeling and TNF response also participate in gastric cancer progression. At the molecular function layer, signaling receptor activator activity and receptor-ligand activity ranked first (30 DEGs each, 13.7%), followed by cytokine and growth factor-related functions. These results demonstrated that compound **6** disrupts ligand–receptor communication between tumor cells and inflammatory microenvironment to cut off exogenous pro-cancer signaling.

Collectively, these results demonstrate that compound **6** induced a reproducible and concentration-responsive transcriptional program in AGS cells. This response was characterized by alterations in genes related to proteostatic and redox stress, cytokine and receptor–ligand communication, epithelial growth regulation, cell migration, and extracellular remodeling. Rather than affecting a single isolated pathway, compound **6** produced coordinated changes across multiple signaling and phenotypic networks relevant to gastric cancer-cell biology. These transcriptomic findings provide a mechanistic framework for subsequent functional studies.

### 2.4. Effects of Compound ***6*** on Metabolic Levels of Gastric Cancer Cells

**Figure 4:**
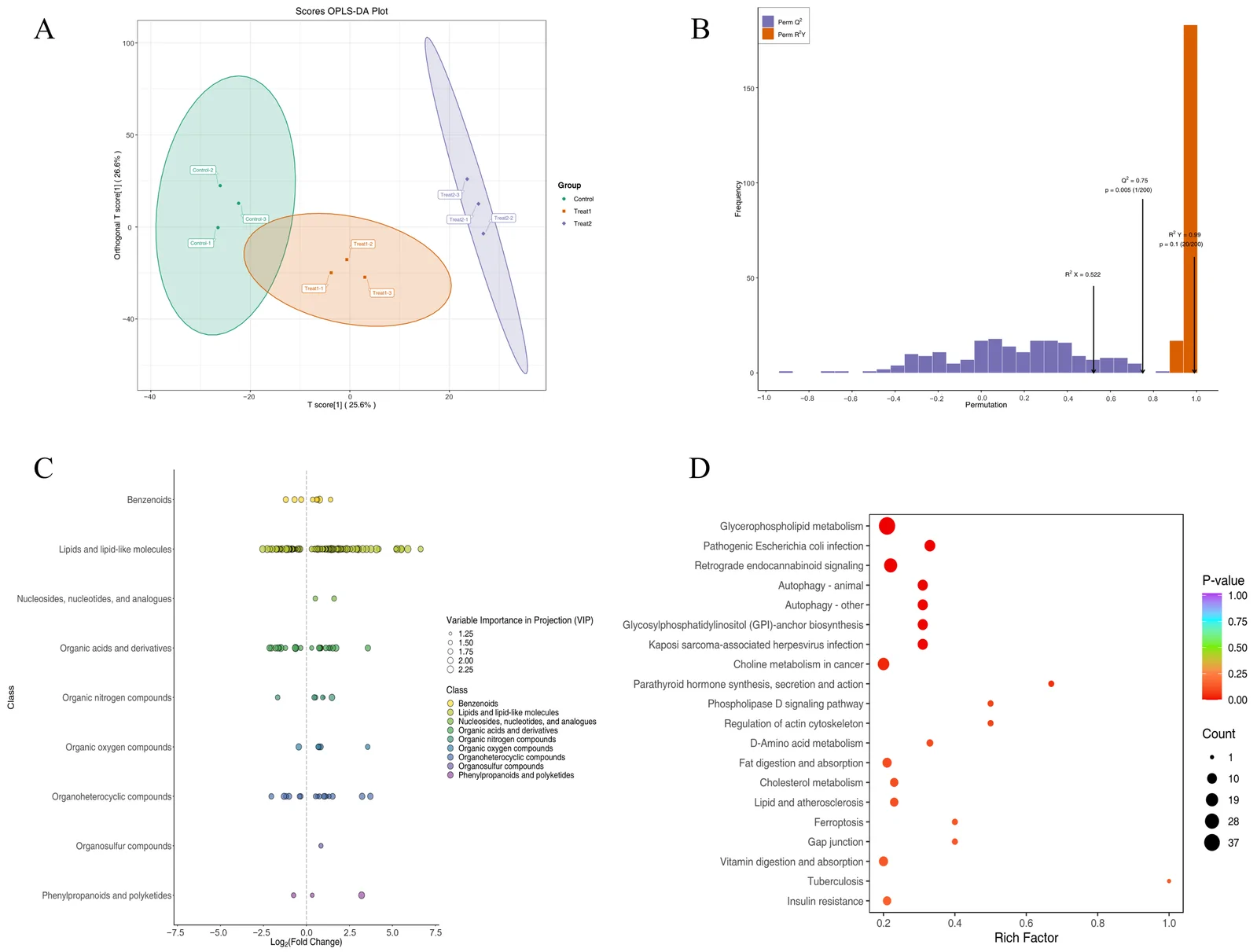
Untargeted metabolomic profiling of compound **6**-intervened AGS gastric cancer cells. (**A**) OPLS-DA score scatter plot for three experimental groups. The horizontal axis represents the predictive principal component, which reflects intergroup differences, the vertical axis corresponds to the orthogonal principal component, representing intra-group variations. Each dot in the plot stands for a single sample, and samples belonging to the same group are marked with identical colors, with Group denoting the sample grouping information. (**B**) Permutation test plot for OPLS-DA model verification. Orange bars represent the R^2^Y values of permuted models, purple bars denote the Q^2^ values of permuted models, and the values indicated by black arrows correspond to the R^2^X, R^2^Y and Q^2^ of the original OPLS-DA model. (**C**) Scatter plot of differential metabolites. Each dot corresponds to one metabolite, and distinct colors are assigned to different chemical classes. (**D**) Bubble plot of KEGG pathway enrichment for differential metabolites. The size of each bubble corresponds to the number of significantly differential metabolites enriched in the corresponding pathway.

Combined with the transcriptome sequencing results, untargeted metabolomics analysis was further performed in this study to evaluate the global metabolic profiles among groups via the OPLS-DA model, and permutation test was conducted to verify the reliability of the constructed model. The OPLS-DA score plot (Figure 4A) exhibited clear spatial separation among the Control, Treat 1 and Treat 2 groups, with tight intra-group aggregation of biological replicates. No extensive overlap was observed between the 95% confidence ellipses of distinct groups, indicating remarkable discrepancies in metabolic signatures across the three cohorts. The 200-times permutation test (Figure 4B) yielded model parameters of R^2^X = 0.522, R^2^Y = 0.99 and Q^2^ = 0.75. All permuted Q^2^ values were lower than the original Q^2^ of the model, accompanied by a permutation *p*-value of 0.005, which confirmed that the OPLS-DA model was free of overfitting and possessed robust discriminatory capacity.

Differential metabolites were screened with the threshold of VIP > 1 and visualized as a scatter plot categorized by chemical classes (Figure 4C). Lipids and lipid-like molecules represented the most abundant category with the largest number of high-VIP differential metabolites, containing both significantly upregulated and downregulated compounds. In addition, organoheterocyclic compounds and organic acids and derivatives harbored abundant differential metabolites, whereas only a small quantity of altered metabolites were identified in categories including benzenoids, phenylpropanoids and polyketides. These observations demonstrated that lipid species acted as core mediators driving intergroup metabolic variations.

KEGG pathway enrichment bubble plot (Figure 4D) identified glycerophospholipid metabolism as the most markedly perturbed metabolic pathway, featuring the highest Rich Factor, the greatest number of differential metabolites, and highly significant *p*-values. Several lipid homeostasis pathways tightly associated with tumor redox homeostasis and ferroptosis susceptibility were also significantly enriched, including choline metabolism in cancer, cholesterol metabolism, glycosylphosphatidylinositol (GPI)-anchor biosynthesis, as well as two autophagy subcategories (autophagy-animal, autophagy-other). Collectively, these metabolic signatures indicate that compound **6** primarily disrupts the glycerophospholipid metabolic network in AGS cells, accompanied by coordinated reprogramming of cholesterol turnover, membrane anchor lipid synthesis and autophagy flux. These metabolic shifts synergistically disrupt cellular lipid redox balance and suppress the proliferation of gastric cancer cells.

Consistent with the enrichment patterns of differentially expressed genes derived from transcriptome data, the above metabolomic findings collectively validated that the intervention triggered global lipid metabolic reprogramming centered on glycerophospholipid metabolism, and lipids and lipid-like molecules served as the pivotal differential biomarkers distinguishing the Control and Treat groups.

### 2.5. Effects of Compound ***6*** on Transcriptional and Metabolic Molecular Responses in Gastric Cancer Cells

**Figure 5:**
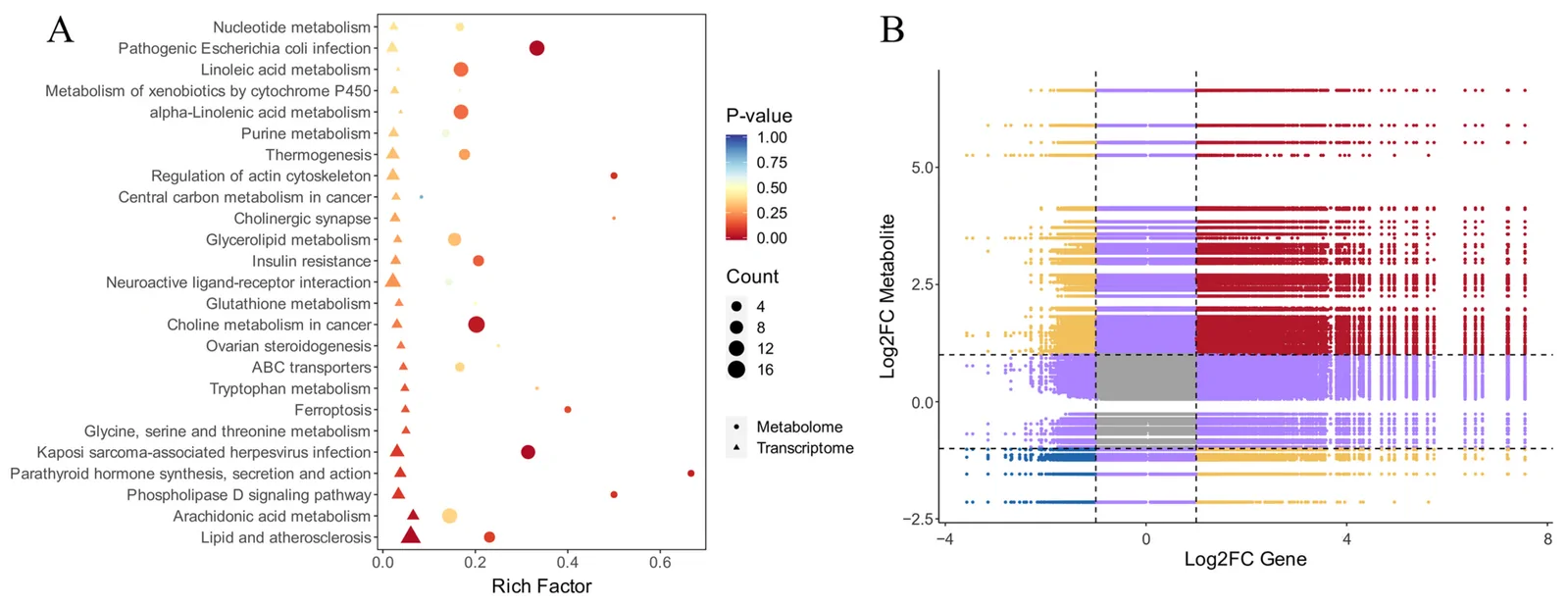
Integrated transcriptome–metabolome correlation analysis of AGS gastric cancer cells treated with compound **6**. (**A**) Combined KEGG enrichment bubble plot for DEGs and differential metabolites. Bubble shapes distinguish different omics layers, and bubble size reflects the number of differentially expressed genes or differential metabolites; larger bubbles indicate a greater count of differential molecules. (**B**) Nine-quadrant correlation scatter plot of gene-metabolite pairs. The graph is divided into nine quadrants numbered 1 to 9 sequentially from left to right and top to bottom. Each dot represents a single gene-metabolite correlation pair. The horizontal axis corresponds to the Log_2_FC of genes, and the vertical axis corresponds to the Log_2_FC of metabolites.

To systematically decipher the synergistic molecular responses at transcriptional and metabolic levels in gastric cancer cells intervened by compound **6**, integrated transcriptome-metabolome analysis was performed based on differentially expressed genes and differential metabolites. The KEGG pathway enrichment bubble plot (Figure 5A) revealed that multiple pathways associated with cell death, lipid metabolism and tumor metabolic reprogramming were significantly co-enriched in both omics datasets. The ferroptosis pathway annotation in the KEGG database, which represents the core program of iron-dependent regulated cell death, exhibited prominent enrichment signals in both transcriptomic and metabolomic profiles. This finding suggests that compound **6** may induce a ferroptosis-associated cellular state in tumor cells by disrupting the balance between lipid peroxidation and antioxidant defense. Co-enriched pathways including glutathione metabolism, arachidonic acid metabolism and linoleic acid metabolism are responsible for antioxidant homeostasis regulation and the generation of lipid peroxidation substrates, respectively, further verifying the activation of an oxidative stress-driven cell death regulatory network. In addition, the enrichment of tumor-specific metabolic reprogramming pathways such as central carbon metabolism in cancer and choline metabolism in cancer suggested that disrupted cellular energy metabolism indirectly facilitates tumor cell death by disturbing cell cycle progression and redox homeostasis.

The nine-quadrant correlation plot (Figure 5B) further uncovered the multi-layer regulatory landscape of the transcription-metabolism network in compound **6**-treated gastric cancer cells. Genes and metabolites distributed in Quadrant 5 showed no significant differential expression. Quadrants 2, 4, 6 and 8 contained pairs where only genes or metabolites were differentially altered, consistent with the characteristic of pathways exclusively enriched in a single omic layer. Pairs in Quadrants 3 and 7 shared identical differential expression patterns, representing positively correlated gene-metabolite pairs wherein transcriptional changes may positively regulate metabolite abundance. By contrast, pairs in Quadrants 1 and 9 displayed opposite variation trends, indicating negative regulatory relationships between genes and corresponding metabolites.

Collectively, the multi-omics integrated analysis indicates that compound **6** may trigger a ferroptosis-like phenotype in tumor cells and suppresses cell proliferation through the synergistic effects of ferroptosis activation, oxidative stress accumulation and metabolic reprogramming. Pathways related to lipid metabolism (e.g., linoleic acid metabolism), choline metabolism and ferroptosis exhibited highly overlapping enrichment signals in transcriptome and metabolome, which confirmed that the regulatory effects of compound **6** on gastric cancer cells were consistent at both transcriptional and metabolic levels, rather than random fluctuations observed in a single omic dataset.

## 3. Discussion

### 3.1. Sequential C3/C12 Derivatization Establishes a Modular Platform and Complementary Structure–Activity Relationships

The principal medicinal-chemistry advance of this study is the establishment of a sequential, site-differentiated strategy for expanding the chemical space of panaxadiol (PD). Previous PD optimization has focused mainly on the more accessible C3 hydroxyl group, although the biological consequences of derivatization are also influenced by the C12 substituent and the C17 side chain [19,20,21,22]. Here, a deliberately diversified C3 ester library was first constructed, after which a compact C3-acetyl scaffold was advanced to C12 functionalization. The unsuccessful attempts to introduce a bulky p-chlorobenzoyl linker and to chloroacetylate the proline-containing compound **6** were chemically informative: both outcomes define a practical steric boundary around the congested C12 hydroxyl group and show that the feasibility of C12 derivatization depends on both linker size and the architecture already installed at C3 (Figure S1). In the second stage, compound **1** was selected as the common C3-acetylated scaffold for C12 diversification after systematic evaluation of synthetic feasibility and biological activity across the entire C3 series. Although compounds **5** (glycine ester, IC_50_ = 17.14 μM) and **6** (proline ester, IC_50_ = 8.41 μM) also exhibited superior antiproliferative activity relative to PD, their C3 substituents introduced additional steric bulk around the triterpenoid core. Preliminary attempts to perform C12 chloroacetylation on compound **6** resulted in low conversion and complex product profiles, consistent with the severe steric congestion around the C12 reaction site. The glycine-derived compound **5**, while more compact than proline derivative **6**, still carried a larger side chain than the acetate group and introduced an additional Boc-protected amino moiety that could potentially interfere with subsequent nucleophilic substitution steps. In comparison, the C3 acetate group of compound **1** combined three favorable features: reliable and high-yielding synthesis, minimal steric encumbrance adjacent to the C12 hydroxyl group to ensure efficient subsequent functionalization, and substantially improved AGS-cell activity relative to the parent PD. This selection prioritized the robustness of the C12 diversification platform and the interpretability of C12-directed structure–activity relationships, rather than simply maximizing the activity of the starting scaffold. Conversion of its poorly reactive C12 hydroxyl group into the common electrophilic intermediate 10, followed by late-stage substitution with 13 piperazines, transformed a difficult site into a modular diversification node. By retaining a constant PD core, C3 acetate, and C12 oxyacetyl-piperazine linker, this focused congeneric series permitted the biological contribution of the distal substituent to be compared with substantially less structural confounding than would occur in a collection of unrelated analogues.

The antiproliferative data demonstrate that the platform was productive but strongly structure dependent. Thirteen of the 23 derivatives were more active than PD, nine showed IC_50_ values below 25 μM, and compounds **6**, **11**, and **17** entered the sub-10-μM range. Within the C3 series, compound **6** was approximately 4.6-fold more potent than PD and 2.3-fold more potent than acetate 1. Its activity is more reasonably attributed to the compact, conformationally restricted proline-derived geometry and a favorable steric-polar balance than to amino acid conjugation itself. The weaker activities of the phenylalanine-, tryptophan-, and extended lysine-derived analogues further show that increasing aromatic surface, side-chain length, or nominal polarity did not uniformly improve AGS-cell inhibition. This observation agrees with previous PD ester, halogenated, and nitrogen-containing derivative studies in which activity depended strongly on both substituent identity and modification site [20,21,22]. It is also consistent with panaxanediolone oxime derivatives that reduced normal-cell toxicity without consistently increasing tumor-cell antiproliferative activity, confirming that C3 modification is not intrinsically advantageous [23]. Because the amino groups in the present series remained Boc-protected and aqueous solubility was not measured, the improved activity of compound **6** should not be attributed to increased ionization or solubility. Although amino acid esterification can improve the physicochemical or pharmacokinetic properties of selected ginsengenin derivatives, such effects remain compound-specific and require direct measurement [24].

The C12 series provided a complementary and more discriminating structure–activity dimension. Intermediate 10 was inactive at concentrations up to 100 μM, demonstrating that chloroacetylation alone was insufficient to improve activity. Potency was restored only by selected terminal groups: the pyridylpiperazine derivative **11** and cyclohexylpiperazine derivative **17** improved activity by at least 19.7-fold and 14.7-fold, respectively, relative to intermediate 10, whereas phenyl-, benzhydryl-, and extended hydrophobic analogues remained weak or inactive. Chlorination of the pyridyl ring reduced potency by approximately 5.1-fold, defining a clear activity cliff and showing that a seemingly modest electronic and steric change can markedly alter the cellular response. These findings refine the general concept of dual-site modification. C12 is a useful diversification site, but its contribution is conditional on the size, shape, electronics, lipophilicity, and orientation of the terminal group rather than being intrinsically synergistic. This study therefore identifies three complementary lead directions: a conformationally restricted C3 ester represented by compound **6**, a heteroaryl piperazine represented by compound **11**, and a cyclic-alkyl piperazine represented by compound **17**.

### 3.2. Potency–Selectivity Balance Supports Purpose-Specific Lead Prioritization

Compounds **11** and **17** defined the potency ceiling of the current library, but their GES-1 concentration-response curves overlapped extensively with, and partly preceded, the concentrations required for AGS-cell inhibition. Compound **6** did not show unequivocal tumor-cell selectivity; nevertheless, it produced the clearest AGS/GES-1 response separation over the low-to-intermediate concentration range, including a limited window around 8 μM in which AGS inhibition was more pronounced while GES-1 viability was comparatively preserved. At higher concentrations, the curves converged, indicating that this advantage was relative and concentration-dependent rather than a broad therapeutic window. A similar potency-safety trade-off has been reported for halogenated PD derivatives, in which sub-μM activity against BGC-823 gastric cancer cells was accompanied by appreciable effects on GES-1 and other normal cells [22].

This comparison illustrates an important lead-development principle: the analogue with the lowest tumor-cell IC_50_ is not necessarily the most informative or developable candidate. The present library therefore yields a purpose-specific lead portfolio. Compound **11** serves as the potency benchmark, compound **17** provides a structurally distinct non-heteroaryl potency lead, and compound **6** offers the most balanced current platform for mechanism-oriented investigation. The choice of compound **6** for multi-omics analysis was thus based on integrated lead quality rather than potency alone, which strengthens the translational logic of this study. Nevertheless, the normal-cell comparison remains preliminary. Complete GES-1 IC_50_ values, confidence intervals, formal selectivity indices, and evaluation in additional nonmalignant gastric models are required. Moreover, because CCK-8 primarily reflects cellular reductive activity, the current findings should be confirmed using orthogonal proliferation, clonogenic-survival, and membrane-integrity assays, together with intracellular-exposure measurements. Rigorous mechanistic studies in gastric epithelial models similarly require protein-level, localization, genetic, and functional validation [25].

### 3.3. Compound ***6*** Induces a Coordinated Stress-, Cytokine-, and Growth-Regulatory Transcriptional Program

The transcriptomic analysis provides a reproducible molecular context for the cellular response to compound **6**. The 3.8:1 predominance of upregulated differentially expressed genes, concordant qPCR validation of nine representative genes, and generally larger responses at the higher treatment level indicate an inducible, concentration-responsive program rather than nonspecific transcriptional collapse. The validated genes are most appropriately interpreted as partially overlapping functional modules. *HSPA6* and *HSPA1B* are consistent with proteostatic stress; *CHAC1* and *GDF15* with amino-acid/redox stress and adaptive injury signaling; *FOSL1*, *TNFSF15*, and *DKK1* with growth- and cytokine-associated communication; and *MMP12* and *ANGPTL4* with extracellular remodeling and metabolic adaptation. This modular interpretation is more faithful to the data than assigning all changes to a single inflammatory pathway.

KEGG representation of MAPK, TNF, NF-κB, PI3K-Akt, IL-17, and cytokine-receptor pathways, together with GO terms related to epithelial proliferation, kinase regulation, migration, angiogenesis, adhesion, and extracellular organization, indicates that compound **6** perturbed interconnected networks relevant to gastric cancer-cell growth and environmental communication. A previous systematic review elaborated the multi-layered regulatory crosstalk among cell surface signal perception, extracellular matrix remodeling and migration-associated transcriptional programs [26]; nevertheless, these enrichments do not establish uniform activation or inhibition of the corresponding pathways, nor do they demonstrate direct suppression of migration or angiogenesis. MAPK–AMPK signaling can integrate oxidative and energetic stress with autophagy and ferroptosis susceptibility, but the direction and biological consequence of this integration depend on stimulus intensity, duration, and cellular context [27]. The marked induction of *HSPA6* and *GDF15* at the higher treatment level, together with the clearer separation of the high-dose metabolomic group, is consistent with a stronger cellular-stress state, although a temporal progression from adaptation to injury has not yet been demonstrated. Time-resolved measurement of ERK, JNK, p38, Akt, AMPK, and NF-κB activity, combined with pathway-specific perturbation, will be necessary to define direction and causality.

### 3.4. Membrane-Lipid and Redox Remodeling Supports a Testable Ferroptosis-Related Hypothesis

Accumulating evidence demonstrates that natural terpenoids can trigger multiple forms of regulated cell death in cancer cells, including apoptosis, ferroptosis, and autophagic cell death, through modulation of redox homeostasis and lipid metabolic networks [28]. In particular, a growing body of recent studies has shown that natural products and terpenoid derivatives can induce a ferroptosis-susceptible state in various tumor models by disrupting the cellular antioxidant defense system and promoting lipid peroxide accumulation [29]. Metabolomic profiling identified lipids and lipid-like molecules as the dominant differential class and revealed a graded displacement of the AGS-cell metabolic phenotype, most clearly at the higher treatment level. Enrichment of glycerophospholipid metabolism, choline metabolism in cancer, cholesterol metabolism, phospholipase D signaling, GPI-anchor biosynthesis, and autophagy-related annotations collectively points to remodeling of membrane composition and lipid turnover. This interpretation is biologically plausible because rapidly proliferating tumor cells require coordinated lipid synthesis, uptake, storage, and remodeling for membrane biogenesis, receptor organization, signaling, energy management, and protection from oxidative stress [30,31]. Importantly, individual lipid species changed in both directions. The data therefore support redistribution of lipid pools and altered membrane-lipid homeostasis rather than simple inhibition of lipid synthesis, generalized lipid depletion, or nonspecific metabolic collapse.

The integrated KEGG analysis further connected linoleic acid, α-linolenic acid, arachidonic acid, glycerolipid, choline, and glutathione metabolism with phospholipase D signaling and the ferroptosis annotation. The transcriptomic and metabolomic layers contributed unequally, with lipid pathways represented predominantly by differential metabolites and stress/cytokine processes more evident in the transcriptome. This asymmetry is biologically expected because gene expression and metabolite pools respond on different timescales and need not change proportionally. Likewise, the nine-quadrant analysis demonstrates concordant or discordant directional variation at the dataset level but cannot establish direct gene-to-metabolite regulation. The cross-layer convergence therefore strengthens the conclusion that membrane-lipid and redox homeostasis are central components of the compound **6** response without implying that every pathway was coordinately regulated at both levels.

Ferroptosis is defined by iron-dependent accumulation of lethal membrane lipid peroxides and is controlled by system x_c−/GSH/GPX4, FSP1/CoQ_10_, iron handling, and phospholipid-remodeling pathways [32,33]. The present data identify two features relevant to ferroptosis susceptibility: alteration in potentially oxidizable lipid pools and perturbation of glutathione-associated metabolism. However, they do not demonstrate iron dependence, lethal lipid peroxidation, or rescue by ferroptosis inhibitors. Autophagy, p62-dependent metabolic signaling, and mitochondrial dynamics may further influence this response through ferritin turnover, lipid-droplet mobilization, organelle quality control, and redox regulation [34,35]. Thus, the most defensible interpretation is that compound **6** generates a ferroptosis-related lipid/redox signature or sensitized state, not that ferroptotic cell death has already been established. Functional confirmation should prioritize C11-BODIPY lipid-ROS analysis, labile Fe^2+^, GSH/GSSG, MDA or 4-HNE, oxidized phospholipid profiling, and *GPX4*, *SLC7A11*, *ACSL4*, *TFRC*, and FSP1 measurements, together with rescue by ferrostatin-1 or liproxstatin-1 and parallel discrimination from apoptosis and necroptosis.

### 3.5. Integrated Working Model, Scientific Value, and Future Development

**Figure 6:**
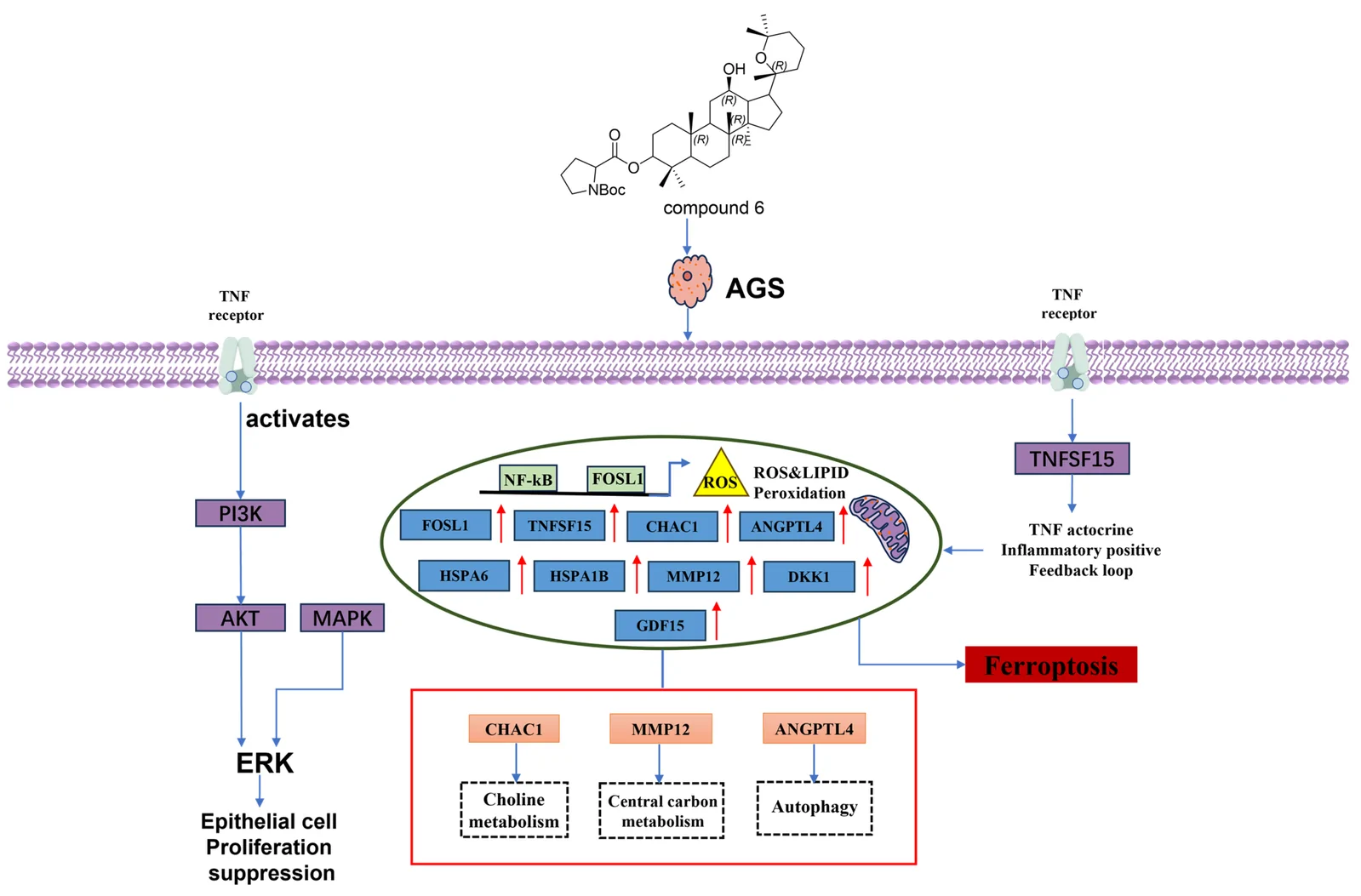
Proposed working model for the response of AGS cells to compound **6**.

The value of the integrated analysis lies in cross-layer triangulation rather than nominal validation of a linear mechanism. The transcriptome emphasized cellular stress, cytokine communication, growth regulation, and extracellular remodeling, whereas the metabolome emphasized phospholipid, choline, cholesterol, polyunsaturated-fatty-acid, and glutathione-associated changes. Their convergence around membrane-lipid and redox biology makes the working hypothesis more coherent than an interpretation based on either dataset alone. Figure 6 summarizes this evidence-based model while distinguishing observed molecular responses from literature-supported mechanistic links. The schematic should therefore be read as a framework for hypothesis testing: direct molecular targets, pathway direction, the sequence connecting stress signaling to lipid remodeling, and the final mode of regulated cell death remain unresolved.

This study provides four connected advances that define its broader value. First, it establishes a staged C3-to-C12 derivatization platform and experimentally identifies a steric boundary for C12 functionalization, rather than merely reporting a set of disconnected analogues. Second, it generates three structurally distinct sub-10-μM AGS-cell inhibitors and reveals clear activity cliffs, demonstrating that the PD scaffold supports discriminating and optimizable structure–activity relationships. Third, it separates maximal potency from preliminary normal-cell response and thereby introduces purpose-specific lead selection, with compound **6** selected as the most defensible mechanism-oriented candidate. Fourth, it connects medicinal chemistry with reproducible transcriptomic and metabolomic profiling to generate a falsifiable membrane-lipid/redox hypothesis. Together, these features provide substantially greater development value than synthesis, single-cell-line screening, or single-omics profiling alone.

Several limitations define the current evidence boundary. Activity was assessed in one gastric cancer cell line, and complete normal-cell selectivity, aqueous solubility, ester stability, permeability, intracellular exposure, metabolic stability, pharmacokinetics, and in vivo efficacy remain undetermined. The direct molecular target and the causal relationship among signaling, metabolic remodeling, and cell death also remain unresolved. Future work should therefore proceed in a prioritized sequence: first, confirm lead quality by verifying the antiproliferative activity of compound **6** in an additional 1–2 gastric cancer cell lines (MKN-45 and BGC-823), calculating formal selectivity indices, testing solubility and chemical stability, measuring intracellular drug exposure, and implementing orthogonal proliferation/death assays; second, systematically dissect the underlying molecular mechanism: perform Western blot to detect expression changes in phosphorylated MAPK cascade proteins (p-ERK, p-JNK, p-p38), and conduct canonical ferroptosis functional validation including C11-BODIPY lipid peroxidation detection and ferrostatin-1 rescue experiments; and third, evaluate translational feasibility by assessing the preliminary anti-tumor efficacy and biosafety of compound **6** in AGS xenograft mouse models, combined with spheroid/organoid assays and pharmacokinetic characterization. Within these limits, the principal conclusion is robust: sequential C3/C12 diversification converted PD into multiple low-μM AGS-cell inhibitors, and compound **6** reproducibly modulates cellular stress, cytokine signaling and membrane lipid redox homeostasis, providing a clear and experimentally verifiable strategy for the subsequent development of anti-gastric-cancer lead compounds.

## 4. Materials and Methods

### 4.1. Chemicals and Analytical Methods

All chemicals and solvents used in the experiments were purchased from commercial sources; unless otherwise stated, all chemicals and solvents were used without further purification. Panaxadiol (PD, HPLC purity ≥ 98%) was supplied by Beijing Ouhe Technology Co., Ltd. (Beijing, China). ^1^H NMR and ^13^C NMR spectra were recorded on a Bruker AV II-500 MHz nuclear magnetic resonance spectrometer (Bruker Corporation, Beijing, China). The ^1^H NMR spectra were acquired at a resonance frequency of 500 MHz with CDCl_3_ as the internal reference (δ 7.26 ppm), while the ^13^C NMR spectra were measured at 126 MHz, using CDCl_3_ as the internal standard (δ 77.16 ppm). High-resolution mass spectrometry (HRMS) analysis was performed on a Waters LCT Premier™ mass spectrometer (Waters Corporation, Beijing, China) equipped with an electrospray ionization (ESI) source. Optical rotations were recorded on a Rudolph Research Analytical AUTOPOL-IV polarimeter (Rudolph Research Analytical, Beijing, China).

All synthesized PD derivatives were stored as solid powders in tightly sealed vials at −20 °C under an atmosphere and protected from light, in order to minimize potential hydrolysis of ester derivatives (**1**–**9**) and oxidative degradation of piperazine-containing derivatives (**11**–**23**).

For biological assays, stock solutions were freshly prepared in anhydrous methanol on the day of each experiment, and serial working dilutions were prepared immediately before cell administration. All solution preparation procedures were completed at room temperature with minimal ambient exposure time to reduce the risk of compound degradation prior to cell incubation.

### 4.2. Synthetic Procedures

General Procedure for the Synthesis of C3-Substituted Panaxadiol Derivatives **1**–**9**: Under an argon atmosphere, panaxadiol (PD, 1.0 equiv.), the corresponding carboxylic acid—either acetic acid or an *N*-Boc-protected amino acid derivative (1.5 equiv.)—dicyclohexylcarbodiimide (DCC, 2.0 equiv.), and 4-(dimethylamino)pyridine (DMAP, 2.0 equiv.) were added to anhydrous dichloromethane (DCM; 0.05 M with respect to PD) at room temperature. The reaction mixture was heated to 40 °C and stirred for 24 h under an argon atmosphere. Reaction progress was monitored by thin-layer chromatography (TLC). After complete consumption of PD, the mixture was cooled to room temperature and then to 0 °C before being carefully quenched with deionized water. The phases were separated, and the aqueous phase was extracted with ethyl acetate (EtOAc, three times). The combined organic layers were washed with brine, dried over anhydrous sodium sulfate (Na_2_SO_4_), filtered, and concentrated under reduced pressure. The resulting residue was purified by silica gel column chromatography to afford the corresponding C3-substituted PD derivatives **1**–**9**.

Synthesis of Chloroacetyl Intermediate 10: Under an argon atmosphere, compound **1** (2.01 g, 4.00 mmol, 1.0 equiv.), calcium hydride (CaH_2_; 1.51 g, 35.82 mmol, 9.0 equiv.), tetrabutylammonium bromide (TBAB; 0.85 g, 2.63 mmol, 0.66 equiv.), and anhydrous toluene (40 mL) were added to a dry reaction flask at room temperature. The suspension was stirred for 10 min, after which chloroacetyl chloride (CAC, 2.98 g, 26.4 mmol, 6.6 equiv.) was added carefully. The reaction mixture was heated to 110 °C and stirred under reflux overnight. Reaction progress was monitored by TLC.

After complete consumption of compound **1**, the reaction mixture was cooled to room temperature and subsequently to 0 °C. Deionized water (40 mL) was then added slowly to quench the reaction. The organic phase was separated, and the aqueous phase was extracted with EtOAc (3 × 40 mL). The combined organic layers were washed with brine, dried over anhydrous Na_2_SO_4_, filtered, and concentrated under reduced pressure. The crude residue was purified by silica gel column chromatography to afford chloroacetyl intermediate 10.

General Procedure for the Synthesis of C12 Piperazine-Containing Derivatives **11**–**23**: Under an argon atmosphere, intermediate 10 (1.0 equiv.), the appropriate substituted piperazine derivative (1.5 equiv.), potassium carbonate (K_2_CO_3_, 2.5 equiv.), and potassium iodide (KI, 0.05 equiv.) were suspended in anhydrous acetonitrile (MeCN; 0.05 M with respect to intermediate 10) at room temperature. The reaction mixture was heated to 82 °C and stirred under reflux for 5 h. Reaction progress was monitored by TLC. Upon completion, the mixture was cooled to room temperature and concentrated under reduced pressure to remove most of the MeCN. The residue was diluted with water and EtOAc, and the resulting phases were separated. The aqueous phase was extracted with EtOAc three times. The combined organic layers were washed with brine, dried over anhydrous Na_2_SO_4_, filtered, and concentrated under reduced pressure. The crude products were purified by silica gel column chromatography to afford the corresponding C3/C12 dual-modified PD derivatives **11**–**23**.

The structures of all synthesized derivatives were confirmed by ^1^H nuclear magnetic resonance spectroscopy (^1^H NMR), ^13^C nuclear magnetic resonance spectroscopy (^13^C NMR), and high-resolution mass spectrometry (HRMS)**.** Detailed spectroscopic and mass-spectrometric data are provided in the Supplementary Materials.

### 4.3. Evaluation of Anti-Gastric Cancer Activity

The human gastric cancer AGS cell line was purchased from the Cell Bank of the Chinese Academy of Sciences (Shanghai, China).

AGS cells were cultured in high-glucose Dulbecco’s Modified Eagle Medium (DMEM, Bio-Channel, Nanjing, China) supplemented with 1% penicillin–streptomycin double antibody solution (Solarbio, Beijing, China) and 10% fetal bovine serum (FBS, HyClone, Logan, UT, USA), and incubated in a cell incubator (Heal Force, Shanghai, China) at 37 °C with 5% CO_2_ atmosphere. Stock solutions of test compounds were prepared with methanol as the solvent at a concentration of 5 mM. Each stock solution was serially diluted with complete DMEM medium to obtain final working concentrations of 2, 4, 8, 16, 32, and 64 μM. For the highest working concentration of 64 μM, the stock solution was diluted approximately 78-fold, corresponding to a final methanol volume fraction of 1.28% (*v*/*v*) in the culture wells. A solvent control group containing 1.28% (*v*/*v*) methanol (matching the methanol content in the highest-concentration treatment group) was set up to evaluate the potential cytotoxicity of the solvent. As illustrated in Figures S2–S24, methanol at this concentration exerted no obvious adverse effects on the survival and growth of tested cells. Since all lower-concentration treatment groups had methanol volume fractions below 1.28%, it can be confirmed that the methanol solvent did not introduce cytotoxicity or interfere with the results of the CCK-8 antiproliferative assay.

For the anti-gastric cancer activity assay, AGS cells were seeded into 96-well plates at a density of 5 × 10^3^ cells per well in 100 μL complete medium and incubated at 37 °C for 24 h. Serial dilutions of test compounds (final concentrations ranging from 2 μM to 64 μM) prepared in maintenance medium were added to wells containing adherent AGS monolayers. Control wells were treated with maintenance medium without test compounds.

After incubation for 48 h at 37 °C under 5% CO_2_, 10 μL Cell Counting Kit-8 (CCK-8, Puxitang Biotech Co., Ltd., Beijing, China) reagent was added to each well. The plates were incubated for another 2 h at 37 °C in a 5% CO_2_ incubator. The absorbance (OD value) at 450 nm was detected using a microplate reader, and cell viability was calculated according to the following formula:

Cell viability (%) = [(OD experimental − OD blank)/(OD control − OD blank)] × 100%

Statistical comparisons among groups were performed via one-way analysis of variance (one-way ANOVA) followed by a *t*-test using GraphPad Prism 6.0 software. Differences with *p* < 0.05 were considered statistically significant. All IC_50_ values are presented as mean ± standard deviation (SD) from three independent biological experiments, each performed with three technical replicates. IC_50_ values and 95% confidence intervals were calculated by nonlinear regression of concentration–viability curves.

### 4.4. Multi-Omics Analysis

#### 4.4.1. Sample Preparation

According to the concentration–response fitting curve of compound **6** (Figure 2), 5 μM corresponds to a sublethal concentration with a cellular viability of approximately 70%, while 8 μM is close to the half-maximal inhibitory concentration (IC_50_), yielding a cell survival rate of roughly 50%. These two concentrations were adopted for subsequent mechanistic investigations, as they enable the detection of early molecular regulatory events triggered by the drug and produce a remarkable phenotypic inhibition of cell proliferation. On the basis of the above results, three experimental groups were established using gastric cancer AGS cells: a blank control group treated with culture medium alone, Treatment Group 1 incubated with medium supplemented with 5 μM compound **6**, and Treatment Group 2 incubated with medium containing 8 μM compound **6**. After 48 h of drug treatment, the culture medium was aspirated, and the cell monolayer was swiftly rinsed two to three times with ice-cold phosphate-buffered saline (PBS). An appropriate volume of ice-cold PBS was added, and adherent cells were gently scraped with a cell scraper before being transferred into centrifuge tubes. The cell suspension was centrifuged at 300–500× *g* for 5 min at 4 °C, and the supernatant was discarded. The cell pellet was washed with ice-cold PBS and subjected to a second centrifugation at 1000× *g* for 5 min at 4 °C, followed by supernatant removal. The cell pellet was resuspended in PBS for cell counting. An aliquot containing 1 × 10^7^ cells was transferred into a 2 mL sterile centrifuge tube, which was then centrifuged at 1000× *g* for 10 min at 4 °C. The supernatant was discarded, and the cell precipitate was reserved for subsequent experiments.

#### 4.4.2. Sample Processing

Metabolomic Samples: A total of 500 μL of 80% aqueous methanol extraction solution containing internal standard was added to the prepared samples, followed by vortexing for 3 min to fully suspend the samples. The centrifuge tubes were snap-frozen in liquid nitrogen for 5 min, thawed on dry ice for 5 min, and then thawed on ice for another 5 min, with vortex mixing for 2 min afterwards. The freeze–thaw-vortex cycle was repeated three times. The mixture was centrifuged at 12,000 r/min for 10 min at 4 °C, and 300 μL of the supernatant was transferred to correspondingly numbered centrifuge tubes. After standing at −20 °C for 30 min, the samples were centrifuged again at 12,000 r/min for 3 min at 4 °C. A volume of 200 μL supernatant was transferred into the corresponding liner tubes of sample vials for subsequent analysis.

Transcriptomic Samples: Total RNA was extracted from samples using the guanidinium isothiocyanate-phenol method, and the purified RNA was dissolved in DEPC-treated water. RNA quantification was accurately performed using a Qubit 4 fluorometer (Thermo Fisher Scientific, Waltham, MA, USA), and RNA integrity (RQN value) was assessed via the Qsep400 high-throughput biofragment analyzer (BiOptic Inc., New Taipei City, Taiwan, China) to guarantee that sample quality met the library construction criteria.

#### 4.4.3. Public Omics Data Deposition

RNA-seq transcriptomic raw sequencing data derived from compound **6**-treated human AGS gastric adenocarcinoma cells were deposited in the NCBI BioProject database with the accession number PRJNA1503497, including nine biological replicates with matched BioSample identifiers and individual accessible links: Control-1 (SAMN62016112, https://www.ncbi.nlm.nih.gov/biosample/62016112 (accessed on 1 September 2026)), Control-2 (SAMN62016113, https://www.ncbi.nlm.nih.gov/biosample/62016113 (accessed on 1 September 2026)), Control-3 (SAMN62016114, https://www.ncbi.nlm.nih.gov/biosample/62016114 (accessed on 1 September 2026)), Treat1-1 (SAMN62016115, https://www.ncbi.nlm.nih.gov/biosample/62016115 (accessed on 1 September 2026)), Treat1-2 (SAMN62016116, https://www.ncbi.nlm.nih.gov/biosample/62016116 (accessed on 1 September 2026)), Treat1-3 (SAMN62016117, https://www.ncbi.nlm.nih.gov/biosample/62016117 (accessed on 1 September 2026)), Treat2-1 (SAMN62016118, https://www.ncbi.nlm.nih.gov/biosample/62016118 (accessed on 1 September 2026)), Treat2-2 (SAMN62016119, https://www.ncbi.nlm.nih.gov/biosample/62016119 (accessed on 1 September 2026)), and Treat2-3 (SAMN62016120, https://www.ncbi.nlm.nih.gov/biosample/62016120 (accessed on 1 September 2026)). Untargeted metabolomics raw data generated from identical cell samples were submitted to the EMBL-EBI MetaboLights public repository under study ID MTBLS15239, and the full metabolomics dataset can be accessed via the URL https://www.ebi.ac.uk/metabolights/MTBLS15239 (accessed on 1 September 2026). All cell samples used for multi-omics profiling were *Homo sapiens* gastric adenocarcinoma AGS cells with Tax ID 9606, collected in Jinan, Shandong; the RNA-seq datasets were submitted to NCBI on 28 July 2026, while the untargeted metabolomics data were deposited into MetaboLights on 3 August 2026.

### 4.5. qPCR Validation

Each group of cells served as one biological replicate, with three independent biological replicates performed in total, and three technical replicates set for each qPCR reaction.

RNA was extracted from transcriptome samples to determine RNA concentration and purity. Reverse transcription was carried out using 2 μg of RNA in a total reaction volume of 10 μL, consisting of 8 μL master mix, 1 μL primer mix, and 1 μL diluted cDNA. Three technical replicates were prepared for each sample.

Target genes included *FOSL1*, *TNFSF15*, *CHAC1*, *HSPA6*, *HSPA1B*, *MMP12*, *DKK1*, *GDF15* and *ANGPTL4*. *β-actin* was used as the reference gene. The primer sequences used in this experiment are listed in Table 2. The 2^−ΔΔCt^ method was adopted to calculate the relative gene expression levels. One-way analysis of variance (one-way ANOVA) was applied for statistical analysis, and a *p*-value < 0.05 was considered statistically significant.

## 5. Conclusions

In this study, we developed a novel stepwise, site-differentiated strategy for panaxadiol structural optimization, which enables sequential selective C3 esterification and late-stage C12 diversification via a chloroacetyl–piperazine linker and supports systematic complementary structure–activity relationship analysis of the two positions. Among the 23 synthesized derivatives, compound **6** was identified as a promising lead candidate with micromolar-level antiproliferative activity against gastric cancer cells and relative safety to non-malignant gastric epithelial cells within a defined concentration range. Integrated transcriptomic and metabolomic analyses reveal that the antitumor effect of compound **6** is associated with stress and cytokine signaling modulation, membrane lipid remodeling, and induction of a ferroptosis-related redox state. While this study has limitations in cell model coverage and mechanistic validation depth, follow-up work will expand cell panels, perform functional validation of the ferroptosis mechanism, and evaluate in vivo efficacy. This study lays a solid chemical and biological foundation for the development of novel anti-gastric cancer agents derived from natural triterpenoids, and holds important scientific value and application prospects for advancing the research and development and clinical translation of bioactive natural products.

## Figures and Tables

**Table 1:** Antiproliferative activity of PD derivatives against AGS gastric cancer cells. IC_50_ values marked with “>100” are extrapolated from nonlinear regression curves, as 50% cell growth inhibition was not achieved within the experimentally tested concentration range (2–64 μM). Percentage inhibition of these low-activity compounds at the highest tested concentration (64 μM) is provided in Table S1 of the Supplementary Materials.

Compound	IC_50_ (μM)	95% Confidence Interval (μM)	Compound	IC_50_ (μM)	95% Confidence Interval (μM)
**PD**	38.29 ± 6.31	31.98–44.59	**13**	>100	-
**1**	19.54 ± 1.66	17.88–21.20	**14**	17.26 ± 0.94	16.32–18.19
**2**	66.17 ± 3.93	62.24–70.10	**15**	74.86 ± 9.59	65.27–84.44
**3**	32.72 ± 4.94	27.78–37.65	**16**	64.31 ± 15.47	48.84–79.78
**4**	26.92 ± 9.51	17.41–36.42	**17**	6.78 ± 1.07	5.71–7.86
**5**	17.14 ± 2.76	14.38–19.90	**18**	15.42 ± 1.20	14.22–16.62
**6**	8.41 ± 0.61	7.81–9.02	**19**	26.06 ± 3.82	22.24–29.88
**7**	49.62 ± 4.38	45.24–53.99	**20**	15.27 ± 3.17	12.10–18.44
**8**	34.57 ± 9.73	24.84–44.30	**21**	91.08 ± 17.22	73.86–108.30
**9**	>100	-	**22**	60.27 ± 7.76	52.51–68.02
**10**	>100	-	**23**	24.24 ± 2.64	21.60–26.87
**11**	5.08 ± 0.13	4.94–5.21	5-Fu	14.42 ± 1.52	12.90–15.94
**12**	>100	-			

**Table 2:** Sequence of primers for the qPCR analysis.

Gene	Forward Sequence (5′ → 3′)	Reverse Sequence (5′ → 3′)
*FOSL1*	CAGGCGGAGACTGACAAACTG	TCCTTCCGGGATTTTGCAGAT
*TNFSF15*	GCACCTCTTAGAGCAGACGG	CGGAATGTGACCTGGGAGTAAAT
*CHAC1*	GAACCCTGGTTACCTGGGC	CGCAGCAAGTATTCAAGGTTGT
*HSPA6*	CAAGGTGCGCGTATGCTAC	GCTCATTGATGATCCGCAACAC
*HSPA1B*	TTTGAGGGCATCGACTTCTACA	CCAGGACCAGGTCGTGAATC
*MMP12*	CATGAACCGTGAGGATGTTGA	GCATGGGCTAGGATTCCACC
*DKK1*	CCTTGAACTCGGTTCTCAATTCC	CAATGGTCTGGTACTTATTCCCG
*GDF15*	GACCCTCAGAGTTGCACTCC	GCCTGGTTAGCAGGTCCTC
*ANGPTL4*	GTCCACCGACCTCCCGTTA	CCTCATGGTCTAGGTGCTTGT

## Data Availability

The original contributions presented in this study are included in this article/Supplementary Materials. Further inquiries can be directed to the corresponding author.

## Supplementary Materials

The following supporting information can be downloaded at https://www.mdpi.com/article/10.3390/molecules31183225/s1, Figure S1: Screening of experimental conditions; Table S1: Percentage inhibition of low-activity panaxadiol derivatives against AGS gastric cancer cells at the highest tested concentration (64 μM); Figures S2–S24: Determination of cellular activity of compounds **1**–**23**; Figure S25–S70: ^1^H, ^13^C spectrum of compounds **1**–**23**.
