# Supplementary material for: Design, Synthesis, and Antiproliferative Evaluation of C3/C12-Modified Panaxadiol Derivatives Against Gastric Cancer Cells: Integrated Transcriptomic and Metabolomic Analyses
Source: Molecules. 2026 Sep 12;31(18):3225. doi: 10.3390/molecules31183225 (PMC13609390; doi:10.3390/molecules31183225)

SUPPORTING INFORMATION

Design, Synthesis, and Antiproliferative Evaluation of C3/C12-Modified Panaxadiol Derivatives against Gastric Cancer Cells: Integrated Transcriptomic and Metabolomic Analyses

Yueru Zhang [1,2,#], Hongqing Xie [1,#], Chenggang Shan [1,#], Jinlong Han [1], Fangzhou Zhao [1], Xianchang Wang [1], Yinan Qi[1,2], Xiaoyang Li[3], Jianhua Zhang[1], Feng Zhang[1] and Yun Zhou [1,*]

1 Institute of Industrial Crops, Shandong Academy of Agricultural Sciences, Jinan 250100, China; 13553169667@163.com (Y.Z.); xiehongqing@zju.edu.cn (H.X.); shanchenggang@126.com (C.S.); goldendragonh@163.com (J.H.); 19508465248@163.com (F.Z.) archangw@163.com (X.W.); 19862613161@163.com (Y. Q.); zhangjianhua198904@163.com (J.Z.); zhangfeng 0723 @12 6.com (F.Z.)

2 School of Pharmacy, Shandong University of Traditional Chinese Medicine, Jinan 250355, China; 13553169667@163.com (Y.Z.); 19862613161@163.com (Y. Q.)

3 Department of Applied Chemistry, China Agricultural University, Beijing 100193, China; b20233100844@cau.edu.cn (X.L.)

# Yueru Zhang, Hongqing Xie and Chenggang Shan contributed equally to this work.

* Correspondence: zhouyun@saas.ac.cn (Y.Z.)

Table of Contents

1. Screening of experimental conditions..S3

2. Percentage inhibition of low-activity panaxadiol derivatives against AGS gastric cancer cells at the highest tested concentration (64 μM)..S4

3. Activity Assay of PD Derivatives..S5

4. Synthesis of procedure...S17

5. ^{1}H, ^{13}C spectra of the products...S26

1. Screening of experimental conditions

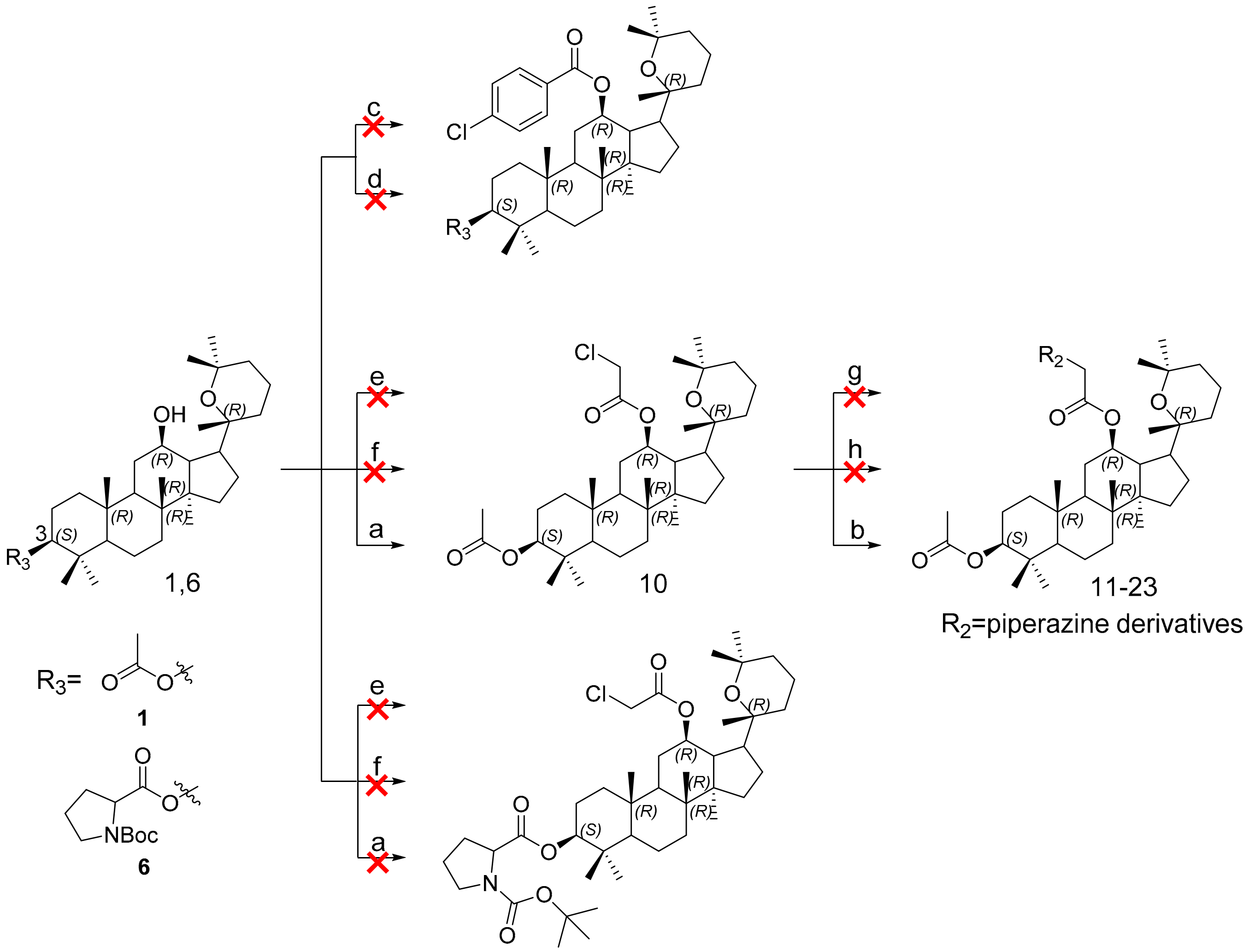

Figure S1. Scheme for synthetic exploration at the C12 position of panaxadiol (PD). (a) $PhCH_3$, TBAB, CaH_2, 110 ℃, overnight; (b) K_2CO_3, KI, MeCN, 82 ℃, 5 h; (c) DCM, Et_3N, DMAP, p-CBC, 40 ℃, 4-24 h; (d) LiHDMS, THF, p-CBC, -78 ℃ to rt, 4-24 h; (e) DCM, Et_3N, DMAP, CAC, 40 ℃, 4-24 h; (f) DCM, DCC, DMAP, CAC, 40 ℃, 4-24 h; (g) DMAP, dioxane, piperazine derivatives, 40 ℃, 4-24 h; (h) K_2CO_3, ACE, piperazine derivatives, 60 ℃, 4-24 h.

2. Percentage inhibition of low-activity panaxadiol derivatives against AGS gastric cancer cells at the highest tested concentration (64 μM)

Table S1. Percentage inhibition of low-activity panaxadiol derivatives against AGS gastric cancer cells at the highest tested concentration (64 μM)

Compound	Inhibition rate at 64 μM (%)
**9**	**31.72%**
**10**	**23.96%**
**12**	**5.46%**
**13**	**0.00%**

3. Activity Assay of PD Derivatives

Figure S2. Determination of cellular activity of Compound 1

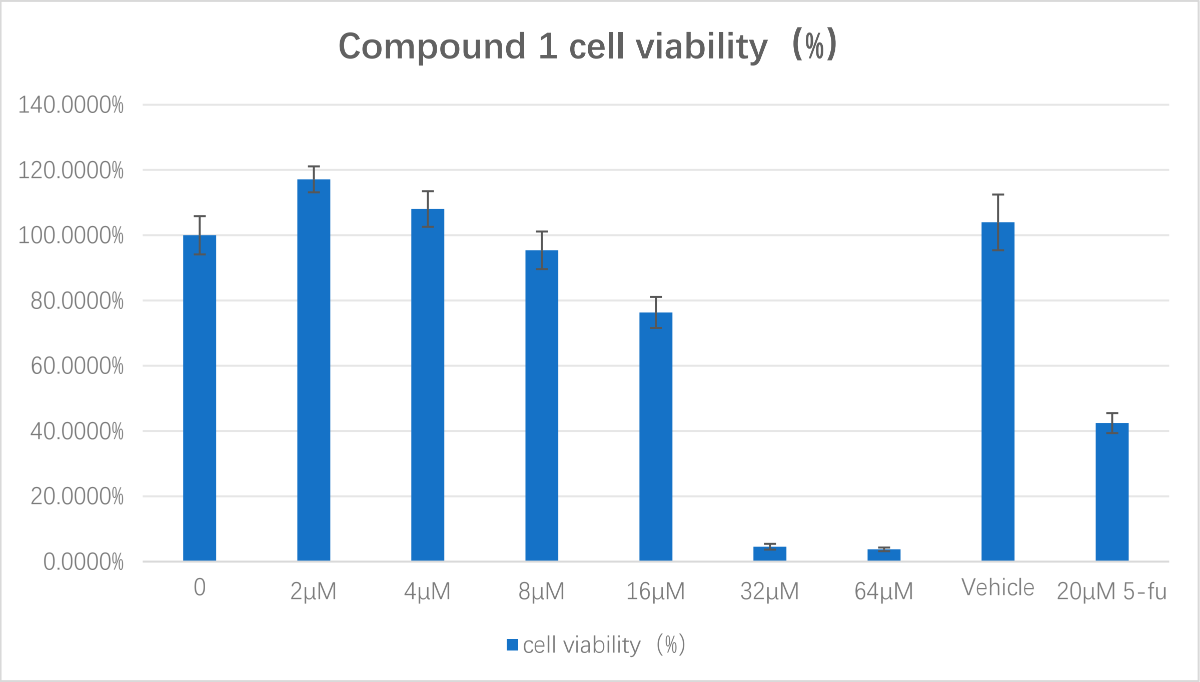

Figure S3. Determination of cellular activity of Compound 2

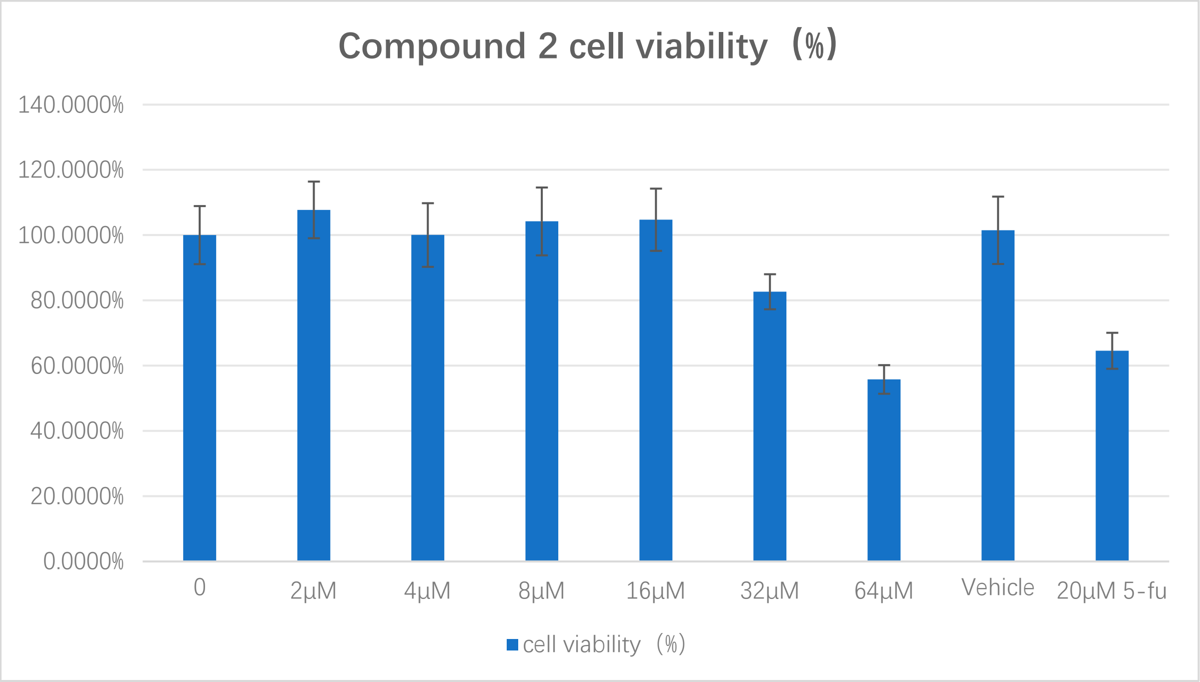

Figure S4. Determination of cellular activity of Compound 3

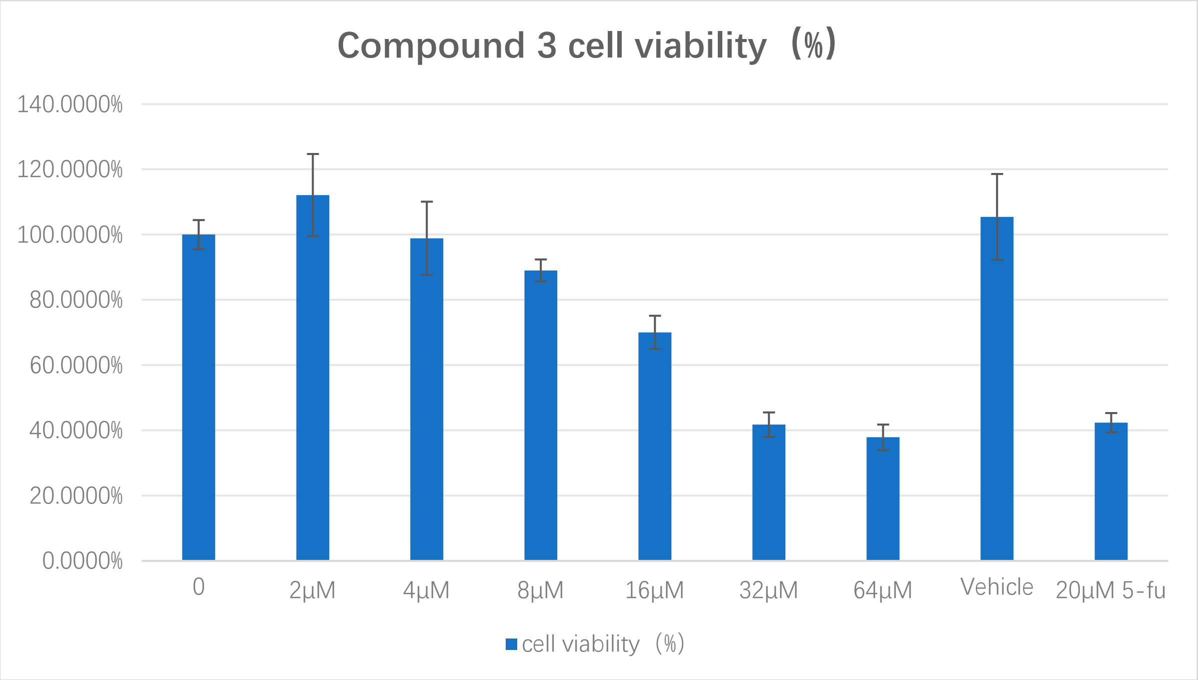

Figure S5. Determination of cellular activity of Compound 4

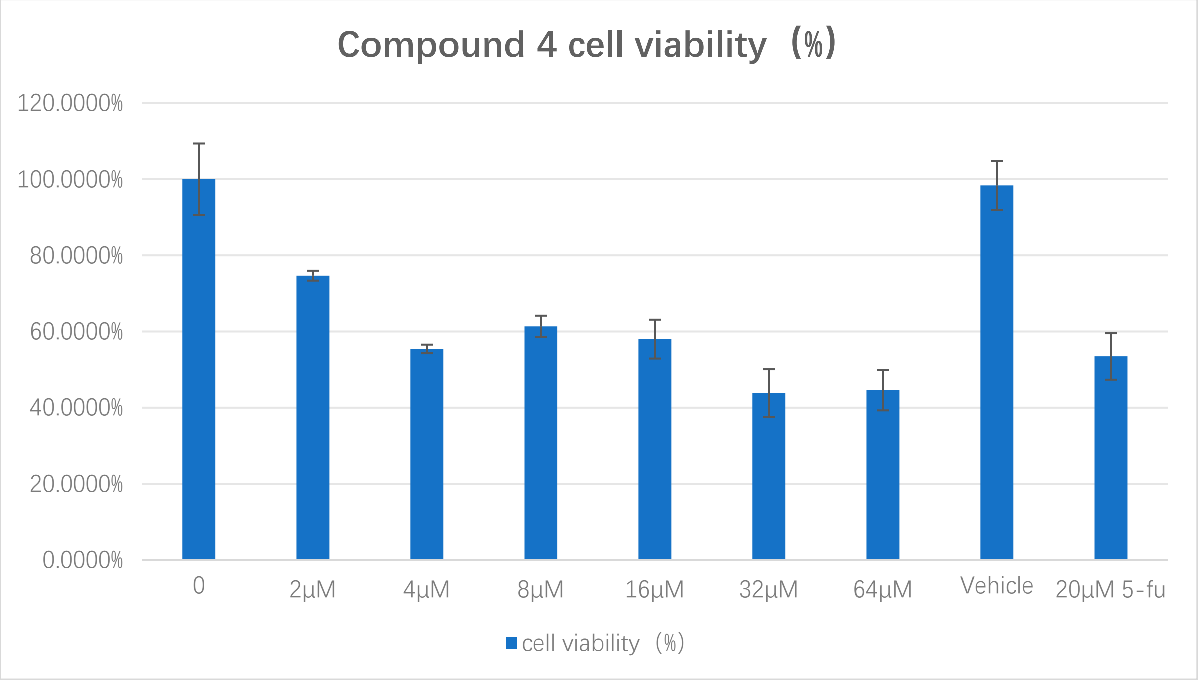

Figure S6. Determination of cellular activity of Compound 5

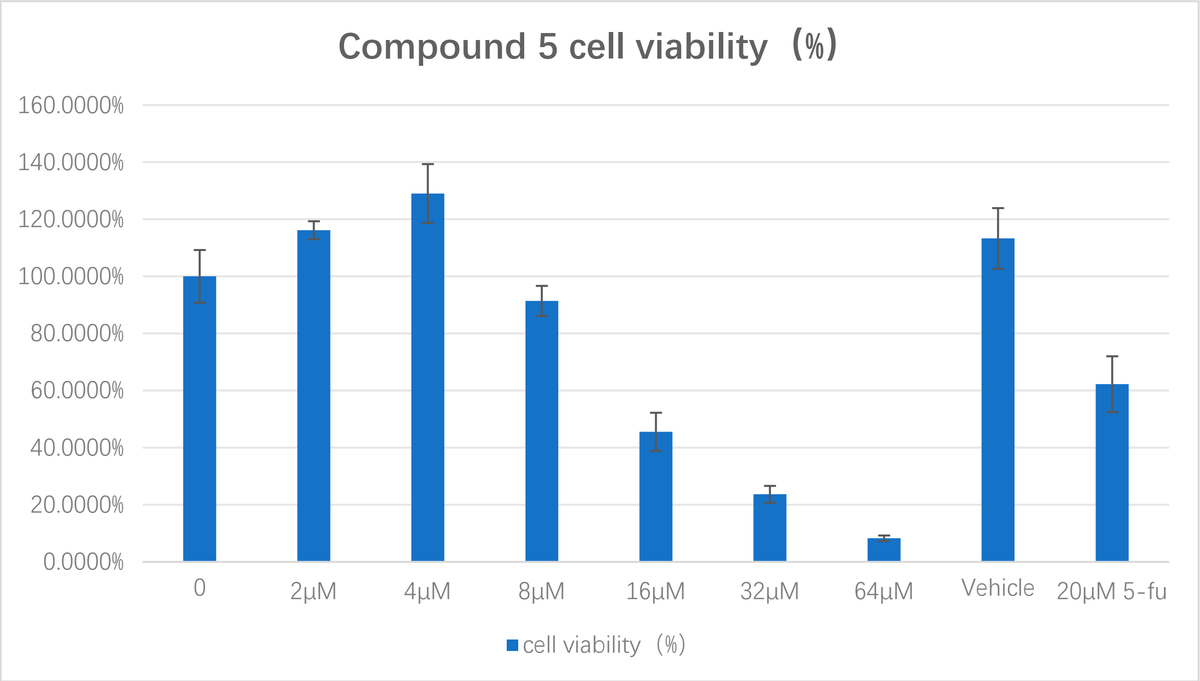

Figure S7. Determination of cellular activity of Compound 6

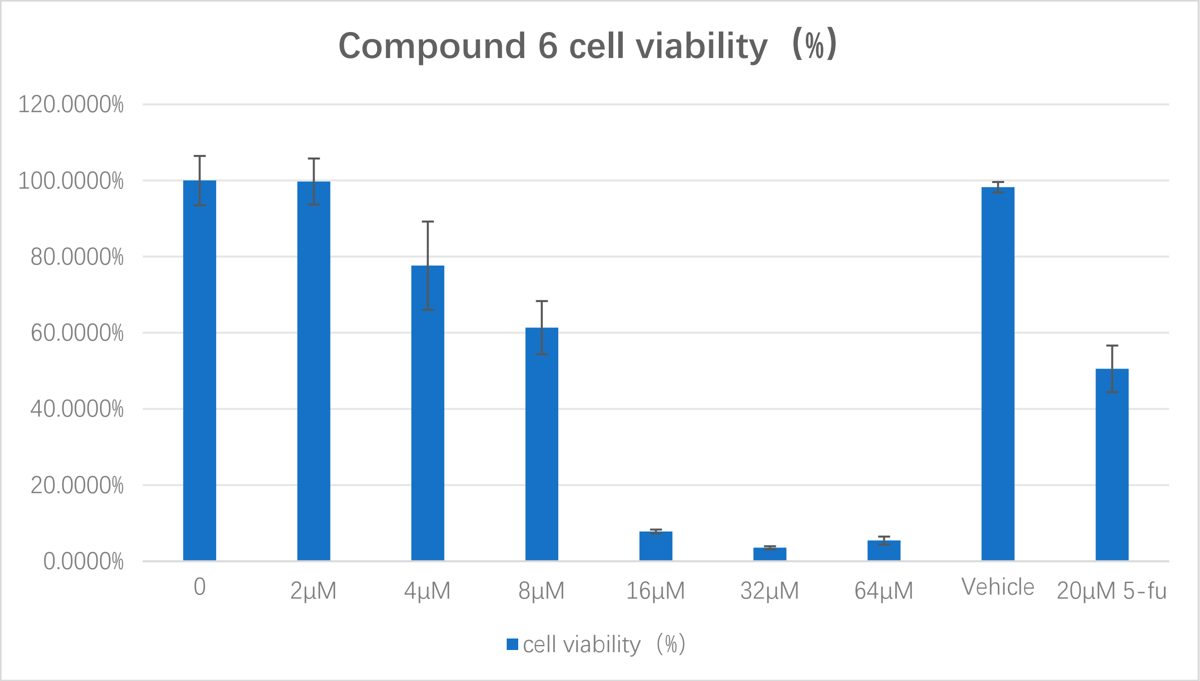

Figure S8. Determination of cellular activity of Compound 7

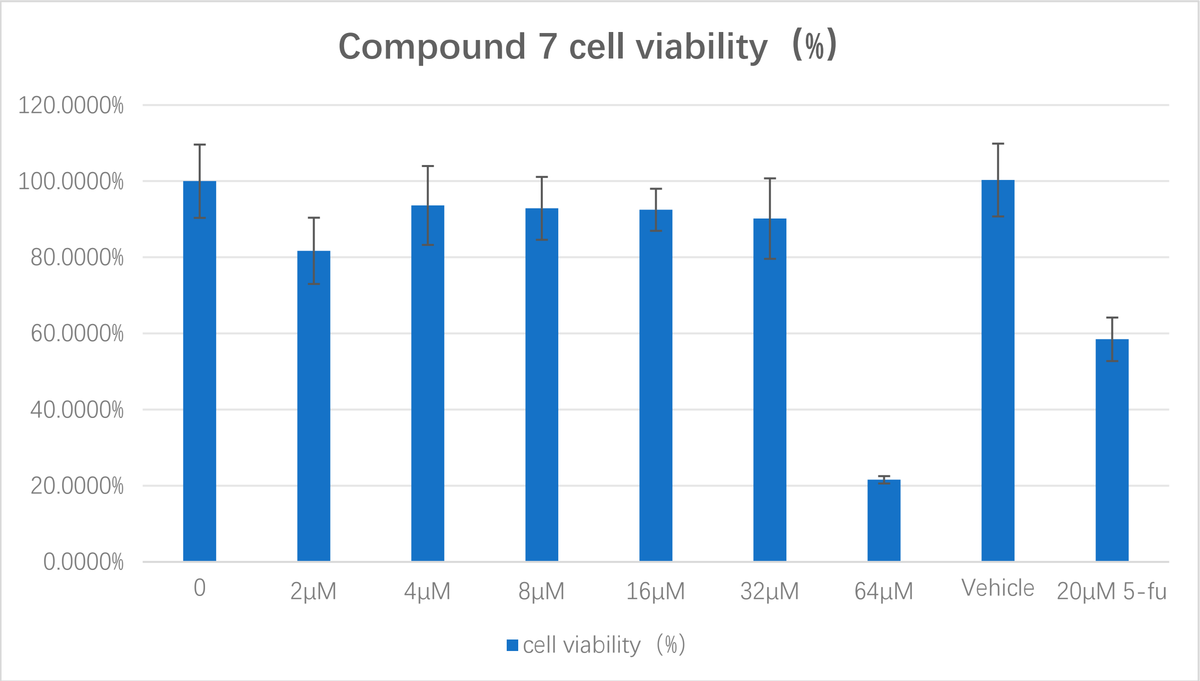

Figure S9. Determination of cellular activity of Compound 8

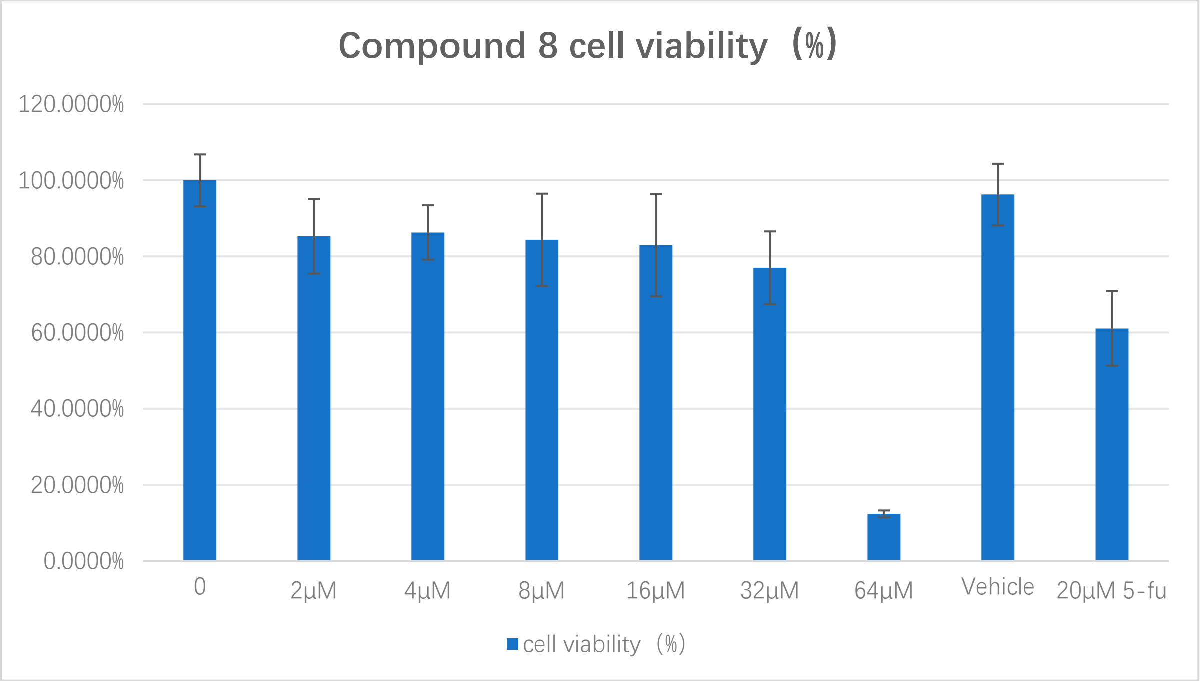

Figure S10. Determination of cellular activity of Compound 9

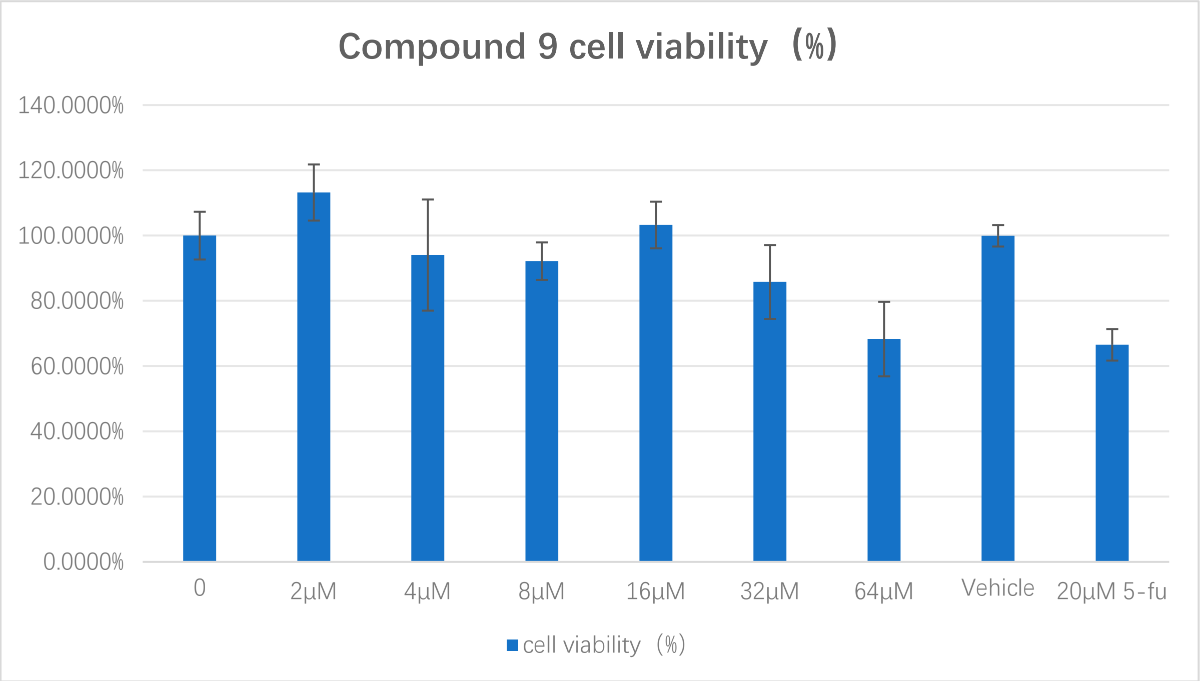

Figure S11. Determination of cellular activity of Compound 10

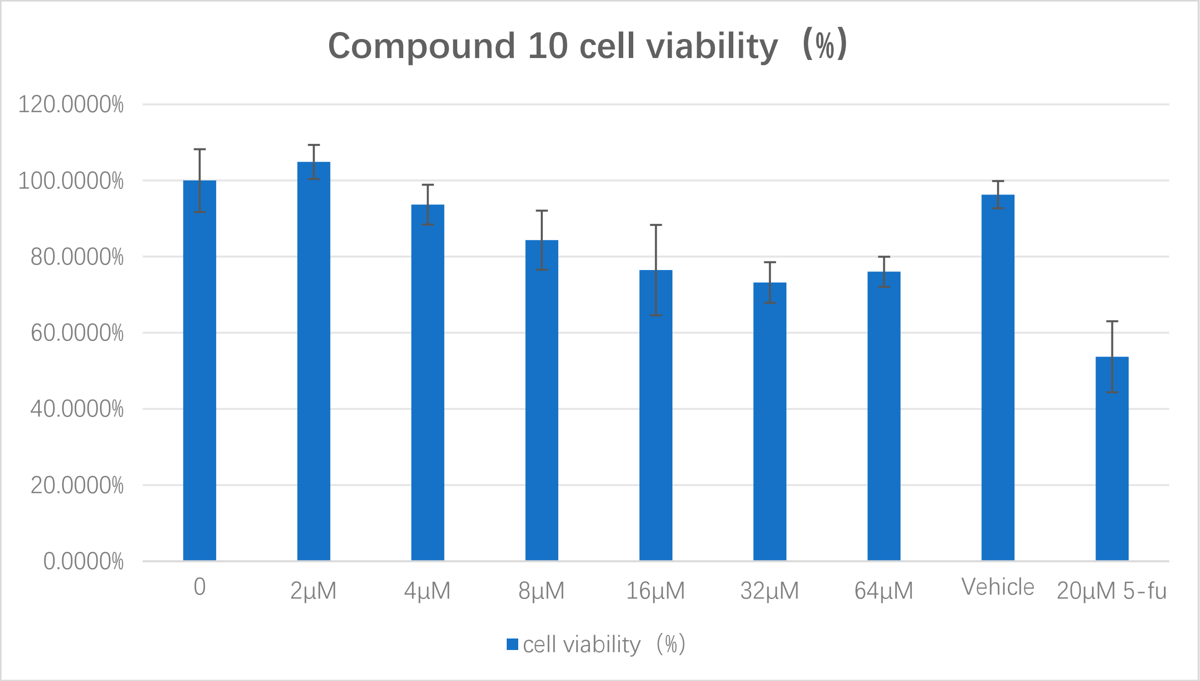

Figure S12. Determination of cellular activity of Compound 11

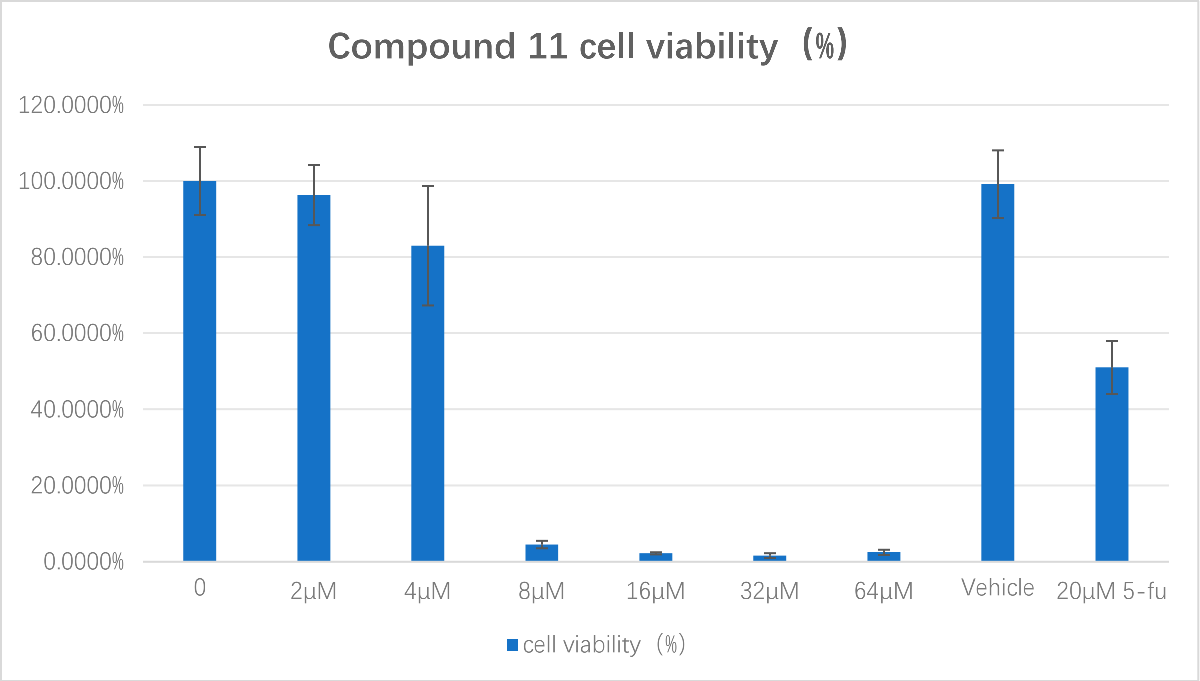

Figure S13. Determination of cellular activity of Compound 12

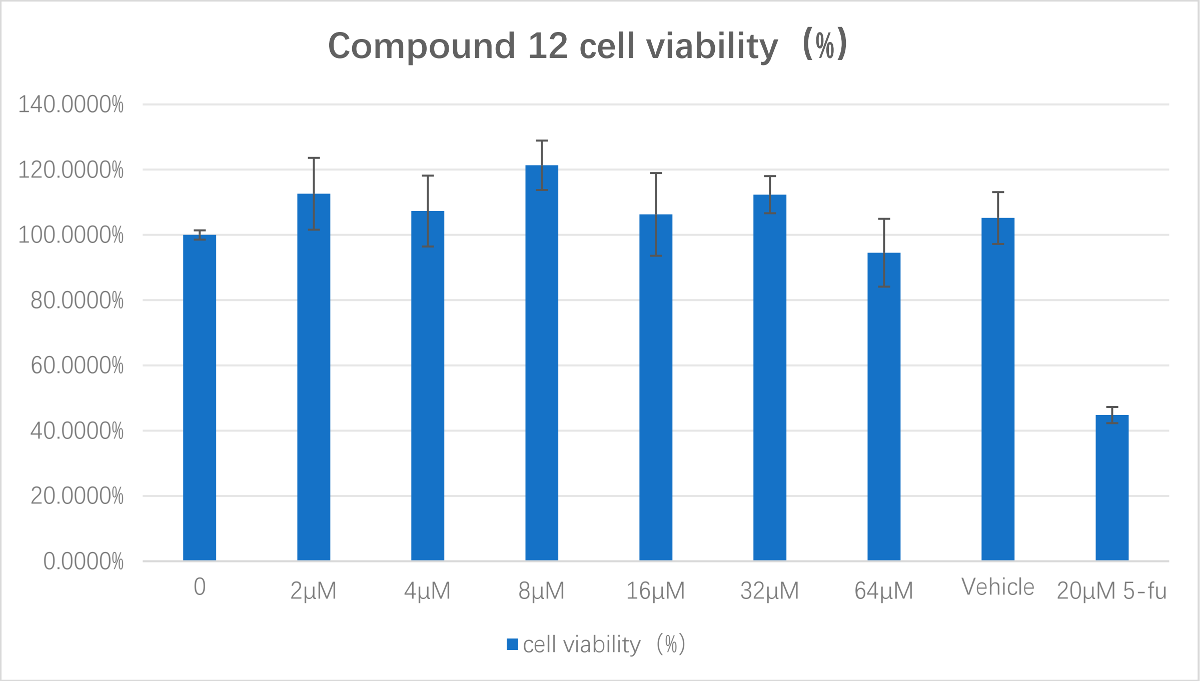

Figure S14. Determination of cellular activity of Compound 13

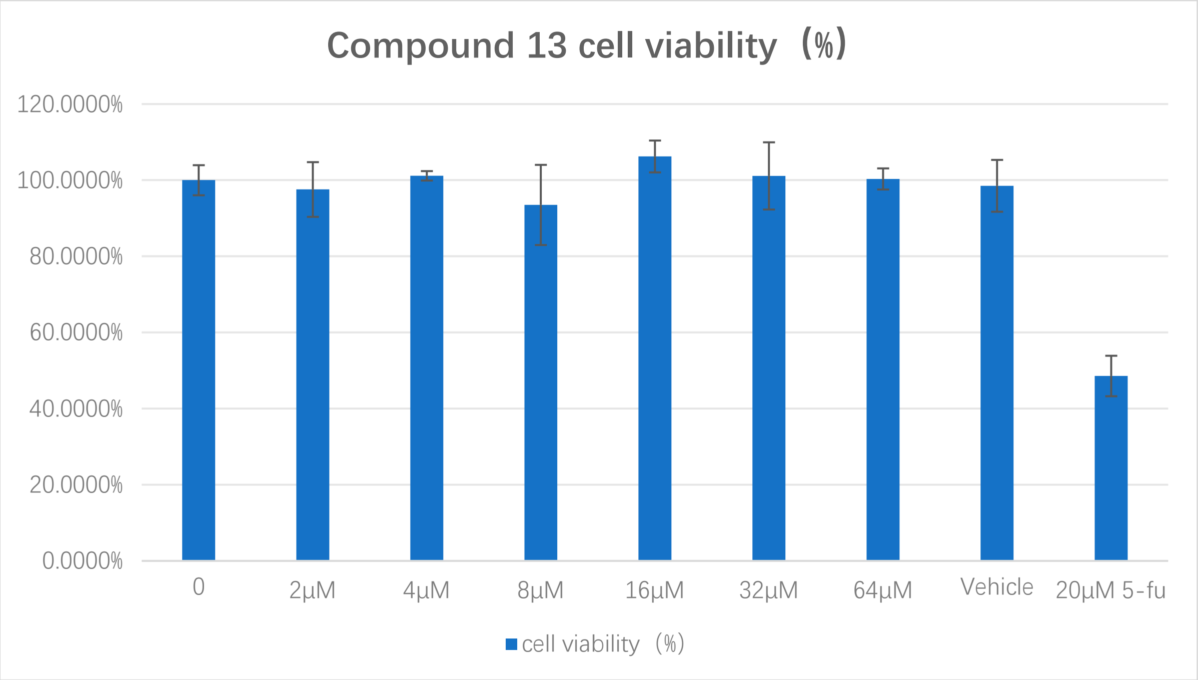

Figure S15. Determination of cellular activity of Compound 14

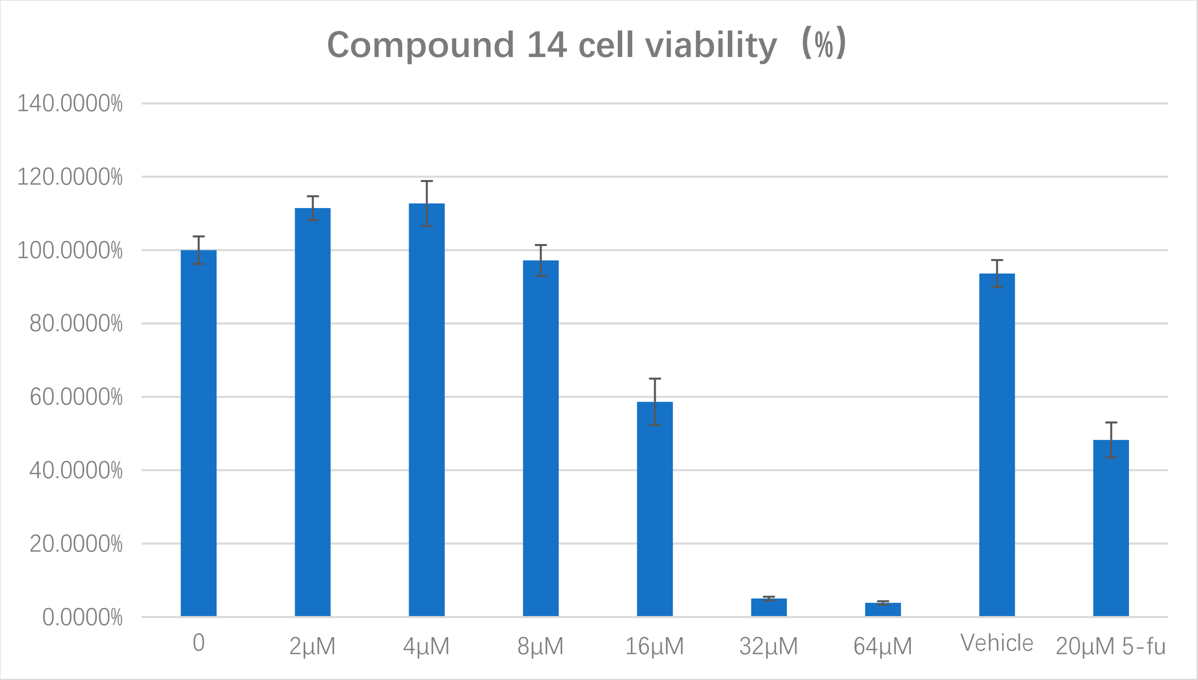

Figure S16. Determination of cellular activity of Compound 15

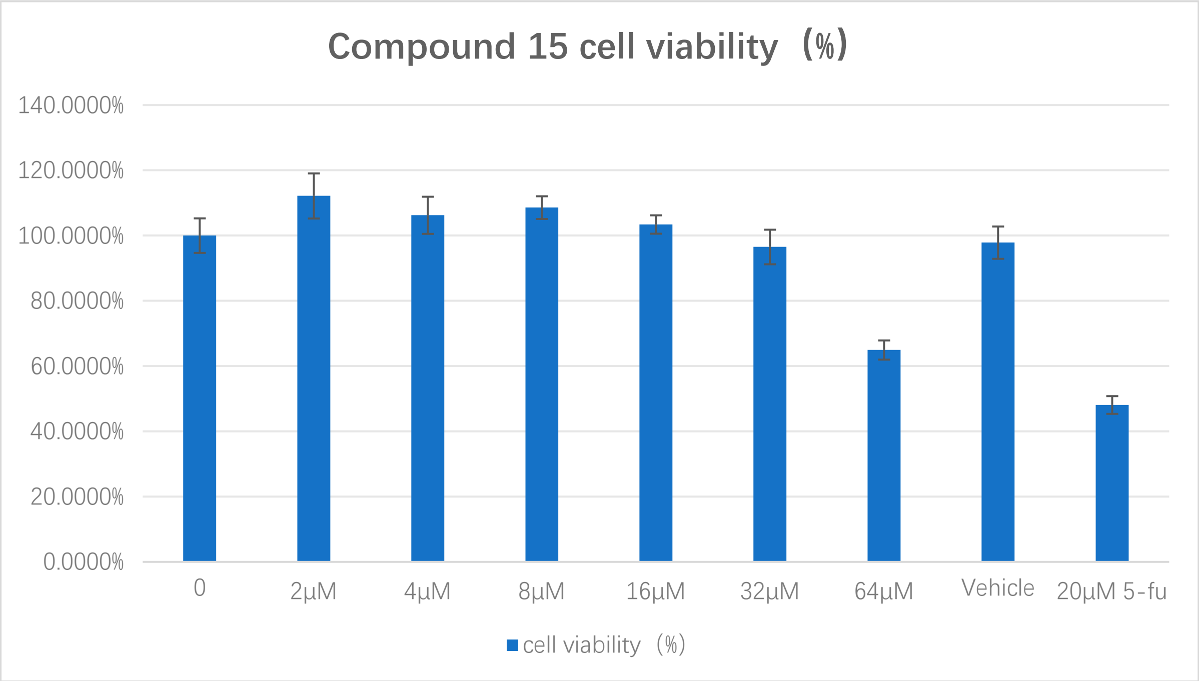

Figure S17. Determination of cellular activity of Compound 16

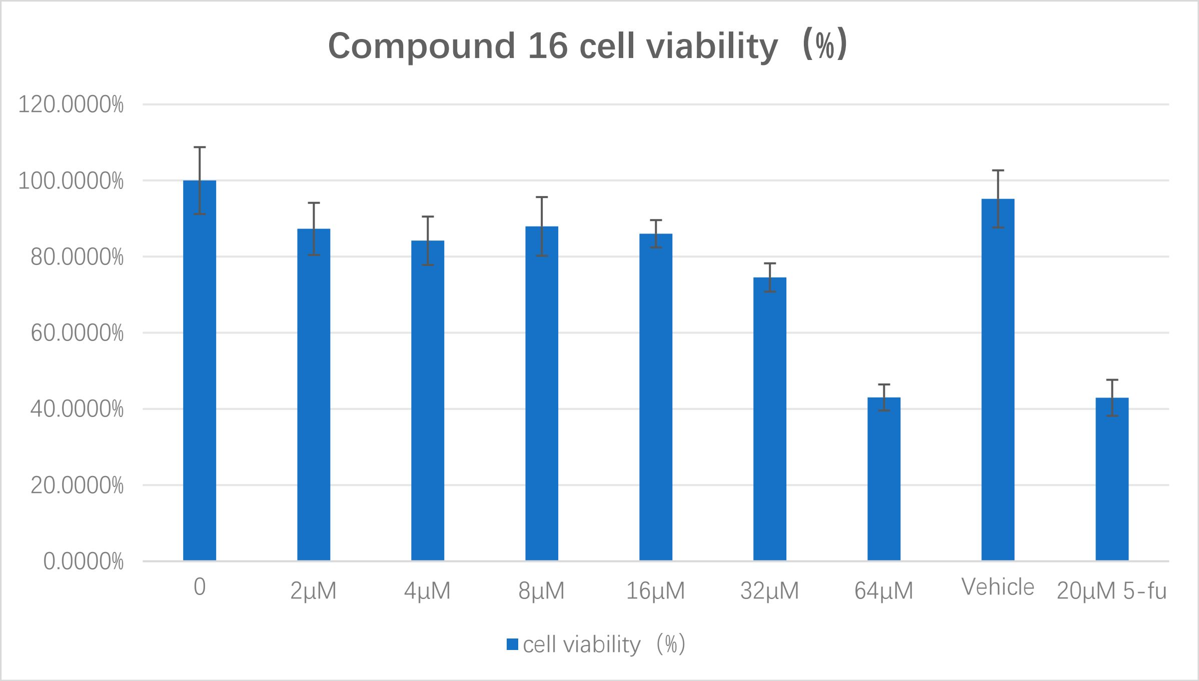

Figure S18. Determination of cellular activity of Compound 17

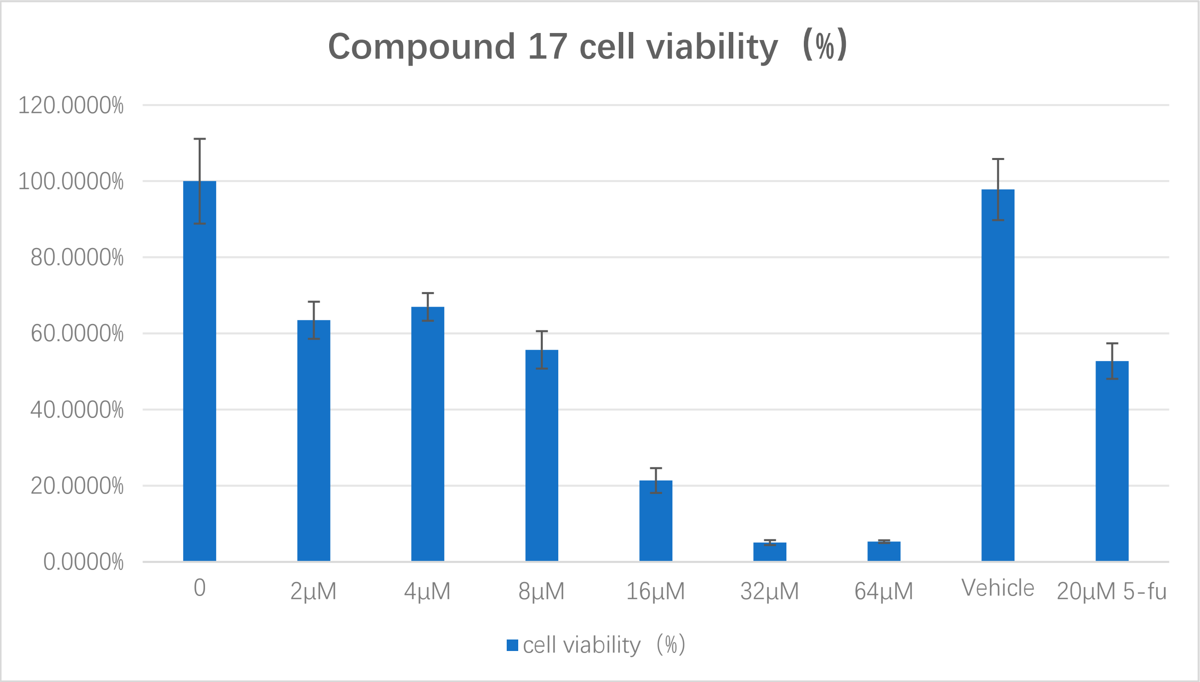

Figure S19. Determination of cellular activity of Compound 18

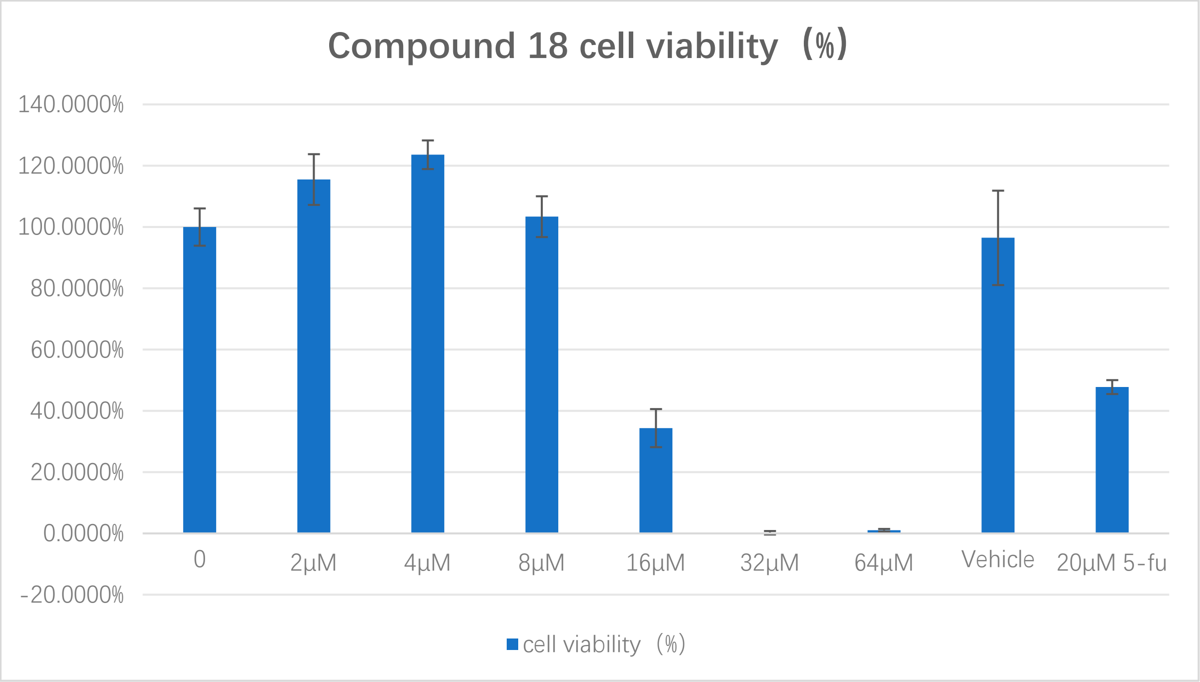

Figure S20. Determination of cellular activity of Compound 19

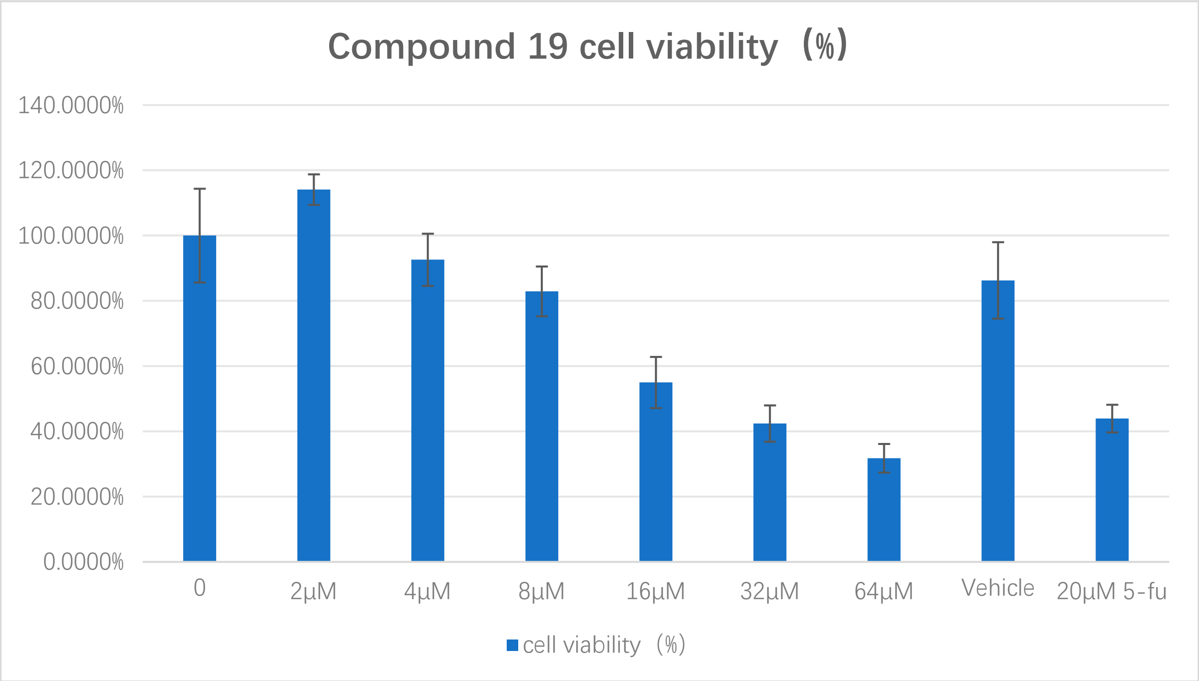

Figure S21. Determination of cellular activity of Compound 20

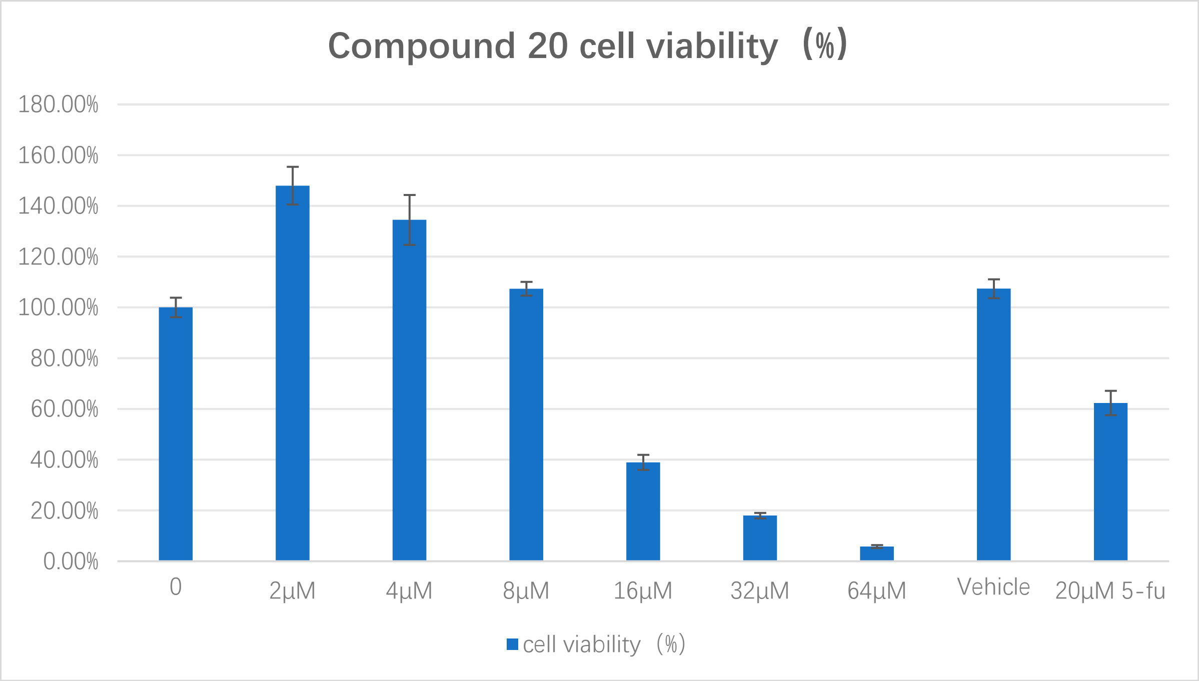

Figure S22. Determination of cellular activity of Compound 21

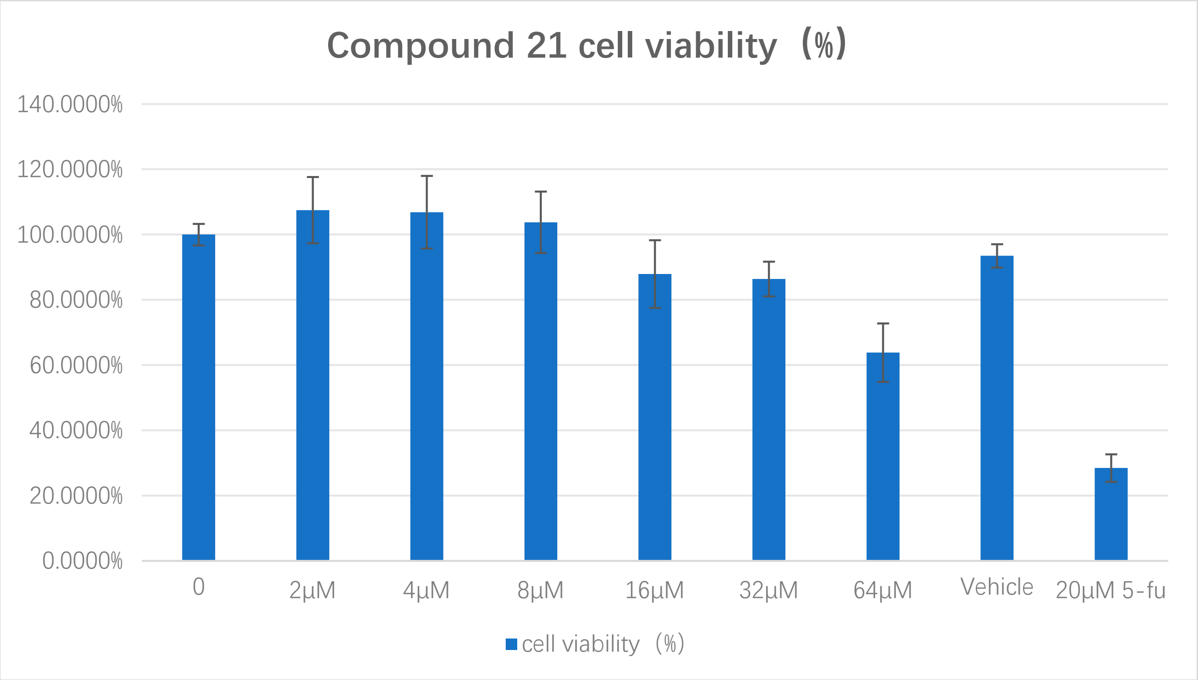

Figure S23. Determination of cellular activity of Compound 22

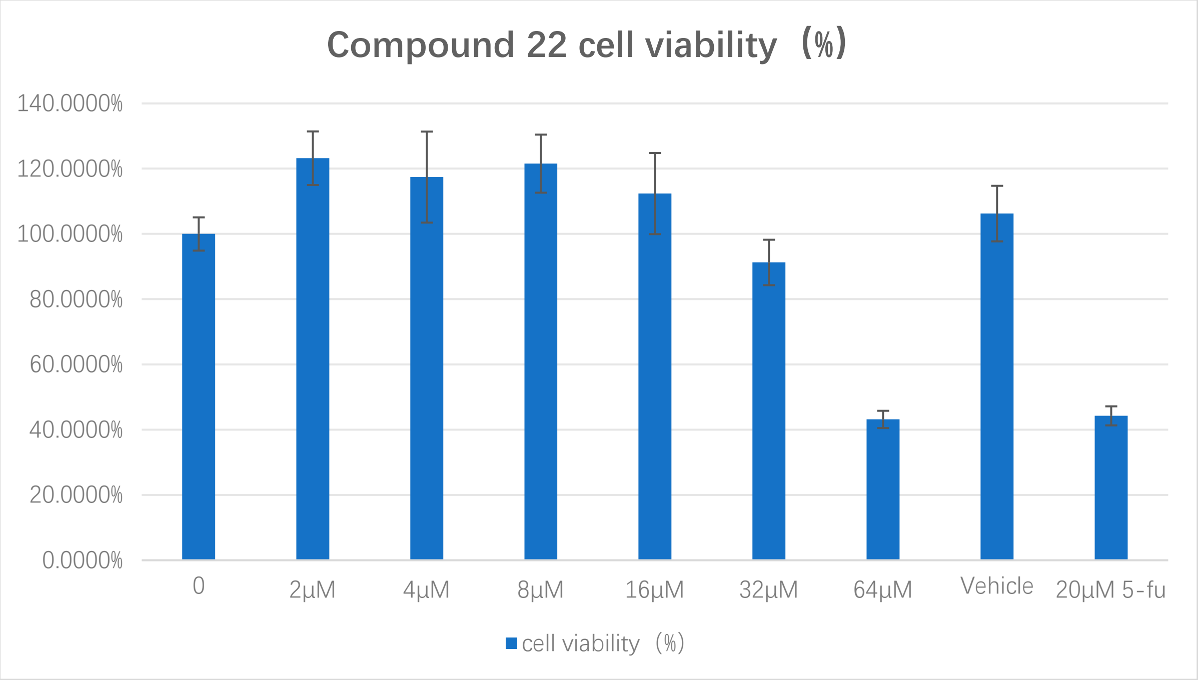

Figure S24. Determination of cellular activity of Compound 23

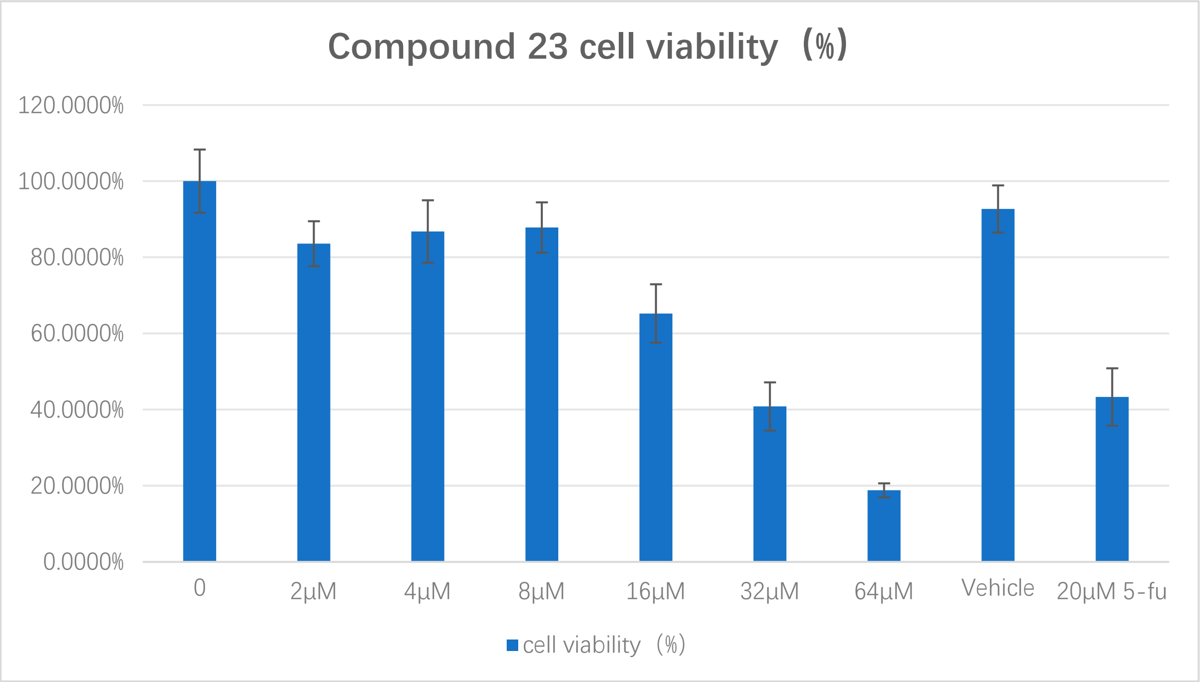

4. Synthesis of procedure

*(3S,8R,10R,12R,14R)-12-hydroxy-4,4,8,10,14-pentamethyl-17-((R)-2,6,6-trimethyltetrahydro-2H-pyran-2-yl)hexadecahydro-1H-cyclopenta[a]phenanthren-3-yl acetate (**1**)*

PD (2.5 g, 5.426 mmol) and acetic acid (0.47 mL) were introduced. The resulting crude product was purified via silica gel column chromatography with (petroleum ether/ethyl acetate 5:1) as the eluent, furnishing compound (3*S*,8*R*,10*R*,12*R*,14*R*)-12-hydroxy-4,4,8,10,14-pentamethyl-17-((*R*)-2,6,6-trimethyltetrahydro-2H-pyran-2-yl)hexadecahydro-1H-cyclopenta[a]phenanthren-3-yl acetate (1) as a white amorphous powder (2.619 g, 96% yield). $[\alpha]_D^{25}$ = + 31.13 (c 1.07, $CHCl_3$). ^{1}H NMR (500 MHz, Chloroform-*d*) δ 6.25 (s, 1H), 4.47 (dd, *J* = 10.5, 5.9 Hz, 1H), 3.53 (td, *J* = 10.4, 5.2 Hz, 1H), 2.04 (s, 3H), 1.96 – 1.86 (m, 2H), 1.81 – 1.72 (m, 3H), 1.66 – 1.62 (m, 2H), 1.58 – 1.34 (m, 9H), 1.29 – 1.16 (m, 15H), 1.09 – 1.01 (m, 2H), 0.97 (s, 3H), 0.89 (d, *J* = 13.8 Hz, 6H), 0.85 (s, 6H). ^{13}C NMR (126 MHz, $CDCl_3$) δ 171.12, 81.04, 76.81, 73.24, 70.02, 56.13, 54.88, 51.35, 50.01, 49.33, 39.98, 38.75, 38.02, 37.21, 36.61, 35.91, 34.98, 33.17, 31.28, 30.72, 28.14, 27.30, 25.32, 23.90, 21.46, 19.58, 18.36, 17.19, 16.62, 16.43, 16.33, 15.79. HRMS (ESI, m/z): calculated for $[M + H]^+$ $C_{32}H_{55}O_4$ 505.6654, found: 505.6626.

*(3S,8R,10R,12R,14R)-12-hydroxy-4,4,8,10,14-pentamethyl-17-((R)-2,6,6-trimethyltetrahydro-2H-pyran-2-yl)hexadecahydro-1H-cyclopenta[a]phenanthren-3-yl (tert-butoxycarbonyl)-L-phenylalaninate (**2**)*

PD (0.1 g, 0.217 mmol) and Boc-L-phenylalanine (57.58 mg, 0.217 mmol) were added. The crude product was purified by silica gel column chromatography eluted with (petroleum ether/ethyl acetate 5:1), affording compound (3*S*,8*R*,10*R*,12*R*,14*R*)-12-hydroxy-4,4,8,10,14-pentamethyl-17-((*R*)-2,6,6-trimethyltetrahydro-2H-pyran-2-yl)hexadecahydro-1H-cyclopenta[a]phenanthren-3-yl (tert-butoxycarbonyl)-L-phenylalaninate (2) as a white amorphous powder (96.80 mg, 63% yield). $[\alpha]_D^{25}$ = + 2.70 (c 0.74, $CHCl_3$). ^{1}H NMR (500 MHz, Chloroform-*d*) δ 7.28 (m, 2H), 7.25 – 7.21 (m, 1H), 7.17 (d, *J* = 7.3 Hz, 2H), 6.25 (s, 1H), 4.88 (d, *J* = 8.4 Hz, 1H), 4.61 – 4.45 (m, 2H), 3.53 (m, 1H), 3.17 – 2.99 (m, 2H), 1.94 – 1.86 (m, 2H), 1.76 (m, 3H), 1.67 – 1.58 (m, 5H), 1.50 (m, 5H), 1.40 (m, 13H), 1.26 (m, 5H), 1.22 (s, 3H), 1.18 (s, 3H), 1.04 (m, 2H), 0.97 (s, 3H), 0.88 (d, *J* = 6.8 Hz, 6H), 0.78 (d, *J* = 9.0 Hz, 6H). ^{13}C NMR (126 MHz, $CDCl_3$) δ 171.97, 155.21, 136.30, 129.53, 128.67, 127.07, 82.61, 79.89, 76.80, 73.25, 70.00, 60.52, 56.12, 54.87, 54.79, 51.34, 49.98, 49.32, 39.96, 38.67, 38.03, 37.16, 36.60, 35.90, 34.95, 33.17, 31.27, 30.71, 28.46, 28.15, 27.30, 25.31, 23.75, 21.18, 19.57, 18.29, 17.18, 16.62, 16.43, 16.27, 15.78, 14.34. HRMS (ESI, m/z): calculated for $[M + H]^+$ $C_{44}H_{70}NO_6$ 709.7591, found: 709.7532.

*(3S,8R,10R,12R,14R)-12-hydroxy-4,4,8,10,14-pentamethyl-17-((R)-2,6,6-trimethyltetrahydro-2H-pyran-2-yl)hexadecahydro-1H-cyclopenta[a]phenanthren-3-yl (tert-butoxycarbonyl)-L-valinate (**3**)*

PD (0.1 g, 0.217 mmol) and N-Boc-L-valine (47 mg, 0.217 mmol) were combined. The crude product was purified by silica gel column chromatography using (petroleum ether/ethyl acetate 5:1) as eluent to furnish compound (3*S*,8*R*,10*R*,12*R*,14*R*)-12-hydroxy-4,4,8,10,14-pentamethyl-17-((*R*)-2,6,6-trimethyltetrahydro-2H-pyran-2-yl)hexadecahydro-1H-cyclopenta[a]phenanthren-3-yl (tert-butoxycarbonyl)-L-valinate (3) as a white amorphous powder (95.96 mg, 67% yield). $[\alpha]_D^{25}$ = + 4.82 (c 0.75, $CHCl_3$). ^{1}H NMR (500 MHz, Chloroform-*d*) δ 6.25 (d, *J* = 6.0 Hz, 1H), 4.99 (d, *J* = 9.1 Hz, 1H), 4.51

(dd, *J* = 10.8, 5.4 Hz, 1H), 4.21 (dd, *J* = 8.9, 3.9 Hz, 1H), 3.53 (mf, 1H), 2.17 (m, 1H), 1.95 – 1.85 (m, 2H), 1.81 – 1.72 (m, 3H), 1.70 – 1.55 (m, 7H), 1.55 – 1.46 (m, 5H), 1.44 (s, 9H), 1.41 – 1.33 (m, 2H), 1.25 (m, 6H), 1.21 (s, 3H), 1.18 (s, 3H), 1.05 – 0.94 (m, 8H), 0.90 (s, 3H), 0.88 – 0.82 (m, 12H). ^{13}C NMR (126 MHz, $CDCl_3$) δ 172.20, 155.90, 82.29, 79.74, 76.80, 73.25, 70.00, 60.52, 59.05, 56.12, 54.87, 51.34, 49.99, 49.32, 39.96, 38.67, 38.03, 37.17, 36.60, 35.90, 34.96, 33.16, 31.36, 31.27, 30.71, 28.50, 28.21, 27.29, 25.31, 23.96, 19.57, 18.33, 17.35, 17.19, 16.77, 16.42, 16.28, 15.79, 14.34. HRMS (ESI, m/z): calculated for $[M + H]^+$ $C_{40}H_{70}NO_6$ 660.9671, found: 660.9657.

*(3S,8R,10R,12R,14R)-12-hydroxy-4,4,8,10,14-pentamethyl-17-((R)-2,6,6-trimethyltetrahydro-2H-pyran-2-yl)hexadecahydro-1H-cyclopenta[a]phenanthren-3-yl (tert-butoxycarbonyl)-L-methioninate (**4**)*

PD (0.1 g, 0.217 mmol) and N-Boc-L-methionine (65 mg, 0.2604 mmol) were added. The resulting crude product was purified via silica gel column chromatography eluted with (petroleum ether/ethyl acetate 5:1), affording compound (3*S*,8*R*,10*R*,12*R*,14*R*)-12-hydroxy-4,4,8,10,14-pentamethyl-17-((*R*)-2,6,6-trimethyltetrahydro-2H-pyran-2-yl)hexadecahydro-1H-cyclopenta[a]phenanthren-3-yl (tert-butoxycarbonyl)-L-methioninate (4) as a white amorphous powder (105.12 mg, 70% yield). $[\alpha]_D^{25}$ = + 43.23 (c 0.62, $CHCl_3$). ^{1}H NMR (500 MHz, Chloroform-*d*) δ 6.24 (s, 1H), 5.11 (d, *J* = 8.0 Hz, 1H), 4.60 – 4.47 (m, 1H), 4.44 – 4.35 (m, 1H), 3.53 (m, 1H), 2.53 (m, 2H), 2.09 (s, 5H), 1.94 – 1.85 (m, 3H), 1.81 – 1.73 (m, 3H), 1.67 – 1.56 (m, 6H), 1.52 (m, 5H), 1.44 (s, 9H), 1.39 – 1.34 (m, 1H), 1.26 (m, 7H), 1.21 (s, 3H), 1.18 (s, 3H), 1.07 – 1.00 (m, 2H), 0.98 (s, 3H), 0.90 (s, 3H), 0.87 (d, *J* = 4.1 Hz, 6H), 0.85 (s, 3H). ^{13}C NMR (126 MHz, $CDCl_3$) δ 172.13, 155.53, 82.60, 82.54, 76.80, 73.25, 69.99, 56.09, 54.86, 51.34, 49.98, 49.31, 39.96, 38.65, 38.11, 37.18, 36.59, 35.89, 34.93, 34.10, 33.16, 32.70, 31.27, 30.71, 30.16, 28.48, 28.28, 27.29, 25.77, 25.30, 23.87, 19.56, 18.31, 17.19, 16.74, 16.42, 16.28, 15.78, 15.61. HRMS (ESI, m/z): calculated for $[M + H]^+$ $C_{40}H_{70}NO_6S$ 695.4681, found: 695.4624.

*(3S,8R,10R,12R,14R)-12-hydroxy-4,4,8,10,14-pentamethyl-17-((R)-2,6,6-trimethyltetrahydro-2H-pyran-2-yl)hexadecahydro-1H-cyclopenta[a]phenanthren-3-yl (tert-butoxycarbonyl)glycinate (**5**)*

PD (100 mg, 0.217 mmol) and Boc-glycine (46 mg, 0.2604 mmol) were added. The resulting crude product was purified by silica gel column chromatography eluted with (petroleum ether/ethyl acetate 2:1), affording compound (3*S*,8*R*,10*R*,12*R*,14*R*)-12-hydroxy-4,4,8,10,14-pentamethyl-17-((*R*)-2,6,6-trimethyltetrahydro-2H-pyran-2-yl)hexadecahydro-1H-cyclopenta[a]phenanthren-3-yl (tert-butoxycarbonyl)glycinate (5) as a white amorphous powder (112.63 mg, 84% yield). $[\alpha]_D^{25}$ = + 42 (c 0.67, $CHCl_3$). ^{1}H NMR (500 MHz, Chloroform-*d*) δ 6.24 (s, 1H), 4.99 (s, 1H), 4.64 – 4.47 (m, 1H), 3.89 (d, *J* = 5.1 Hz, 2H), 3.53 m, 1H), 1.96 – 1.85 (m, 2H), 1.77 (m, 3H), 1.68 – 1.59 (m, 5H), 1.51 (m, 4H), 1.45 (s, 11H), 1.26 (q, *J* = 7.2, 5.6 Hz, 8H), 1.21 (s, 3H), 1.18 (s, 3H), 1.07 – 0.99 (m, 2H), 0.97 (s, 3H), 0.90 (s, 3H), 0.87 (s, 3H), 0.85 (s, 6H). ^{13}C NMR (126 MHz, $CDCl_3$) δ 170.25, 155.76, 82.39, 80.00, 73.25, 70.00, 60.53, 56.10, 54.88, 51.35, 50.00, 49.33, 42.81, 39.97, 38.69, 38.13, 37.19, 36.61, 35.91, 34.95, 33.17, 31.28, 30.72, 28.48, 28.17, 27.30, 25.31, 23.86, 19.57, 18.33, 17.19, 16.59, 16.43, 16.32, 15.79, 14.35. HRMS (ESI, m/z): calculated for $[M + H]^+$ $C_{37}H_{64}NO_6$ 618.7533, found: 618.7591.

*1-(tert-butyl) 2-((3S,8R,10R,12R,14R)-12-hydroxy-4,4,8,10,14-pentamethyl-17-((R)-2,6,6-trimethyltetrahydro-2H-pyran-2-yl)hexadecahydro-1H-cyclopenta[a]phenanthren-3-yl) pyrrolidine-1,2-dicarboxylate (**6**)*

PD (0.1 g, 0.217 mmol) and Boc-L-proline (56 mg, 0.2624 mmol) were added. The resulting crude product was purified by silica gel column chromatography eluted with (petroleum ether/ethyl acetate 2:1), affording 1-(tert-butyl) 2-((3*S*,8*R*,10*R*,12*R*,14*R*)-12-hydroxy-4,4,8,10,14-pentamethyl-17-((*R*)-2,6,6-trimethyltetrahydro-2H-pyran-2-yl)hexadecahydro-1H-cyclopenta[a]phenanthren-3-yl) pyrrolidine-1,2-dicarboxylate (6) as a white amorphous powder (91.38 mg, 64% yield). $[\alpha]_D^{25}$ = - 30 (c 0.52, $CHCl_3$). ^{1}H NMR (500 MHz, Chloroform-*d*) δ 6.25 (d, *J* = 5.3 Hz, 1H), 4.58 – 4.44 (m, 1H), 4.28 (dd, *J* = 40.9, 7.0 Hz, 1H), 3.57 – 3.35 (m, 3H), 2.26 – 2.15 (m, 1H), 2.01 – 1.85 (m, 5H), 1.76 (m, 3H), 1.65 (m, 3H), 1.52 (m, 6H), 1.45 (s, 3H), 1.42 (s, 6H), 1.39 – 1.28 (m, 6H), 1.22 (s, 3H), 1.18 (s, 3H), 1.08 – 1.00 (m, 3H), 0.97 (s, 3H), 0.90 (s, 3H), 0.88 – 0.83 (m, 12H). ^{13}C NMR (126 MHz, $CDCl_3$) δ 172.94, 130.46, 81.44, 80.49, 80.06, 79.68, 73.26, 70.01, 59.59, 56.09, 54.86, 51.35, 49.30, 46.40, 39.96, 38.64, 38.15, 37.17, 36.59, 35.89, 34.93, 33.18, 31.28, 30.71, 29.85, 28.65, 28.56, 28.30, 27.29, 25.31, 24.43, 23.61, 19.57, 18.31, 17.18, 16.66, 16.43, 16.30, 15.77, 14.27. HRMS (ESI, m/z): calculated for $[M + H]^+$ $C_{40}H_{68}NO_6$ 659.1257, found: 659.1238.

*(3S,8R,10R,12R,14R)-12-hydroxy-4,4,8,10,14-pentamethyl-17-((R)-2,6,6-trimethyltetrahydro-2H-pyran-2-yl)hexadecahydro-1H-cyclopenta[a]phenanthren-3-yl 2-((tert-butoxycarbonyl)amino)-3-(indolin-3-yl)propanoate (**7**)*

PD (0.1 g, 0.217 mmol) and N-(tert-butoxycarbonyl)-L-tryptophan (79 mg, 0.2604 mmol) were added. The resulting crude product was purified by silica gel column chromatography eluted with (petroleum ether/ethyl acetate 2:1), affording compound (3*S*,8*R*,10*R*,12*R*,14*R*)-12-hydroxy-4,4,8,10,14-pentamethyl-17-((*R*)-2,6,6-trimethyltetrahydro-2H-pyran-2-yl)hexadecahydro-1H-cyclopenta[a]phenanthren-3-yl 2-((tert-butoxycarbonyl)amino)-3-(1H-indol-3-yl)propanoate (7) as a white amorphous powder (118.66 mg, 73% yield). $[\alpha]_D^{25}$ = + 28.47 (c 0.79, $CHCl_3$). ^{1}H NMR (500 MHz, Chloroform-*d*) δ 8.11 (d, *J* = 10.6 Hz, 1H), 7.61 (d, *J* = 7.7 Hz, 1H), 7.36 (t, *J* = 8.0 Hz, 1H), 7.20 (q, *J* = 6.8 Hz, 1H), 7.12 (t, *J* = 7.3 Hz, 1H), 7.05 (d, *J* = 8.7 Hz, 1H), 6.27 (s, 1H), 5.10 – 4.95 (m, 1H), 4.75 – 4.61 (m, 1H), 4.48 (dd, *J* = 10.2, 4.5 Hz, 1H), 3.59 – 3.49 (m, 1H), 3.27 (m, 2H), 1.96 – 1.67 (m, 8H), 1.59 – 1.36 (m, 19H), 1.26 (s, 9H), 1.20 (d, *J* = 20.5 Hz, 6H), 1.10 – 0.93 (m, 5H), 0.83 (m, 7H), 0.75 (d, *J* = 6.5 Hz, 3H). ^{13}C NMR (126 MHz, $CDCl_3$) δ 183.31, 122.76, 122.35, 119.77, 119.13, 118.96, 111.37, 110.69, 82.34, 80.39, 80.06, 78.03, 73.26, 70.02, 56.09, 54.86, 51.34, 49.96, 49.29, 39.94, 38.66, 38.02, 37.13, 36.59, 35.89, 34.93, 33.17, 30.69, 29.85, 29.57, 28.48, 28.40, 28.02, 27.29, 26.20, 26.04, 25.30, 24.69, 23.69, 21.20, 19.56, 18.28, 17.18, 16.60, 16.26, 15.76. HRMS (ESI, m/z): calculated for $[M + H]^+$ $C_{46}H_{73}N_2O_6$ 749.9907, found: 749.9913.

*1-(tert-butyl) 4-((3S,8R,10R,12R,14R)-12-hydroxy-4,4,8,10,14-pentamethyl-17-((R)-2,6,6-trimethyltetrahydro-2H-pyran-2-yl)hexadecahydro-1H-cyclopenta[a]phenanthren-3-yl) (tert-butoxycarbonyl)aspartate (**8**)*

PD (0.1 g, 0.217 mmol) and tert-butyl N-(tert-butoxycarbonyl)-L-aspartate (94 mg, 0.3255 mmol) were added. The resulting crude product was purified by silica gel column chromatography eluted with (petroleum ether/ethyl acetate 3:1), affording 1-(tert-butyl) 4-((3*S*,8*R*,10*R*,12*R*,14*R*)-12-hydroxy-4,4,8,10,14-pentamethyl-17-((*R*)-2,6,6-trimethyltetrahydro-2H-pyran-2-yl)hexadecahydro-1H-cyclopenta[a]phenanthren-3-yl) (tert-butoxycarbonyl)-L-aspartate (8) as a white amorphous powder (88.96 mg, 56% yield). $[\alpha]_D^{25}$ = + 16.13 (c 0.62, $CHCl_3$). ^{1}H NMR (500 MHz, Chloroform-*d*) δ 6.26 (s, 1H), 5.46 (d, *J* = 8.5 Hz, 1H), 4.52 – 4.39 (m, 2H), 4.06 (d, *J* = 6.9 Hz, 1H), 3.56 – 3.45 (m, 2H), 2.91 – 2.78 (m, 2H), 2.04 – 1.98 (m, 1H), 1.96 – 1.87 (m, 4H), 1.81 – 1.73 (m, 4H), 1.67 – 1.62 (m, 6H), 1.46 (s, 9H), 1.43 (s, 9H), 1.38 – 1.33 (m, 4H), 1.26 (s, 3H), 1.25 (s, 3H), 1.22 (s, 3H), 1.18 (s, 3H), 0.97 (s, 3H), 0.90 (s, 3H), 0.87

(s, 3H), 0.84 (d, *J* = 4.7 Hz, 6H). ^{13}C NMR (126 MHz, $CDCl_3$) δ 190.50, 187.61, 139.30, 82.37, 81.83, 76.80, 73.25, 70.02, 56.12, 54.86, 51.34, 49.98, 49.30, 39.95, 38.70, 37.99, 37.17, 36.59, 35.89, 34.93, 34.10, 33.17, 31.28, 30.70, 29.85, 28.47, 28.18, 28.09, 27.29, 25.76, 25.31, 25.09, 23.90, 22.84, 19.57, 18.34, 17.18, 16.65, 16.43, 16.30, 15.77. HRMS (ESI, m/z): calculated for $[M + H]^+$ $C_{43}H_{74}NO_8$ 733.6587, found: 733.6558.

*(3S,8R,10R,12R,14R)-12-hydroxy-4,4,8,10,14-pentamethyl-17-((R)-2,6,6-trimethyltetrahydro-2H-pyran-2-yl)hexadecahydro-1H-cyclopenta[a]phenanthren-3-yl N2,N6-bis(tert-butoxycarbonyl)-L-lysinate (**9**)*

PD (0.1 g, 0.217 mmol) and (S)-2,6-bis(tert-butoxycarbonylamino)hexanoic acid (112 mg, 0.3255 mmol) were added. The resulting crude product was purified by silica gel column chromatography eluted with (petroleum ether/ethyl acetate 2:1), affording compound (3*S*,8*R*,10*R*,12*R*,14*R*)-12-hydroxy-4,4,8,10,14-pentamethyl-17-((*R*)-2,6,6-trimethyltetrahydro-2H-pyran-2-yl)hexadecahydro-1H-cyclopenta[a]phenanthren-3-yl N2,N6-bis(tert-butoxycarbonyl)-L-lysinate (9) as a white amorphous powder (90.76 mg, 53% yield). $[\alpha]_D^{25}$ = + 32.84 (c 0.63, $CHCl_3$). ^{1}H NMR (500 MHz, Chloroform-*d*) δ 6.25 (s, 1H), 5.17 – 4.95 (m, 1H), 4.62 – 4.41 (m, 2H), 4.36 – 4.18 (m, 1H), 3.53 (m, 1H), 3.15 – 3.05 (m, 2H), 1.96 – 1.86 (m, 2H), 1.78 (m, 4H), 1.64 (m, 6H), 1.50 (m, 4H), 1.44 (m, 22H), 1.38 – 1.33 (m, 2H), 1.26 (m, 5H), 1.22 (s, 3H), 1.18 (s, 3H), 1.05 (m, 2H), 0.98 (s, 3H), 0.90 (s, 3H), 0.88 – 0.84 (m, 9H). ^{13}C NMR (126 MHz, $CDCl_3$) δ 156.17, 148.96, 82.29, 76.50, 73.25, 70.00, 56.09, 54.87, 53.78, 51.34, 49.97, 49.30, 39.95, 38.65, 38.10, 37.17, 36.59, 35.89, 34.93, 33.17, 32.70, 31.27, 30.71, 29.85, 29.77, 29.75, 28.58, 28.52, 28.20, 27.28, 25.31, 23.89, 22.61, 19.56, 18.31, 17.18, 16.74, 16.43, 16.29, 15.78. HRMS (ESI, m/z): calculated for $[M + H]^+$ $C_{46}H_{81}N_2O_8S$ 790.0967, found: 790.0954.

*(3S,8R,10R,12R,14R)-3-acetoxy-4,4,8,10,14-pentamethyl-17-((R)-2,6,6-trimethyltetrahydro-2H-pyran-2-yl)hexadecahydro-1H-cyclopenta[a]phenanthren-12-yl 2-chloroacetate (**10**)*

The crude product was purified by silica gel column chromatography eluted with (petroleum ether/ethyl acetate 5:1), affording compound (3*S*,8*R*,10*R*,12*R*,14*R*)-3-acetoxy-4,4,8,10,14-pentamethyl-17-((*R*)-2,6,6-trimethyltetrahydro-2H-pyran-2-yl)hexadecahydro-1H-cyclopenta[a]phenanthren-12-yl 2-chloroacetate (10) as a white amorphous powder (2.09 g, 90% yield). $[\alpha]_D^{25}$ = + 17.32 (c 0.99, $CHCl_3$). ^{1}H NMR (500 MHz, Chloroform-*d*) δ 5.04 (td, J = 10.5, 6.0 Hz, 1H), 4.49 (dd, *J* = 11.4, 4.2 Hz, 1H), 4.14 – 4.01 (m, 2H), 2.18 (q, *J* = 9.3 Hz, 1H), 2.04 (s, 3H), 1.96 – 1.82 (m, 2H), 1.79 – 1.70 (m, 1H), 1.69 – 1.60 (m, 4H), 1.54 – 1.40 (m, 7H), 1.36 – 1.21 (m, 9H), 1.15 – 1.06 (m, 9H), 0.97 (s, 3H), 0.93 (s, 3H), 0.87 (s, 3H), 0.85 (s, 6H). ^{13}C NMR (126 MHz, $CDCl_3$) δ 171.05, 166.68, 80.75, 76.49, 75.57, 71.17, 55.91, 51.78, 51.11, 49.55, 45.36, 42.11, 39.70, 38.54, 38.02, 37.40, 37.13, 34.32, 31.77, 30.48, 30.23, 29.85, 28.30, 28.15, 26.36, 23.87, 23.72, 21.44, 18.25, 18.17, 16.67, 16.59, 16.00, 15.95. HRMS (ESI, m/z): calculated for $[M + H]^+$ $C_{34}H_{56}ClO_5$ 580.2677, found: 580.2625.

*(3S,8R,10R,12R,14R)-3-acetoxy-4,4,8,10,14-pentamethyl-17-((R)-2,6,6-trimethyltetrahydro-2H-pyran-2-yl)hexadecahydro-1H-cyclopenta[a]phenanthren-12-yl 2-(4-(pyridin-3-yl)piperazin-1-yl)acetate (**11**)*

Compound 10 (100 mg, 0.17 mmol) and 1-(4-pyridyl)piperazine (42.4 mg, 0.26 mmol) were combined. The resulting crude product was purified by silica gel column chromatography eluted with (dichloromethane/methanol 10:1), affording compound (3*S*,8*R*,10*R*,12*R*,14*R*)-3-acetoxy-4,4,8,10,14-pentamethyl-17-((*R*)-2,6,6-trimethyltetrahydro-2H-pyran-2-yl)hexadecahydro-1H-

cyclopenta[a]phenanthren-12-yl 2-(4-(pyridin-3-yl)piperazin-1-yl)acetate (11) as a white amorphous powder (63.50 mg, 53% yield). $[\alpha]_D^{25}$ = + 28.82 (c 0.68, $CHCl_3$). ^{1}H NMR (500 MHz, Chloroform-*d*) δ 8.24 (d, *J* = 6.4 Hz, 2H), 6.68 (d, *J* = 6.6 Hz, 2H), 4.98 (m, 1H), 4.53 – 4.44 (m, 1H), 3.48 – 3.37 (m, 4H), 3.28 – 3.18 (m, 2H), 2.72 (m, 4H), 2.08 – 2.05 (m, 1H), 2.04 (s, 3H), 1.96 – 1.83 (m, 4H), 1.66 – 1.60 (m, 4H), 1.52 – 1.45 (m, 6H), 1.35– 1.32 (m, 2H), 1.17 (d, *J* = 2.2 Hz, 6H), 1.13 (s, 3H), 1.03 – 0.98 (m, 2H), 0.97 (s, 3H), 0.93 (s, 3H), 0.88 – 0.83 (m, 14H). ^{13}C NMR (126 MHz, $CDCl_3$) δ 222.88, 198.60, 183.46, 158.36, 127.04, 115.02, 108.25, 80.74, 75.81, 75.70, 75.37, 72.62, 71.10, 59.73, 58.90, 52.50, 52.24, 51.65, 51.62, 50.95, 49.77, 46.05, 39.71, 38.61, 38.58, 38.02, 37.12, 34.38, 29.85, 29.81, 29.51, 28.46, 28.13, 22.84, 22.22, 21.45, 18.39, 16.64, 16.57, 16.12, 15.85. HRMS (ESI, m/z): calculated for $[M + H]^+$ $C_{43}H_{68}N_3O_5$ 709.0584, found: 709.0544.

*(3S,8R,10R,12R,14R)-3-acetoxy-4,4,8,10,14-pentamethyl-17-((R)-2,6,6-trimethyltetrahydro-2H-pyran-2-yl)hexadecahydro-1H-cyclopenta[a]phenanthren-12-yl 2-(4-phenylpiperazin-1-yl)acetate (**12**)*

Compound 10 (100 mg, 0.17 mmol) and 1-phenylpiperazine (42 mg, 0.259 mmol) were added. The crude product was purified by silica gel column chromatography eluted with (petroleum ether/ethyl acetate 5:1), affording (3*S*,8*R*,10*R*,12*R*,14*R*)-3-acetoxy-4,4,8,10,14-pentamethyl-17-((*R*)-2,6,6-trimethyltetrahydro-2H-pyran-2-yl)hexadecahydro-1H-cyclopenta[a]phenanthren-12-yl 2-(4-phenylpiperazin-1-yl)acetate (12) as a white amorphous powder(113.86 mg, 95% yield). $[\alpha]_D^{25}$ = + 26.72 (c 0.91, $CHCl_3$). ^{1}H NMR (500 MHz, Chloroform-*d*) δ 7.33 – 7.26 (m, 2H), 6.95 (m, 2H), 6.87 (t, *J* = 7.3 Hz, 1H), 4.99 (m 5.5 Hz, 1H), 4.50 (m, 1H), 3.26 (m, 6H), 2.84 – 2.68 (m, 4H), 2.06 (s, 3H), 1.98 – 1.93 (m, 1H), 1.92 – 1.87 (m, 1H), 1.70 – 1.64 (m, 4H), 1.59 – 1.45 (m, 9H), 1.38 – 1.29 (m, 5H), 1.21 (m, 6H), 1.15 (s, 3H), 1.05 (s, 1H), 0.98 (m, 7H), 0.91 (s, 2H), 0.87 (m, 9H). ^{13}C NMR (126 MHz, $CDCl_3$) δ 171.00, 169.56, 151.38, 129.23, 119.87, 116.27, 80.74, 75.68, 75.25, 71.04, 60.00, 55.91, 53.26, 52.29, 51.85, 49.78, 49.13, 45.36, 39.69, 38.55, 37.99, 37.17, 37.11, 34.38, 33.35, 32.74, 31.13, 29.82, 29.02, 28.78, 28.11, 26.48, 25.28, 23.71, 21.42, 18.39, 18.25, 16.63, 16.10, 15.82. HRMS (ESI, m/z): calculated for $[M + H]^+$ $C_{44}H_{69}N_2O_5$ 706.8922, found: 706.8987.

*(3S,8R,10R,12R,14R)-3-acetoxy-4,4,8,10,14-pentamethyl-17-((R)-2,6,6-trimethyltetrahydro-2H-pyran-2-yl)hexadecahydro-1H-cyclopenta[a]phenanthren-12-yl 2-(4-benzhydrylpiperazin-1-yl)acetate (**13**)*

Compound 10 (100 mg, 0.17 mmol) and 1-benzhydrylpiperazine (65 mg, 0.259 mmol) were added. The resulting crude product was purified by silica gel column chromatography eluted with (petroleum ether/ethyl acetate 5:1), affording compound (3*S*,8*R*,10*R*,12*R*,14*R*)-3-acetoxy-4,4,8,10,14-pentamethyl-17-((*R*)-2,6,6-trimethyltetrahydro-2H-pyran-2-yl)hexadecahydro-1H-cyclopenta[a]phenanthren-12-yl 2-(4-benzhydrylpiperazin-1-yl)acetate (13) as a white amorphous powder (123.01 mg, 91% yield). $[\alpha]_D^{25}$ = + 18.95 (c 1.27, $CHCl_3$). ^{1}H NMR (500 MHz, Chloroform-*d*) δ 7.41 – 7.40 (m, 4H), 7.27 – 7.24 (m, 4H), 7.18 – 7.15 (m, 2H),5.00 – 4.91 (m, 1H), 4.49– 1.97 (m, 1H), 4.23 (s, 1H), 3.19 – 2.91 (m, 2H), 2.73 – 2.30 (m, 8H), 2.04 (s, 3H), 2.02 – 1.97 (m, 1H), 1.93 – 1.88 (m, 1H), 1.88 – 1.81 (m, 1H), 1.76 – 1.69 (m, 2H), 1.62 – 1.60 (m, 4H), 1.54 – 1.51 (m, 2H), 1.49 – 1.41 (m, 6H), 1.30 – 1.25 (m, 3H), 1.17 (s, 6H), 1.11 (s, 3H), 1.02 (s, 1H), 0.96 (s, 3H), 0.91 (s, 3H), 0.87 – 0.81 (m, 12H). ^{13}C NMR (126 MHz, $CDCl_3$) δ 171.02, 169.71, 142.91, 128.58, 128.07, 127.02, 80.76, 76.26, 75.68, 75.13, 71.01, 60.00, 55.92, 53.41, 52.32, 52.07, 51.82, 49.78, 45.35, 39.69, 38.55, 38.01, 37.16, 37.11, 34.81, 34.39, 33.12, 32.87, 31.17, 28.82, 28.12, 27.06, 26.45, 25.42, 25.37, 23.72, 21.44, 18.37, 18.27, 16.64, 16.10, 15.81. HRMS (ESI, m/z): calculated for $[M + H]^+$ $C_{51}H_{75}N_2O_5S$ 796.8533, found: 796.8565.

*(3S,8R,10R,12R,14R)-3-acetoxy-4,4,8,10,14-pentamethyl-17-((R)-2,6,6-trimethyltetrahydro-2H-pyran-2-yl)hexadecahydro-1H-cyclopenta[a]phenanthren-12-yl 2-(4-acetylpiperazin-1-yl)acetate (**14**)*

Compound 10 (100 mg, 0.17 mmol) and 1-acetylpiperazine (33 mg, 0.259 mmol) were added. The resulting crude product was purified by silica gel column chromatography eluted with (dichloromethane/methanol 10:1), affording compound (3*S*,8*R*,10*R*,12*R*,14*R*)-3-acetoxy-4,4,8,10,14-pentamethyl-17-((*R*)-2,6,6-trimethyltetrahydro-2H-pyran-2-yl)hexadecahydro-1H-cyclopenta[a]phenanthren-12-yl 2-(4-acetylpiperazin-1-yl)acetate (14) as a white amorphous powder (95.82 mg, 84% yield). $[\alpha]_D^{25}$ = + 24.89 (c 0.90, $CHCl_3$). ^{1}H NMR (500 MHz, Chloroform-*d*) δ 4.98 – 4.93 (m, 1H), 4.49 – 4.46 (m, 1H), 3.69 – 3.64 (m, 2H), 3.51 – 3.48 (m, 2H), 3.24 – 2.98 (m, 2H), 2.66 – 2.44 (m, 4H), 2.08 (s, 3H), 2.03 (s, 3H), 2.01 – 1.93 (m, 1H), 1.92 – 1.88 (m, 1H), 1.88 – 1.82 (m, 1H), 1.73 – 1.63 (m, 4H), 1.55 – 1.40 (m, 9H), 1.33 – 1.25 (m, 4H), 1.18 – 1.14 (m, 6H), 1.12 (s, 3H), 1.04 – 1.02 (m, 1H), 0.99 – 0.94 (m, 3H), 0.92 (s, 3H), 0.88 – 0.79 (m, 12H). ^{13}C NMR (126 MHz, $CDCl_3$) δ 171.03, 169.45, 169.03, 80.73, 75.66, 75.31, 71.06, 59.74, 55.92, 53.12, 52.82, 52.25, 51.66, 49.76, 46.31, 45.36, 41.42, 39.69, 38.57, 38.00, 37.18, 37.11, 34.37, 33.60, 32.59, 31.07, 29.24, 28.77, 28.11, 27.04, 26.50, 25.15, 23.70, 21.45, 21.43, 18.37, 18.25, 16.63, 16.10, 15.83. HRMS (ESI, m/z): calculated for $[M + H]^+$ $C_{40}H_{67}N_2O_6$ 671.8831, found: 671.8836.

*(3S,8R,10R,12R,14R)-3-acetoxy-4,4,8,10,14-pentamethyl-17-((R)-2,6,6-trimethyltetrahydro-2H-pyran-2-yl)hexadecahydro-1H-cyclopenta[a]phenanthren-12-yl 2-(4-cinnamylpiperazin-1-yl)acetate (**15**)*

Compound 10 (100 mg, 0.17 mmol) and 1-cinnamylpiperazine (53 mg, 0.259 mmol) were added. The resulting crude product was purified by silica gel column chromatography eluted with (petroleum ether/ethyl acetate 1:1), affording compound (3*S*,8*R*,10*R*,12*R*,14*R*)-3-acetoxy-4,4,8,10,14-pentamethyl-17-((*R*)-2,6,6-trimethyltetrahydro-2H-pyran-2-yl)hexadecahydro-1H-cyclopenta[a]phenanthren-12-yl 2-(4-cinnamylpiperazin-1-yl)acetate (15) as a white amorphous powder (79.80 mg, 63% yield). $[\alpha]_D^{25}$ = + 18.68 (c 0.71, $CHCl_3$). ^{1}H NMR (500 MHz, Chloroform-*d*) δ 7.36 (d, *J* = 7.6 Hz, 2H), 7.29 (t, *J* = 7.6 Hz, 2H), 7.21 (t, *J* = 7.3 Hz, 1H), 6.52 (d, *J* = 15.8 Hz, 1H), 6.29 – 6.23 (m, 1H), 4.97– 4.91 (m, 1H), 4.47 (dd, *J* = 11.3, 4.7 Hz, 1H), 3.23 – 3.08 (m, 4H), 2.59 (s, 8H), 2.03 (s, 3H), 2.01 – 1.97 (m, 1H), 1.93 – 1.89 (m, 1H), 1.87 – 1.82 (m, 1H), 1.65 – 1.59 (m, 4H), 1.55 – 1.49 (m, 4H), 1.47 – 1.41 (m, 4H), 1.33 – 1.26 (m, 5H), 1.16 (d, *J* = 2.5 Hz, 6H), 1.11 (s, 3H), 1.02 (s, 1H), 0.95 (s, 3H), 0.91 (s, 3H), 0.89 – 0.79 (m, 12H). ^{13}C NMR (126 MHz, $CDCl_3$) δ 171.26, 171.00, 169.64, 136.97, 133.41, 128.67, 127.64, 126.45, 80.75, 75.66, 75.16, 71.01, 61.06, 60.51, 60.02, 55.91, 53.24, 52.98, 52.28, 51.93, 49.76, 45.34, 39.67, 38.53, 37.98, 37.15, 37.10, 34.37, 32.78, 31.14, 29.82, 28.91, 28.74, 28.10, 26.45, 25.30, 23.70, 21.41, 21.16, 18.37, 18.24, 16.62, 16.09, 15.80, 14.32. HRMS (ESI, m/z): calculated for $[M + H]^+$ $C_{47}H_{73}N_2O_5$ 746.6832, found: 746.6801.

*benzyl 4-(2-(((3S,8R,10R,12R,14R)-3-acetoxy-4,4,8,10,14-pentamethyl-17-((R)-2,6,6-trimethyltetrahydro-2H-pyran-2-yl)hexadecahydro-1H-cyclopenta[a]phenanthren-12-yl)oxy)-2-oxoethyl)piperazine-1-carboxylate (**16**)*

Compound 10 (100 mg, 0.17 mmol) and benzyl piperazine-1-carboxylate (57 mg, 0.259 mmol) were added. The resulting crude product was purified by silica gel column chromatography eluted with (petroleum ether/ethyl acetate 3:1), affording benzyl 4-(2-(((3*S*,8*R*,10*R*,12*R*,14*R*)-3-acetoxy-4,4,8,10,14-pentamethyl-17-((*R*)-2,6,6-trimethyltetrahydro-2H-pyran-2-yl)hexadecahydro-1H-cyclopenta[a]phenanthren-12-yl)oxy)-2-oxoethyl)piperazine-1-carboxylate (16) as a white amorphous powder (101.18 mg, 78% yield). $[\alpha]_D^{25}$ = + 4.85 (c 0.91, $CHCl_3$). ^{1}H NMR (500 MHz, Chloroform-*d*) δ 7.44

– 7.27 (m, 5H), 5.12 (s, 2H), 4.98 – 4.93 (m, 1H), 4.50 – 4.46 (m, 1H), 3.55 (s, 4H), 3.26 – 3.02 (m, 2H), 2.56 – 2.41 (m, 4H), 2.07 – 2.05 (m, 1H), 2.04 (s, 3H), 1.95 – 1.88 (m, 1H), 1.87 – 1.83 (m, 1H), 1.74 – 1.63 (m, 4H), 1.54 – 1.42 (m, 8H), 1.34 – 1.23 (m, 4H), 1.21 – 1.16 (m, 6H), 1.12 (s, 3H), 1.05 – 1.00 (m, 2H), 0.97 – 0.94 (m, 3H), 0.93 (s, 3H), 0.90 – 0.77 (m, 12H). ^{13}C NMR (126 MHz, $CDCl_3$) δ 171.02, 169.51, 155.34, 136.84, 128.63, 128.15, 128.04, 80.73, 75.66, 75.27, 71.05, 67.27, 59.94, 55.92, 52.85, 52.26, 51.73, 49.76, 45.36, 43.80, 39.69, 38.56, 38.00, 37.17, 37.11, 34.37, 33.51, 32.64, 31.09, 29.16, 28.76, 28.11, 27.05, 26.49, 25.20, 23.70, 21.43, 18.37, 18.25, 16.63, 16.10, 15.83. HRMS (ESI, m/z): calculated for $[M + H]^+$ $C_{46}H_{71}N_2O_7$ 764.4809, found: 764.4871.

*(3S,8R,10R,12R,14R)-3-acetoxy-4,4,8,10,14-pentamethyl-17-((R)-2,6,6-trimethyltetrahydro-2H-pyran-2-yl)hexadecahydro-1H-cyclopenta[a]phenanthren-12-yl 2-(4-cyclohexylpiperazin-1-yl)acetate (**17**)*

Compound 10 (100 mg, 0.17 mmol) and 1-cyclohexylpiperazine (44 mg, 0.259 mmol) were added. The resulting crude product was purified by silica gel column chromatography eluted with (dichloromethane/methanol 10:1), affording compound (3*S*,8*R*,10*R*,12*R*,14*R*)-3-acetoxy-4,4,8,10,14-pentamethyl-17-((*R*)-2,6,6-trimethyltetrahydro-2H-pyran-2-yl)hexadecahydro-1H-cyclopenta[a]phenanthren-12-yl 2-(4-cyclohexylpiperazin-1-yl)acetate (17) as a white amorphous powder (83.41 mg, 69% yield). $[\alpha]_D^{25}$ = + 19 (c 0.80, $CHCl_3$). ^{1}H NMR (500 MHz, Chloroform-*d*) δ 5.00 – 4.91 1H), 4.49 – 4.46 (m, 1H), 3.11 (s, 2H), 2.70 – 2.52 (m, 8H), 2.24 (s, 1H), 2.03 (s, 3H), 2.00 – 1.98 (m, 1H), 1.93 – 1.84 (m, 5H), 1.81 – 1.68 (m, 6H), 1.66 – 1.61 (m, 4H), 1.55 – 1.51 (m, 4H), 1.48 – 1.40 (m, 6H), 1.30 – 1.24 (m, 6H), 1.21– 1.19 (m, 4H), 1.10 (s, 3H), 0.95 (s, 3H), 0.91 (s, 3H), 0.88 – 0.81 (m, 13H). ^{13}C NMR (126 MHz, $CDCl_3$) δ 171.01, 169.71, 80.77, 77.41, 77.16, 76.91, 75.68, 75.14, 71.01, 63.65, 60.13, 55.92, 53.82, 52.33, 52.00, 49.79, 48.74, 45.35, 39.69, 38.54, 37.99, 37.15, 37.11, 34.79, 34.39, 32.85, 31.18, 29.08, 29.01, 28.84, 28.76, 28.11, 27.04, 26.46, 26.41, 26.01, 25.41, 23.71, 21.42, 18.39, 18.26, 16.62, 16.10, 15.80. HRMS (ESI, m/z): calculated for $[M + H]^+$ $C_{44}H_{75}N_2O_5$ 712.5573, found: 712.5538.

*(3S,8R,10R,12R,14R)-3-acetoxy-4,4,8,10,14-pentamethyl-17-((R)-2,6,6-trimethyltetrahydro-2H-pyran-2-yl)hexadecahydro-1H-cyclopenta[a]phenanthren-12-yl 2-(3-oxopiperazin-1-yl)acetate (**18**)*

Compound 10 (100 mg, 0.17 mmol) and piperazin-2-one (26 mg, 0.259 mmol) were added. The resulting crude product was purified by silica gel column chromatography eluted with (dichloromethane/methanol 10:1), affording compound (3*S*,8*R*,10*R*,12*R*,14*R*)-3-acetoxy-4,4,8,10,14-pentamethyl-17-((*R*)-2,6,6-trimethyltetrahydro-2H-pyran-2-yl)hexadecahydro-1H-cyclopenta[a]phenanthren-12-yl 2-(3-oxopiperazin-1-yl)acetate (18) as a white amorphous powder (79.79 mg, 73% yield). $[\alpha]_D^{25}$ = + 20 (c 0.72, $CHCl_3$). ^{1}H NMR (500 MHz, Chloroform-*d*) δ 6.04 (s, 1H), 5.01 – 4.95 (m, 1H), 4.50 – 4.47 (m, 1H), 3.45 – 3.16 (m, 6H), 2.92 – 2.76 (m, 2H), 2.13 – 2.06 (m, 1H), 2.04 (s, 3H), 2.02 – 1.95 (m, 1H), 1.93 – 1.88 (m, 1H), 1.88 – 1.82 (m, 1H), 1.78 – 1.70 (m, 2H), 1.67 – 1.63 (m, 3H), 1.56 – 1.53 (m, 3H), 1.49 – 1.41 (m, 5H), 1.35 – 1.25 (m, 5H), 1.18 – 1.16 (m, 4H), 1.14 – 1.10 (m, 3H), 1.04 – 1.01 (m, 1H), 0.98 – 0.96 (m, 3H), 0.95 – 0.92 (m, 3H), 0.89 – 0.82 (m, 12H). ^{13}C NMR (126 MHz, $CDCl_3$) δ 169.89, 167.95, 123.36, 79.58, 76.26, 76.00, 75.75, 74.50, 74.24, 69.94, 57.15, 55.32, 54.77, 51.01, 50.28, 48.57, 47.63, 44.21, 40.41, 38.54, 37.41, 36.86, 36.06, 33.21, 31.23, 29.80, 28.39, 26.97, 25.35, 23.83, 22.54, 20.28, 17.19, 17.09, 15.49, 15.46, 14.93, 14.71. HRMS (ESI, m/z): calculated for $[M + H]^+$ $C_{38}H_{63}N_2O_6$ 643.4908, found: 643.4992.

*(3S,8R,10R,12R,14R)-3-acetoxy-4,4,8,10,14-pentamethyl-17-((R)-2,6,6-trimethyltetrahydro-2H-pyran-2-yl)hexadecahydro-1H-cyclopenta[a]phenanthren-12-yl 2-(4-(2-chloropyridin-4-yl)piperazin-1-yl)acetate (**19**)*

Compound 10 (100 mg, 0.17 mmol) and 1-(2-chloropyridin-4-yl)piperazine (51 mg, 0.259 mmol) were added. The resulting crude product was purified by silica gel column chromatography eluted with (petroleum ether/ethyl acetate 2:1), affording compound (3*S*,8*R*,10*R*,12*R*,14*R*)-3-acetoxy-4,4,8,10,14-pentamethyl-17-((*R*)-2,6,6-trimethyltetrahydro-2H-pyran-2-yl)hexadecahydro-1H-cyclopenta[a]phenanthren-12-yl 2-(4-(2-chloropyridin-4-yl)piperazin-1-yl)acetate (19) as a white amorphous powder (95.67 mg, 76% yield). $[\alpha]_D^{25}$ = + 26.32 (c 1.03, $CHCl_3$). ^{1}H NMR (500 MHz, Chloroform-*d*) δ 8.03 (d, *J* = 5.9 Hz, 1H), 6.68 – 6.62 (m, 1H), 6.57 (dd, *J* = 5.9, 2.4 Hz, 1H), 5.02 – 4.93 (m, 1H), 4.48 (dd, *J* = 11.2, 4.5 Hz, 1H), 3.54 – 3.19 (m, 6H), 2.85 – 2.67 (m, 4H), 2.04 (s, 3H), 1.71 – 1.64 (m, 7H), 1.55 – 1.46 (m, 9H), 1.16 (s, 3H), 1.13 (s, 3H), 1.07– 1.03(m, 2H), 0.97 – 0.92 (m, 6H), 0.88 (d, *J* = 6.3 Hz, 6H), 0.87 – 0.84 (m, 12H). ^{13}C NMR (126 MHz, $CDCl_3$) δ 174.50, 171.07, 157.22, 149.60, 147.48, 120.94, 107.54, 80.73, 80.67, 76.62, 75.71, 68.40, 67.92, 64.98, 64.50, 64.12, 61.13, 57.50, 55.92, 53.65, 52.23, 49.77, 45.37, 44.96, 41.11, 41.08, 39.71, 38.59, 38.02, 37.20, 37.12, 29.85, 28.13, 26.53, 23.71, 22.84, 21.45, 18.38, 18.25, 16.65, 16.11, 15.87, 14.27. HRMS (ESI, m/z): calculated for $[M + H]^+$ $C_{43}H_{67}ClN_3O_5$ 741.8601, found: 741.8647.

*ethyl 4-(2-(((3S,8R,10R,12R,14R)-3-acetoxy-4,4,8,10,14-pentamethyl-17-((R)-2,6,6-trimethyltetrahydro-2H-pyran-2-yl)hexadecahydro-1H-cyclopenta[a]phenanthren-12-yl)oxy)-2-oxoethyl)piperazine-1-carboxylate (**20**)*

Compound 10 (100 mg, 0.17 mmol) and ethyl piperazine-1-carboxylate (41 mg, 0.259 mmol) were added. The resulting crude product was purified by silica gel column chromatography eluted with (petroleum ether/ethyl acetate 3:1), affording ethyl 4-(2-(((3*S*,8*R*,10*R*,12*R*,14*R*)-3-acetoxy-4,4,8,10,14-pentamethyl-17-((*R*)-2,6,6-trimethyltetrahydro-2H-pyran-2-yl)hexadecahydro-1H-cyclopenta[a]phenanthren-12-yl)oxy)-2-oxoethyl)piperazine-1-carboxylate (20) as a white amorphous powder (92.95 mg, 78% yield). $[\alpha]_D^{25}$ = + 20.33 (c 0.81, $CHCl_3$). ^{1}H NMR (500 MHz, Chloroform-*d*) δ 5.01 – 4.92 (m, 1H), 4.49 –4.43 (m, 1H), 4.12 (q, *J* = 7.1 Hz, 2H), 3.51 (s, 4H), 3.24 – 3.05 (m, 2H), 2.60 – 2.45 (m, 4H), 2.03 (s, 3H), 1.92 – 1.87 (m, 1H), 1.86 – 1.82 (m, 1H), 1.78 – 1.68 (m, 3H), 1.67 – 1.58 (m, 5H), 1.56 – 1.43 (m, 9H), 1.41 (s, 1H), 1.28 – 1.23 (m, 6H), 1.18 – 1.13 (s, 5H), 1.13 – 1.09 (m, 3H), 1.03 – 0.99 (m, 1H), 0.95 (t, *J* = 3.2 Hz, 3H), 0.96 – 0.94 (m, 3H), 0.88 – 0.87 (m, 1H), 0.85 – 0.81 (m, 12H). ^{13}C NMR (126 MHz, $CDCl_3$) δ 170.99, 169.47, 155.58, 80.71, 75.64, 75.26, 71.03, 61.46, 59.91, 55.90, 52.83, 52.24, 51.70, 49.75, 45.34, 43.62, 39.67, 38.54, 37.98, 37.15, 37.09, 34.78, 34.36, 33.49, 32.62, 31.08, 29.14, 28.75, 28.09, 27.02, 26.47, 25.39, 25.17, 23.68, 21.40, 18.36, 18.23, 16.61, 16.07, 15.81, 14.78. HRMS (ESI, m/z): calculated for $[M + H]^+$ $C_{41}H_{69}N_2O_7$ 801.9981, found: 801.9997.

*(3S,8R,10R,12R,14R)-3-acetoxy-4,4,8,10,14-pentamethyl-17-((R)-2,6,6-trimethyltetrahydro-2H-pyran-2-yl)hexadecahydro-1H-cyclopenta[a]phenanthren-12-yl 2-(4-(benzo[d][1,3]dioxol-5-ylmethyl)piperazin-1-yl)acetate (**21**)*

Compound 10 (100 mg, 0.17 mmol) and 1-(benzo[d][1,3]dioxol-5-ylmethyl)piperazine (57 mg, 0.259 mmol) were added. The resulting crude product was purified by silica gel column chromatography eluted with (petroleum ether/ethyl acetate 3:1), affording compound (3*S*,8*R*,10*R*,12*R*,14*R*)-3-acetoxy-4,4,8,10,14-pentamethyl-17-((*R*)-2,6,6-trimethyltetrahydro-2H-pyran-2-

yl)hexadecahydro-1H-cyclopenta[a]phenanthren-12-yl 2-(4-(benzo[d][1,3]dioxol-5-ylmethyl)piperazin-1-yl)acetate (21) as a white amorphous powder (94.70 mg, 73% yield). $[\alpha]_D^{25}$ = + 17.32 (c 0.99, $CHCl_3$). ^1H NMR (500 MHz, Chloroform-*d*) δ 6.84 (m, 1H), 6.72 (m, 2H), 5.92 (s, 2H), 4.94 (m, 1H), 4.47 (m, 1H), 3.40 (m, 2H), 3.13 (s, 1H), 2.62 – 2.42 (m, 7H), 2.03 (s, 3H), 2.00 – 1.88 (m, 3H), 1.87 – 1.82 (m, 1H), 1.72 (m, 2H), 1.61 (m, 5H), 1.56 – 1.49 (m, 4H), 1.49 – 1.41 (m, 4H), 1.31 – 1.24 (m, 5H), 1.16 (s, 4H), 1.10 (s, 2H), 1.01 (s, 1H), 0.98 – 0.89 (m, 6H), 0.88 – 0.79 (m, 11H). ^{13}C NMR (126 MHz, $CDCl_3$) δ 171.25, 170.99, 169.70, 147.71, 146.68, 122.38, 109.65, 107.94, 100.97, 80.74, 75.66, 75.12, 71.00, 62.79, 60.51, 60.01, 55.91, 53.25, 52.79, 52.30, 51.97, 49.77, 45.33, 39.67, 38.53, 37.98, 37.27, 37.14, 37.09, 34.37, 32.82, 31.16, 28.85, 28.76, 28.10, 26.45, 25.35, 23.70, 21.41, 21.16, 18.36, 18.24, 16.62, 16.08, 15.79, 14.32. HRMS (ESI, m/z): calculated for $[M + H]^+$ $C_{46}H_{71}N_2O_7$ 764.0407, found: 764.0417.

*(3S,8R,10R,12R,14R)-3-acetoxy-4,4,8,10,14-pentamethyl-17-((R)-2,6,6-trimethyltetrahydro-2H-pyran-2-yl)hexadecahydro-1H-cyclopenta[a]phenanthren-12-yl 2-(4-isobutylpiperazin-1-yl)acetate (**22**)*

Compound 10 (100 mg, 0.17 mmol) and 1-isobutylpiperazine (37 mg, 0.259 mmol) were added. The resulting crude product was purified by silica gel column chromatography eluted with (petroleum ether/ethyl acetate 3:1), affording compound (3*S*,8*R*,10*R*,12*R*,14*R*)-3-acetoxy-4,4,8,10,14-pentamethyl-17-((*R*)-2,6,6-trimethyltetrahydro-2H-pyran-2-yl)hexadecahydro-1H-cyclopenta[a]phenanthren-12-yl 2-(4-isobutylpiperazin-1-yl)acetate (22) as a white amorphous powder (87.34 mg, 75% yield). $[\alpha]_D^{25}$ = + 19.32 (c 1.18, $CHCl_3$). ^1H NMR (500 MHz, Chloroform-*d*) δ 4.95 (m, 1H), 4.47 (m, 1H), 3.21 – 3.04 (m, 2H), 2.54 (m, 8H), 2.08 (d, *J* = 6.9 Hz, 2H), 2.03 (s, 3H), 2.00 (m, 1H), 1.93 – 1.89 (m, 1H), 1.86 (m, 1H), 1.79 – 1.69 (m, 4H), 1.62 – 1.59 (m, 2H), 1.56 – 1.50 (m, 4H), 1.48 – 1.41 (m, 5H), 1.29 – 1.24 (m, 4H), 1.17 (s, 6H), 1.11 (s, 3H), 0.95 (s, 3H), 0.91 (s, 3H), 0.87 (m, 9H), 0.84 (m, 9H). ^{13}C NMR (126 MHz, $CDCl_3$) δ 171.00, 169.74, 80.75, 75.69, 75.11, 71.01, 66.95, 60.09, 55.92, 53.41, 53.33, 52.34, 52.01, 49.79, 45.35, 39.69, 38.54, 37.99, 37.15, 37.10, 34.79, 34.39, 32.86, 31.20, 28.80, 28.10, 27.04, 26.47, 25.47, 25.43, 25.40, 23.71, 21.42, 21.07, 21.05, 18.38, 18.26, 16.62, 16.09, 15.80. HRMS (ESI, m/z): calculated for $[M + H]^+$ $C_{42}H_{73}N_2O_5$ 686.7603, found: 686.7607.

*(3S,8R,10R,12R,14R)-3-acetoxy-4,4,8,10,14-pentamethyl-17-((R)-2,6,6-trimethyltetrahydro-2H-pyran-2-yl)hexadecahydro-1H-cyclopenta[a]phenanthren-12-yl 2-(4-cyclopropylpiperazin-1-yl)acetate (**23**)*

Compound 10 (100 mg, 0.17 mmol) and 1-cyclopropylpiperazine (33 mg, 0.259 mmol) were added. The resulting crude product was purified by silica gel column chromatography eluted with (petroleum ether/ethyl acetate 3:1), affording compound (3*S*,8*R*,10*R*,12*R*,14*R*)-3-acetoxy-4,4,8,10,14-pentamethyl-17-((*R*)-2,6,6-trimethyltetrahydro-2H-pyran-2-yl)hexadecahydro-1H-cyclopenta[a]phenanthren-12-yl 2-(4-cyclopropylpiperazin-1-yl)acetate (23) as a white amorphous powder (68.24 mg, 60% yield). $[\alpha]_D^{25}$ = + 17.95 (c 0.85, $CHCl_3$). ^1H NMR (500 MHz, Chloroform-*d*) δ 5.00 – 4.91 (m, 1H), 4.49 – 4.46 (m, 1H), 3.18 – 2.90 (m, 2H), 2.82 – 2.32 (m, 8H), 2.04 (s, 3H), 2.02 – 1.99 (m, 1H), 1.94 – 1.89 (m, 1H), 1.88 – 1.82 (m, 1H), 1.79 – 1.57 (m, 9H), 1.56 – 1.51 (m, 3H), 1.50 – 1.42 (m, 4H), 1.34 – 1.23 (m, 5H), 1.22 – 1.01 (m, 9H), 0.99 – 0.88 (m, 6H), 0.88 – 0.81 (m, 9H), 0.53 – 0.27 (m, 4H). ^{13}C NMR (126 MHz, $CDCl_3$) δ 171.03, 169.71, 80.77, 75.68, 75.15, 71.02, 60.09, 55.92, 53.25, 53.13, 52.33, 51.98, 50.39, 49.79, 45.35, 40.06, 39.69, 38.55, 38.53, 38.00, 37.29, 37.15, 37.11, 34.39, 32.83, 31.18, 28.86, 28.77, 28.11, 26.47, 25.40, 23.71, 21.43, 18.39, 18.26, 16.63, 16.10, 15.81, 14.33, 5.80. HRMS (ESI, m/z): calculated for $[M + H]^+$ $C_{41}H_{69}N_2O_5$ 670.0056, found: 670.0001.

5. ^{1}H and ^{13}C spectra of the products

Figure S25. ^{1}H NMR Spectrum of compound **1** (500 MHz, $CDCl_3$)

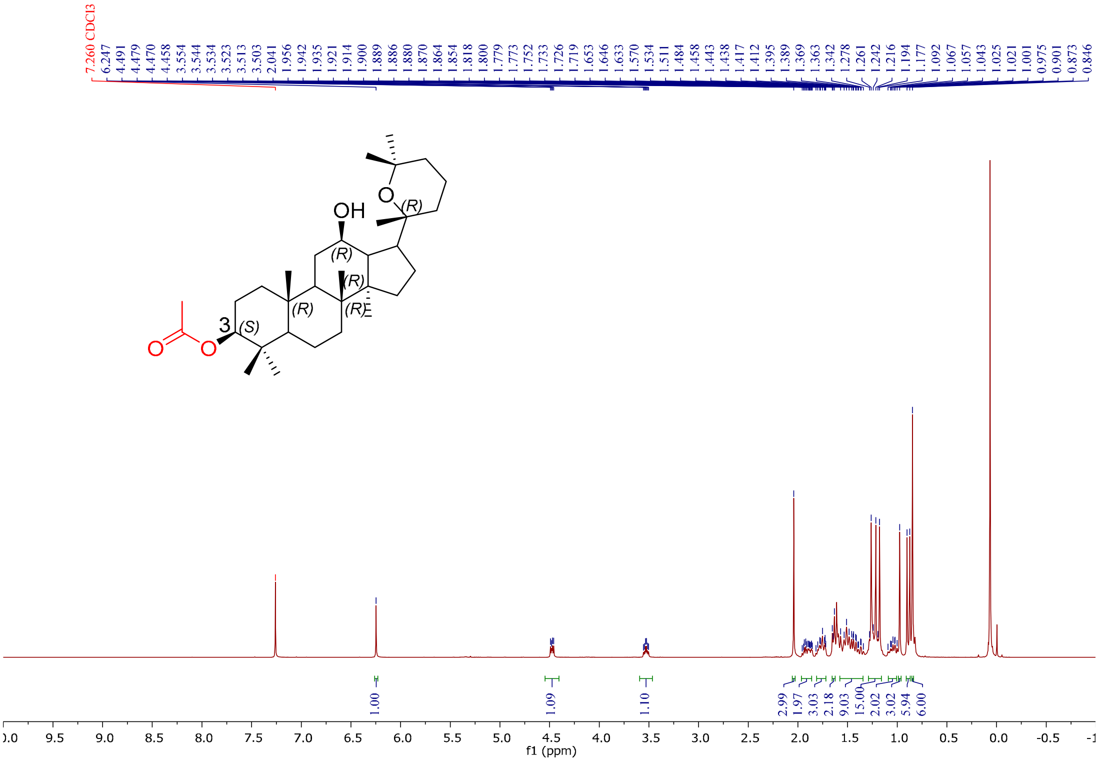

Figure S26. ^{13}C NMR Spectrum of compound **1** (126 MHz, $CDCl_3$)

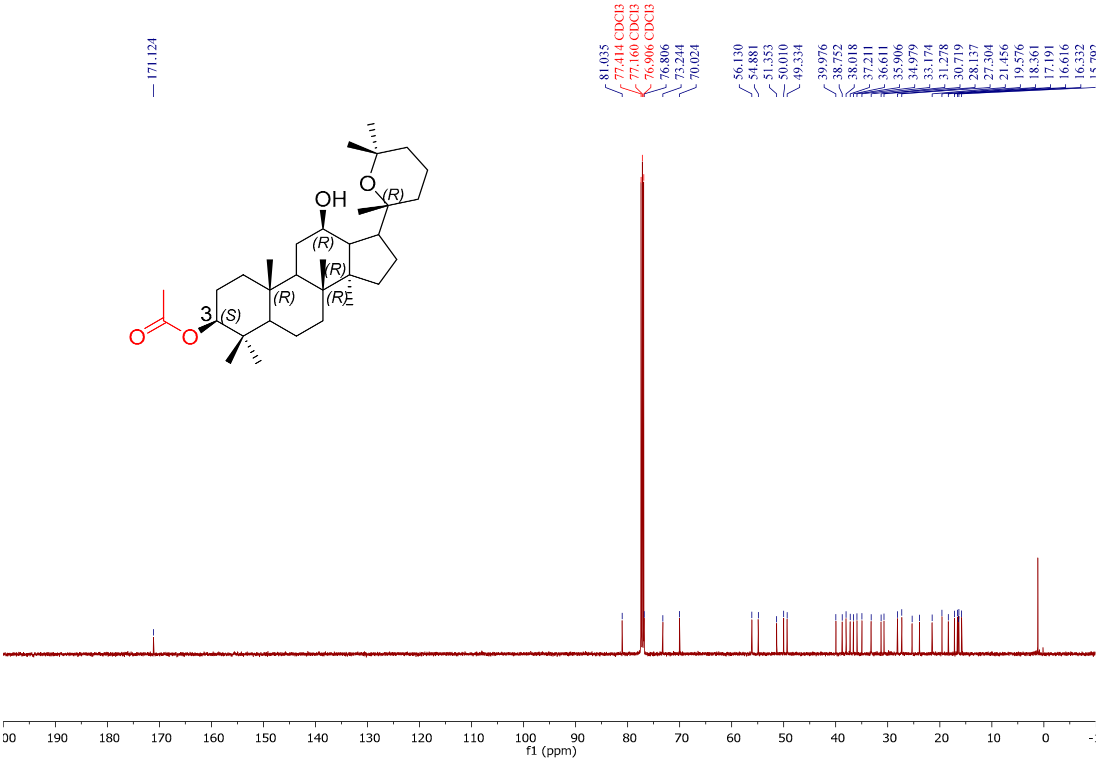

Figure S27. ^{1}H NMR Spectrum of compound **2** (500 MHz, $CDCl_3$)

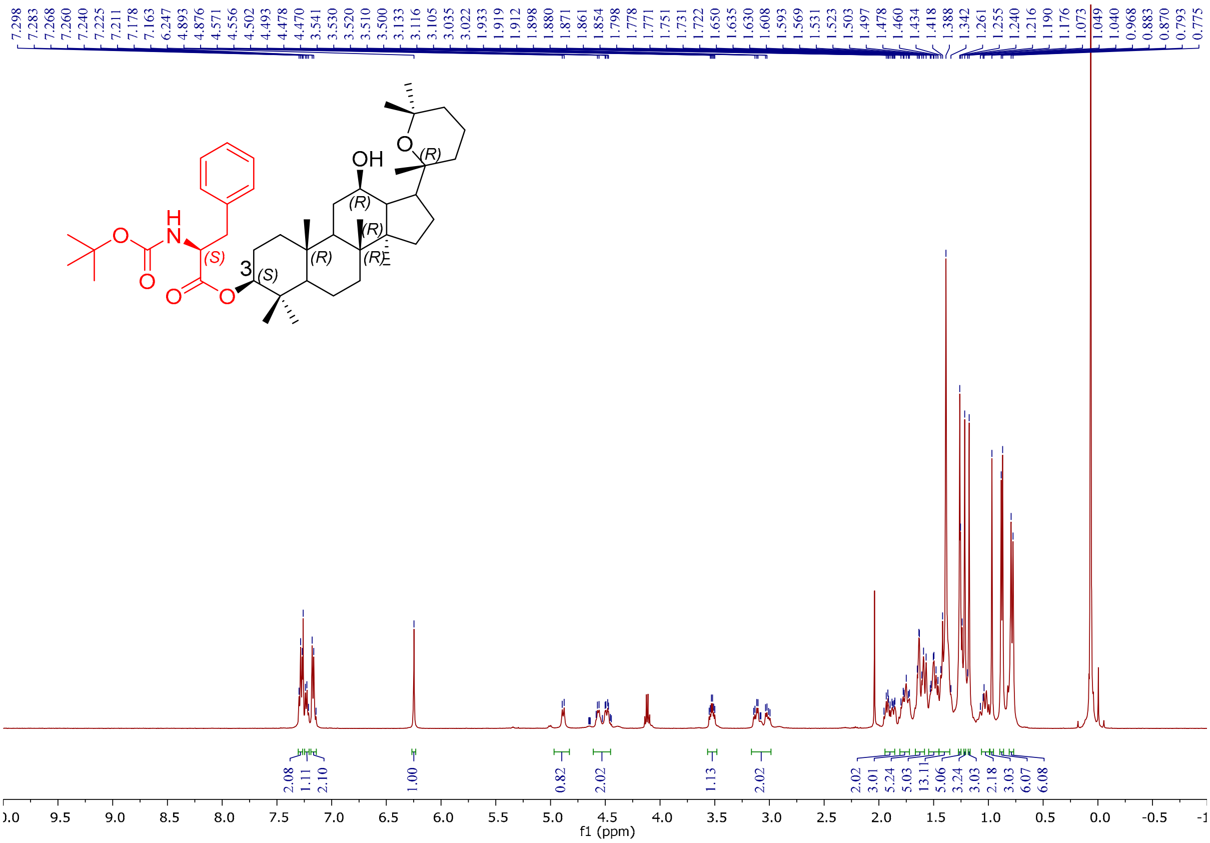

Figure S28. ^{13}C NMR Spectrum of compound **2** (126 MHz, $CDCl_3$)

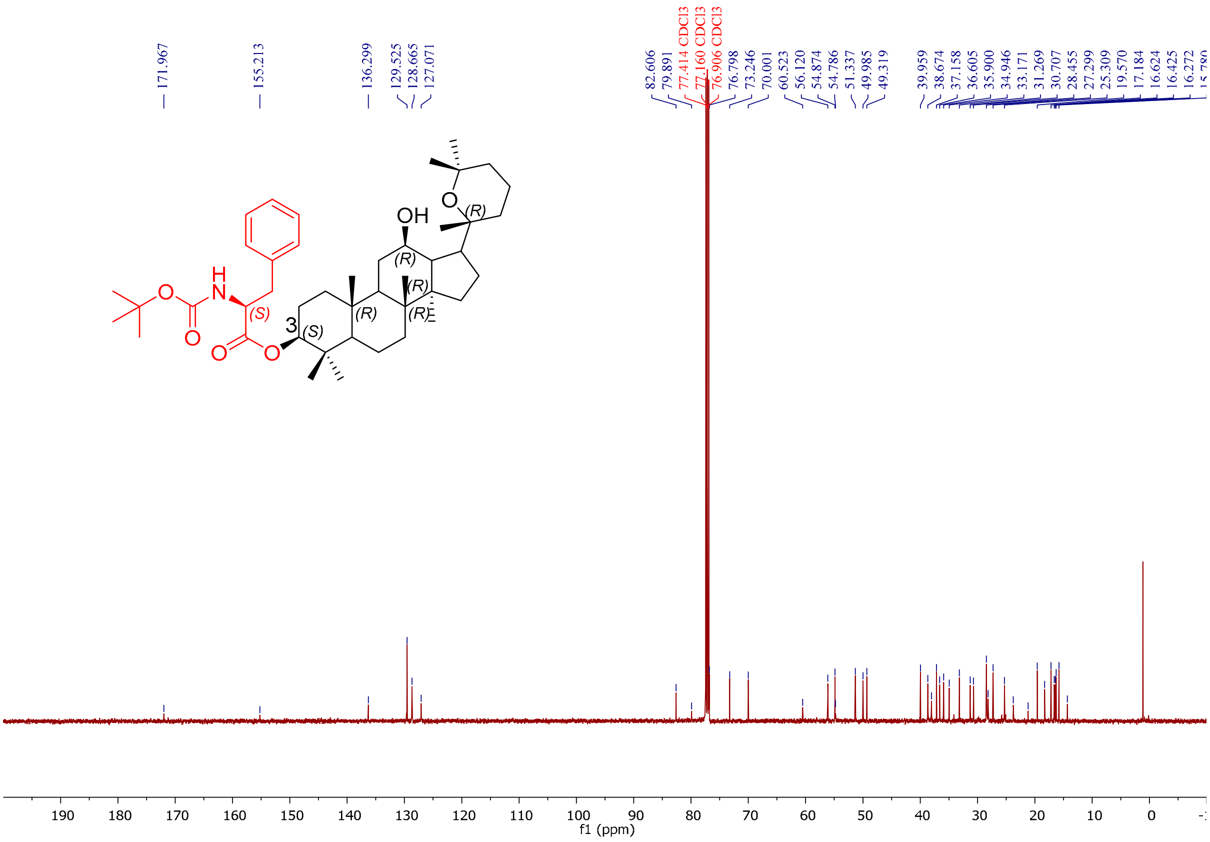

Figure S29. ^{1}H NMR Spectrum of compound **3** (500 MHz, $CDCl_3$)

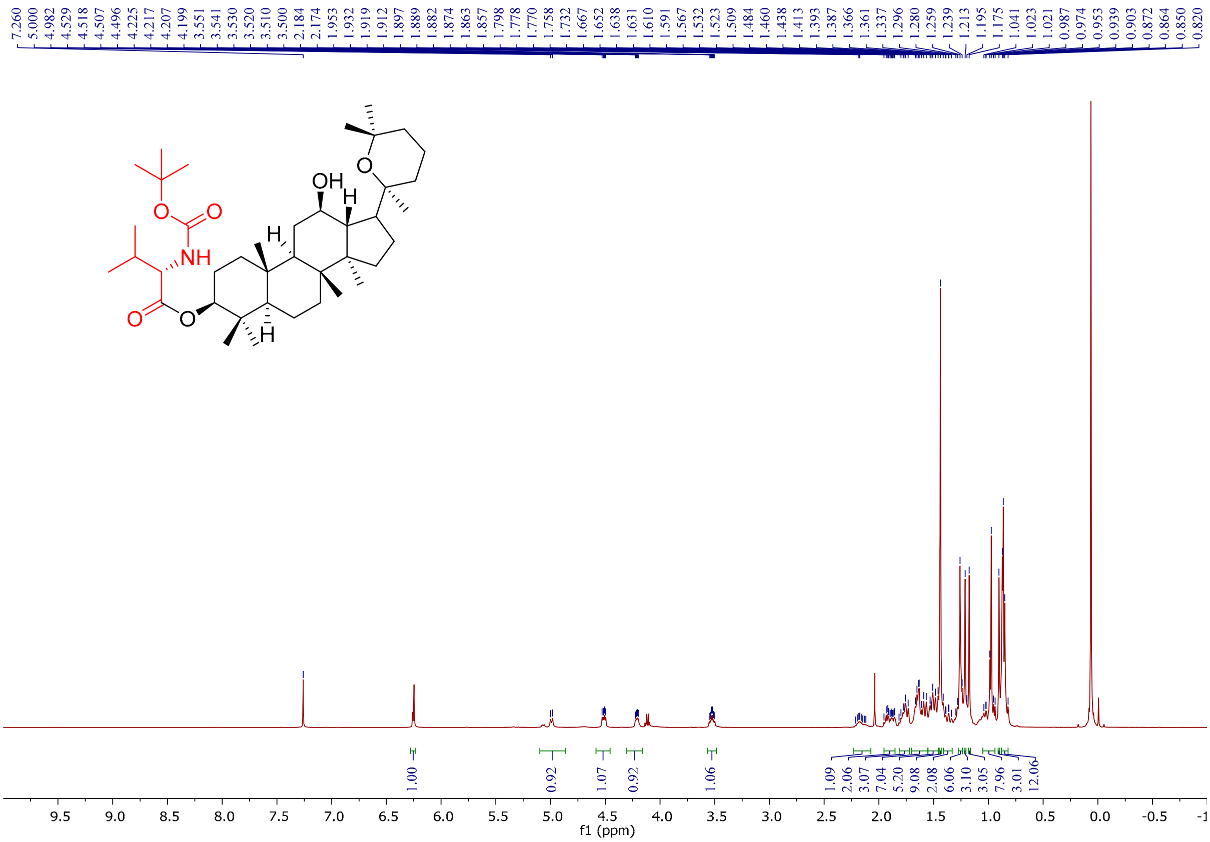

Figure S30. ^{13}C NMR Spectrum of compound **3** (126 MHz, $CDCl_3$)

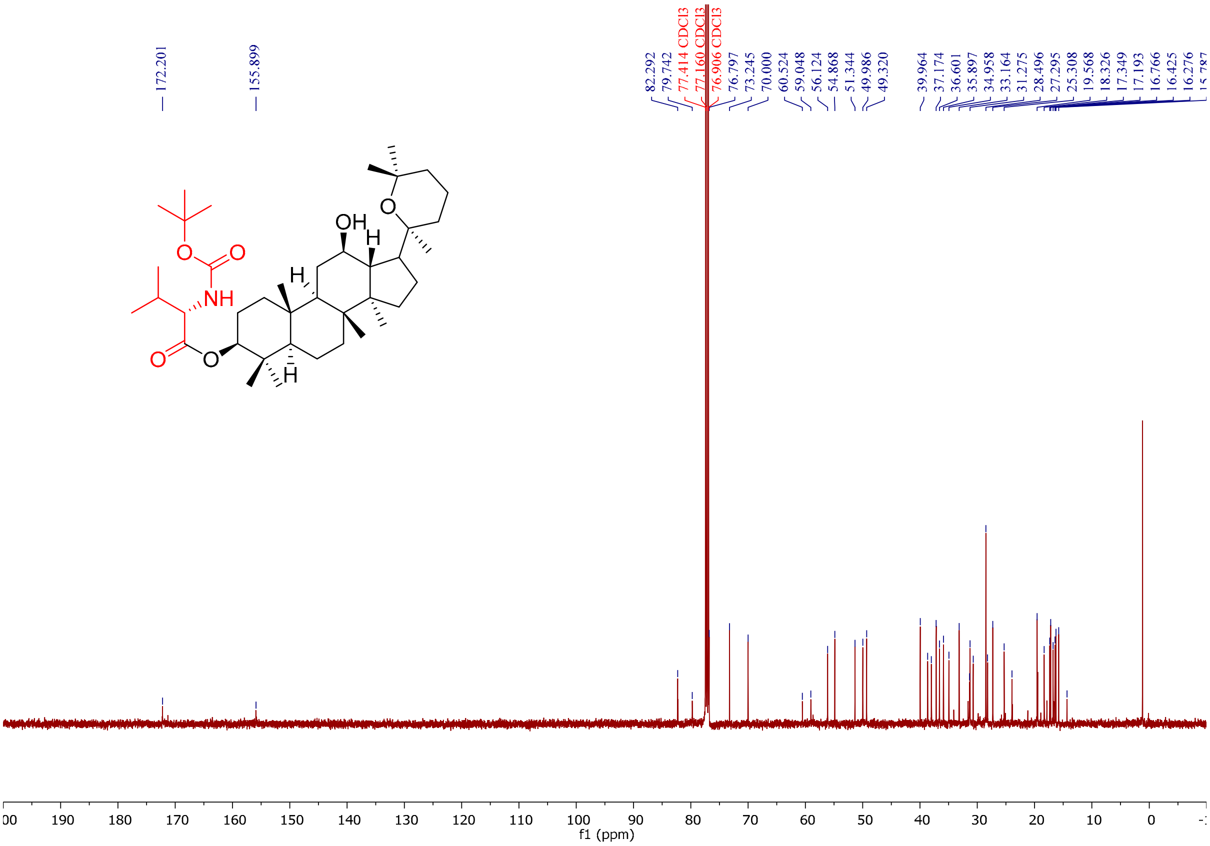

Figure S31. ^{1}H NMR Spectrum of compound **4** (500 MHz, $CDCl_3$)

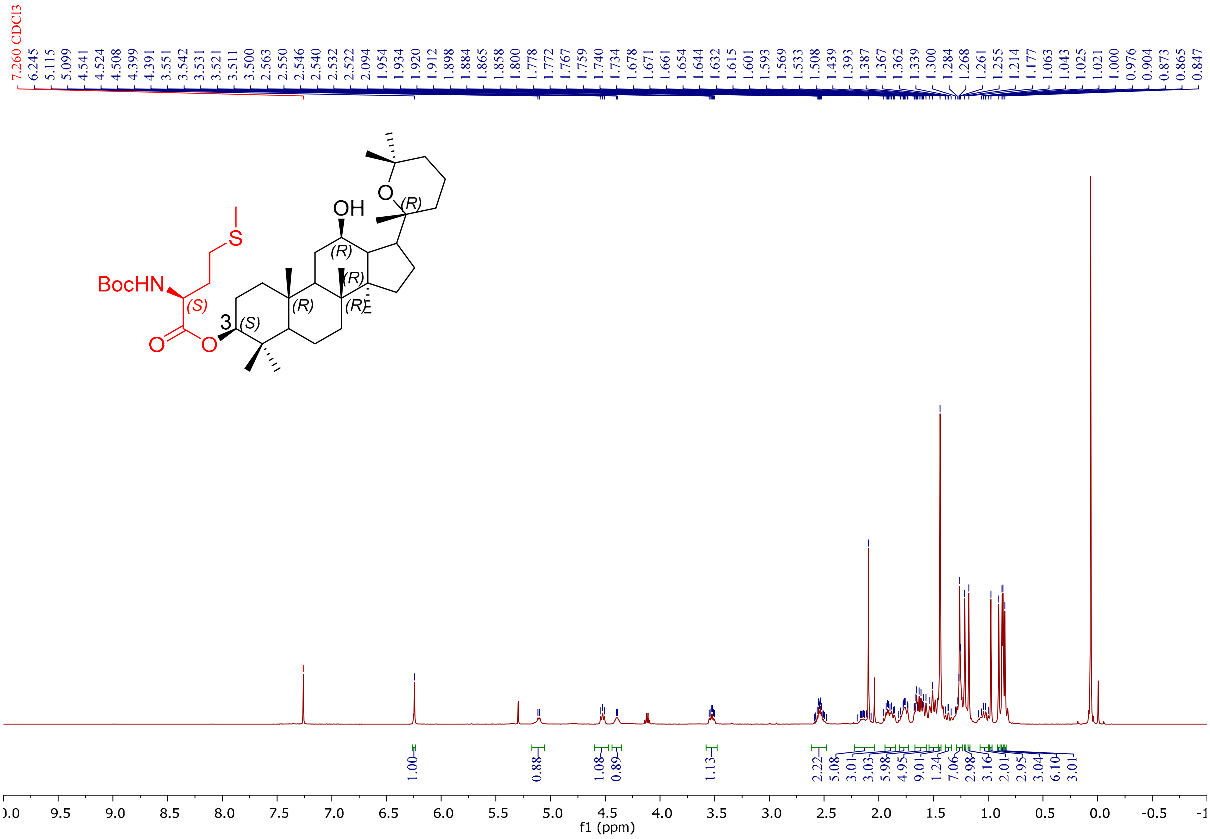

Figure S32. ^{13}C NMR Spectrum of compound **4** (126 MHz, $CDCl_3$)

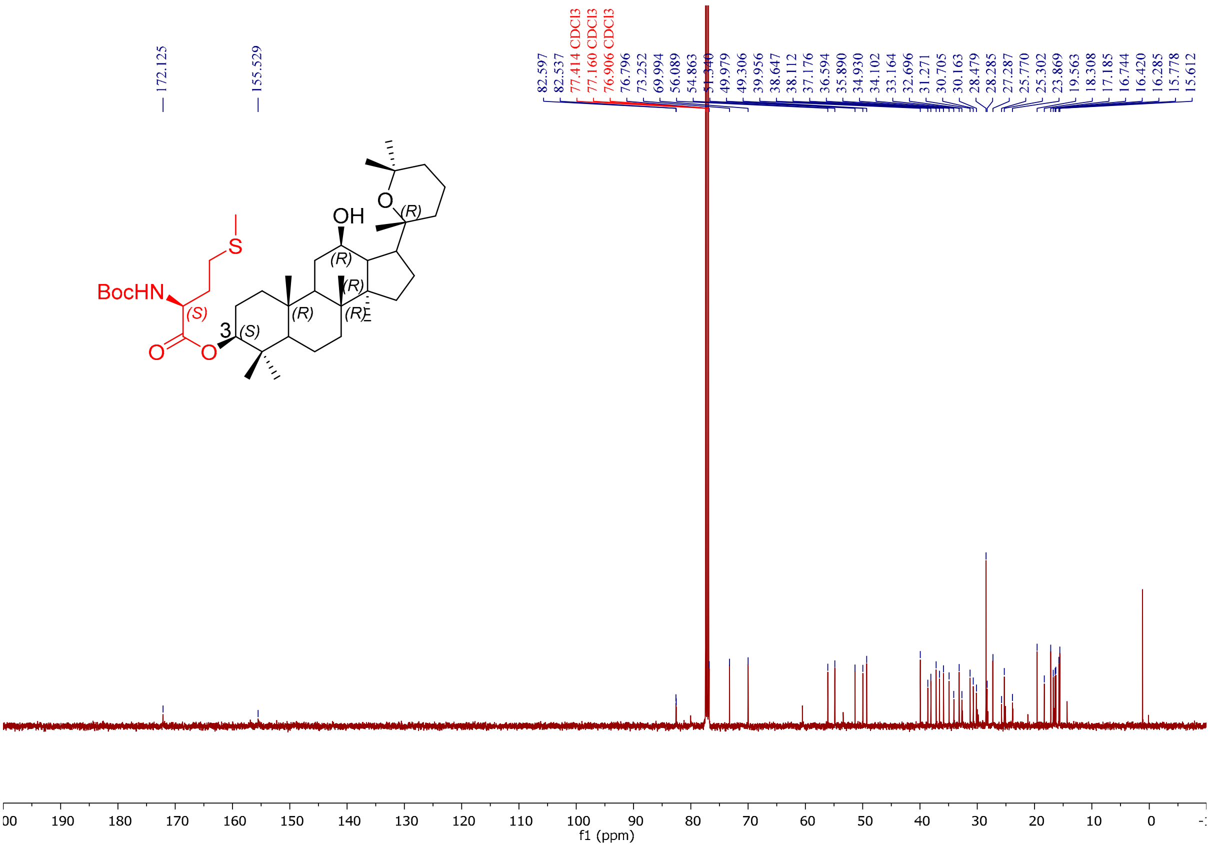

Figure S33. ^{1}H NMR Spectrum of compound **5** (500 MHz, CDCl$_3$)

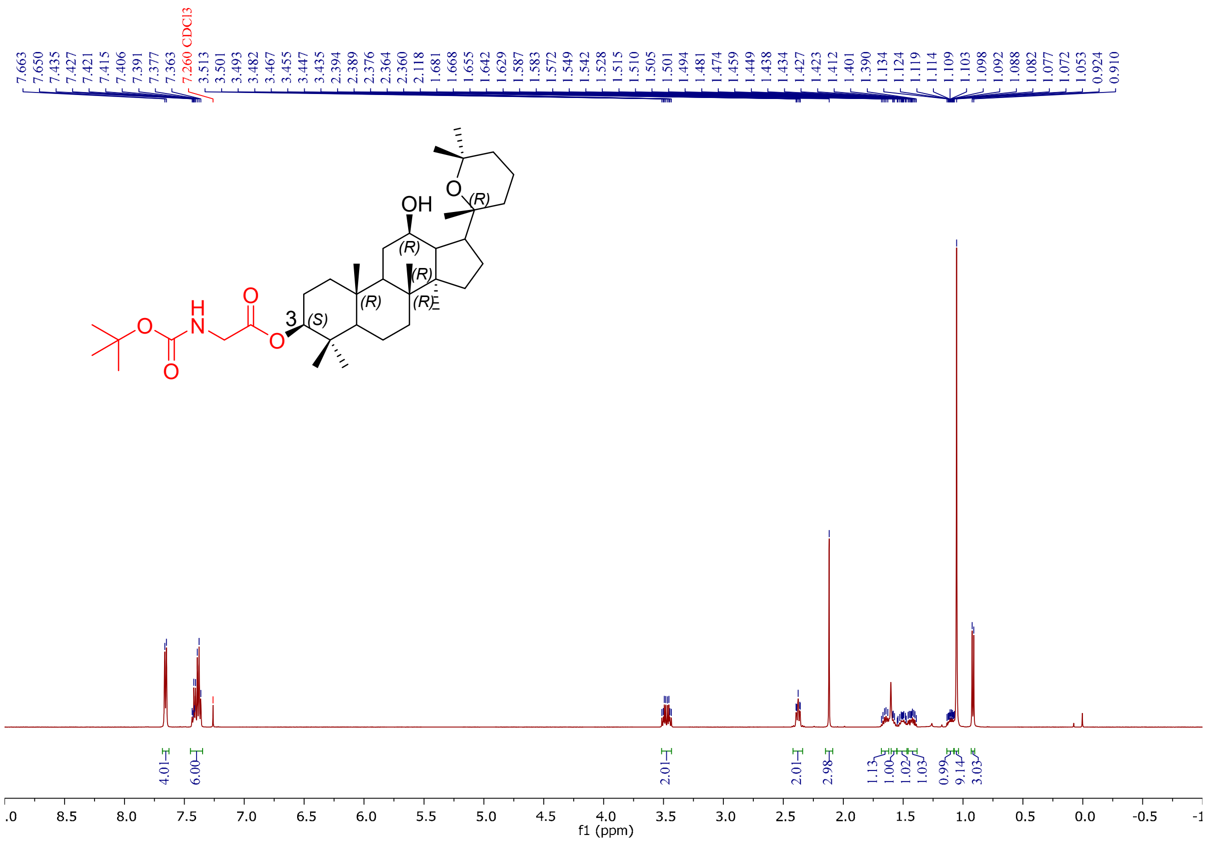

Figure S34. ^{13}C NMR Spectrum of compound **5** (126 MHz, CDCl$_3$)

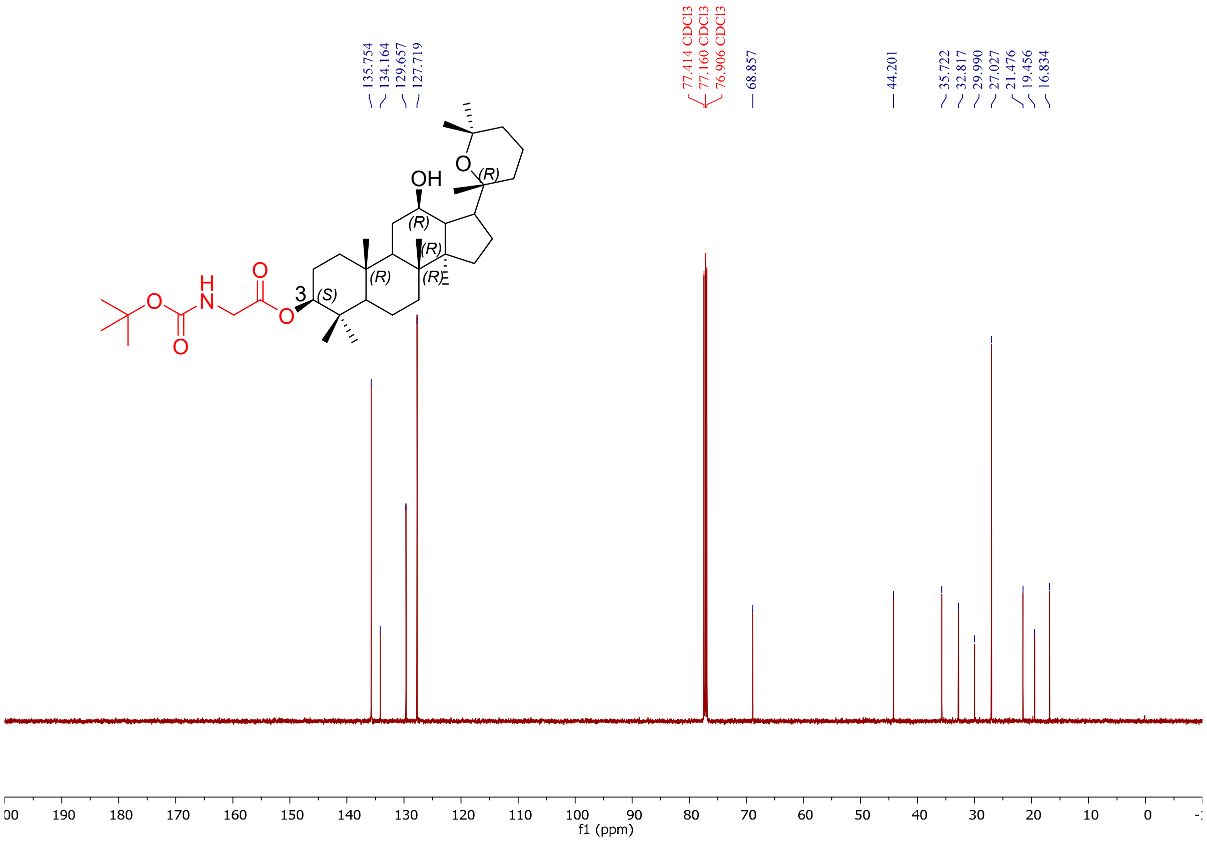

Figure S35. ^{1}H NMR Spectrum of compound **6** (500 MHz, CDCl$_3$)

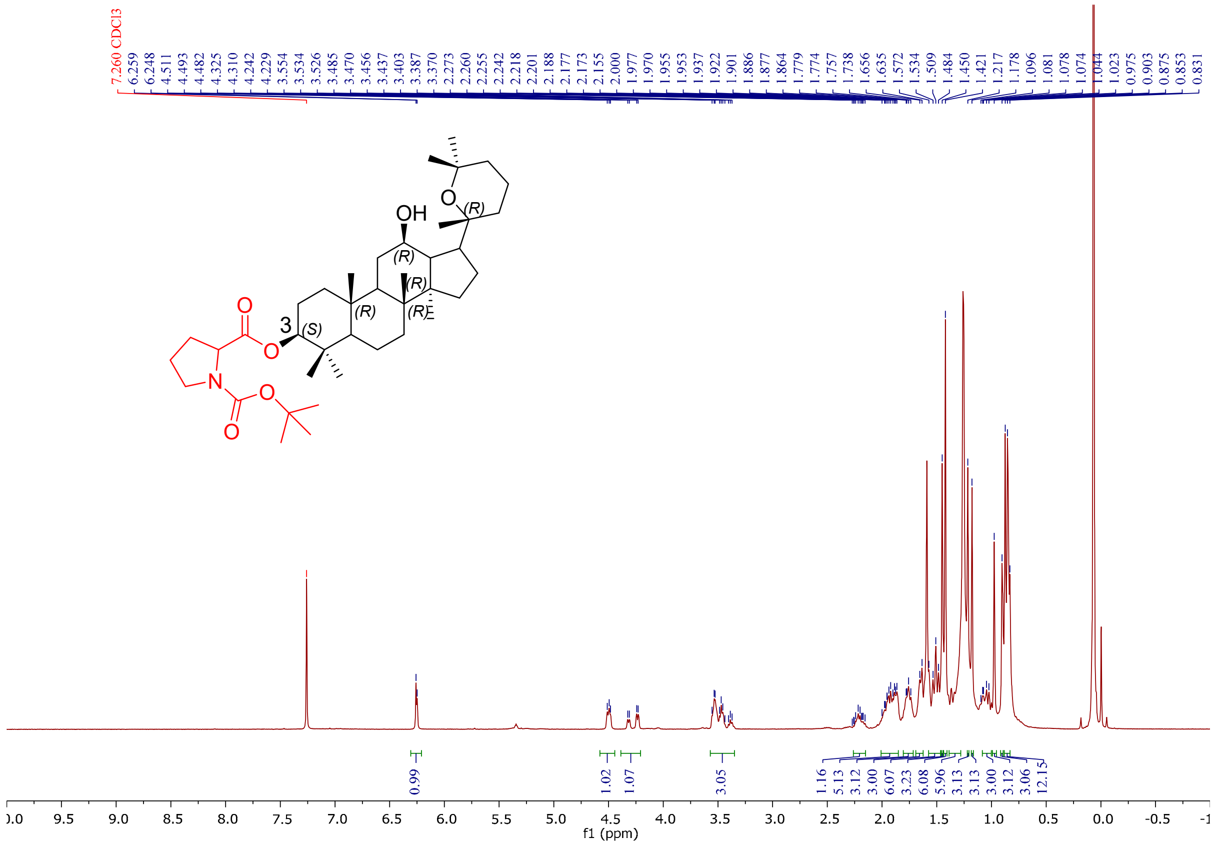

Figure S36. ^{13}C NMR Spectrum of compound **6** (126 MHz, CDCl$_3$)

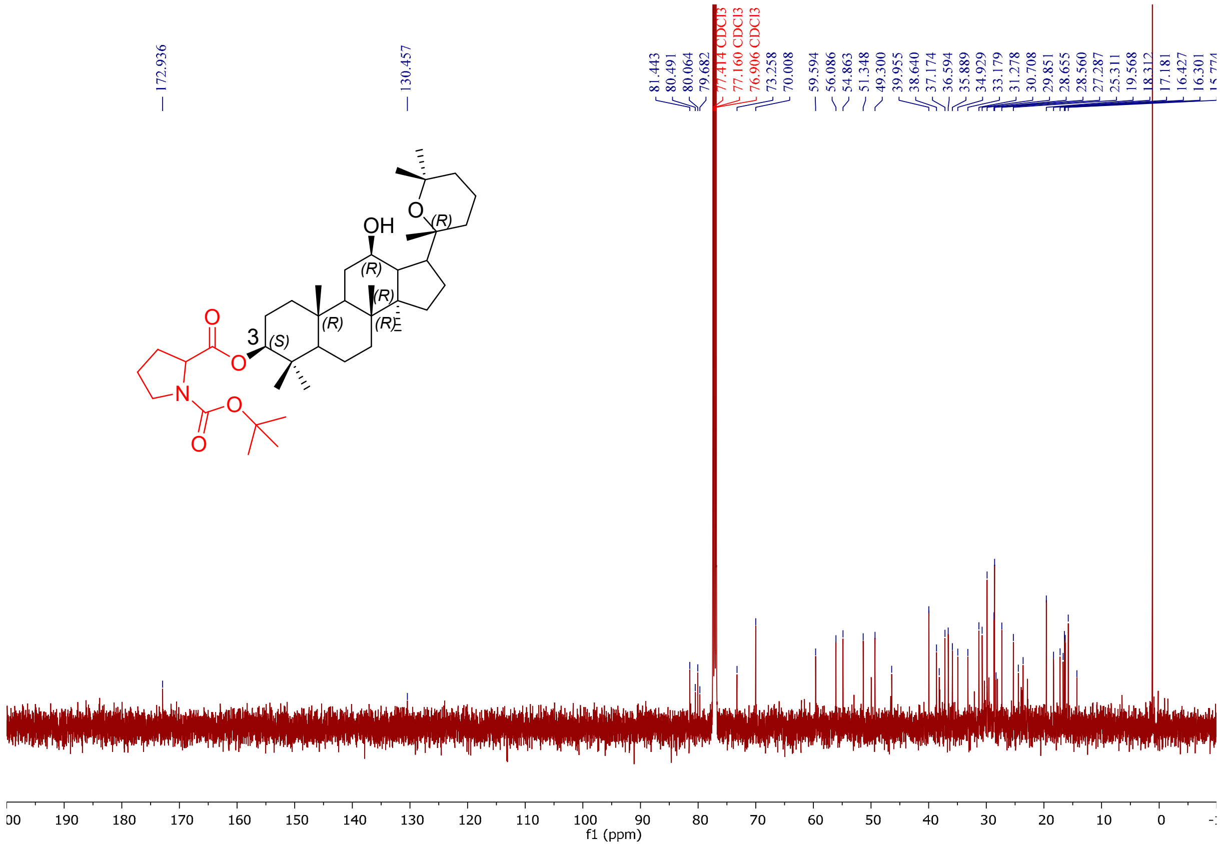

Figure S37. ^{1}H NMR Spectrum of compound **7** (500 MHz, $CDCl_3$)

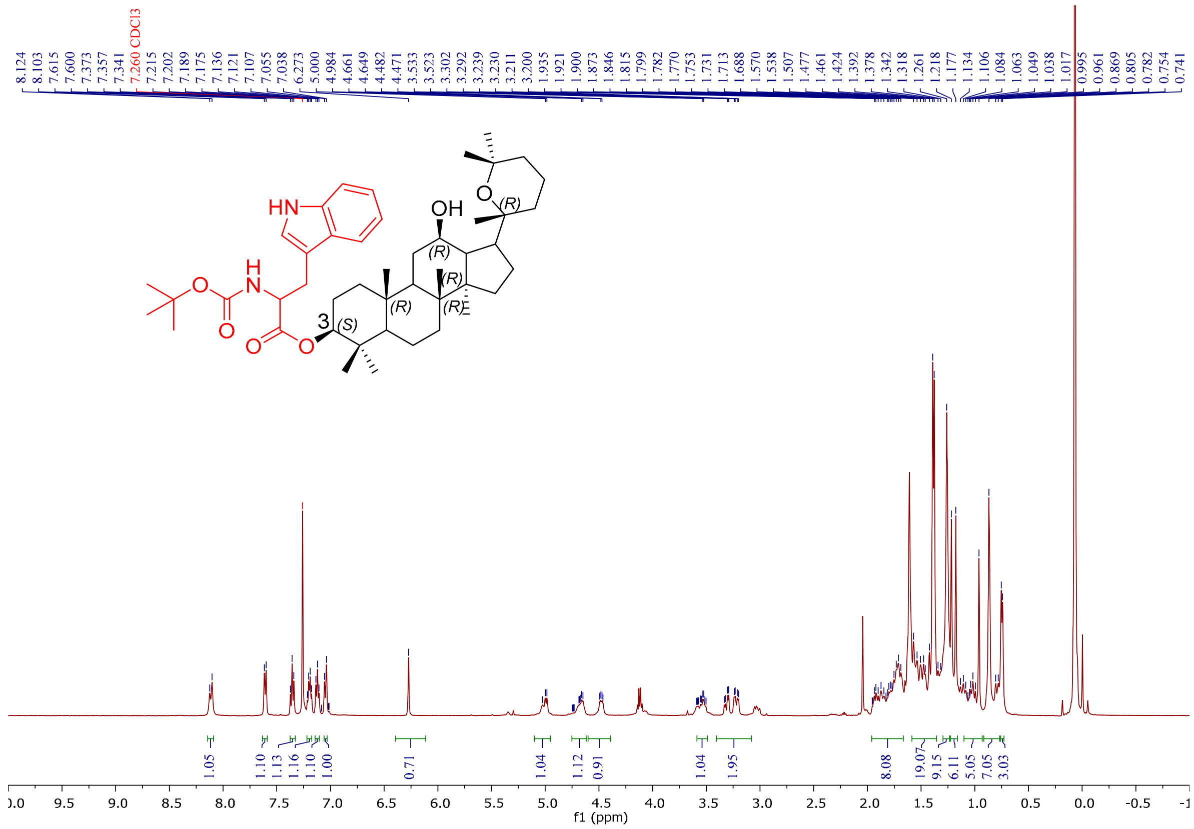

Figure S38. ^{13}C NMR Spectrum of compound **7** (126 MHz, $CDCl_3$)

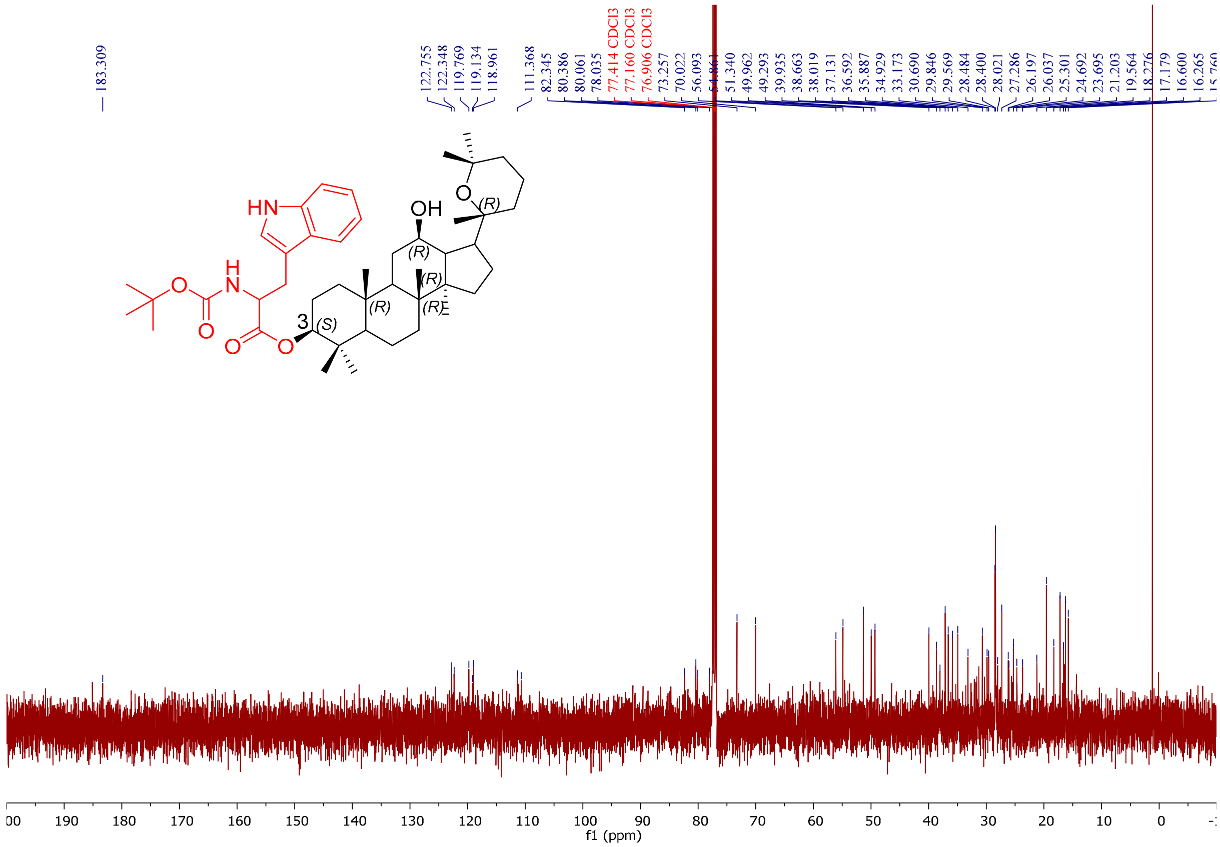

Figure S39. ^{1}H NMR Spectrum of compound **8** (500 MHz, $CDCl_3$)

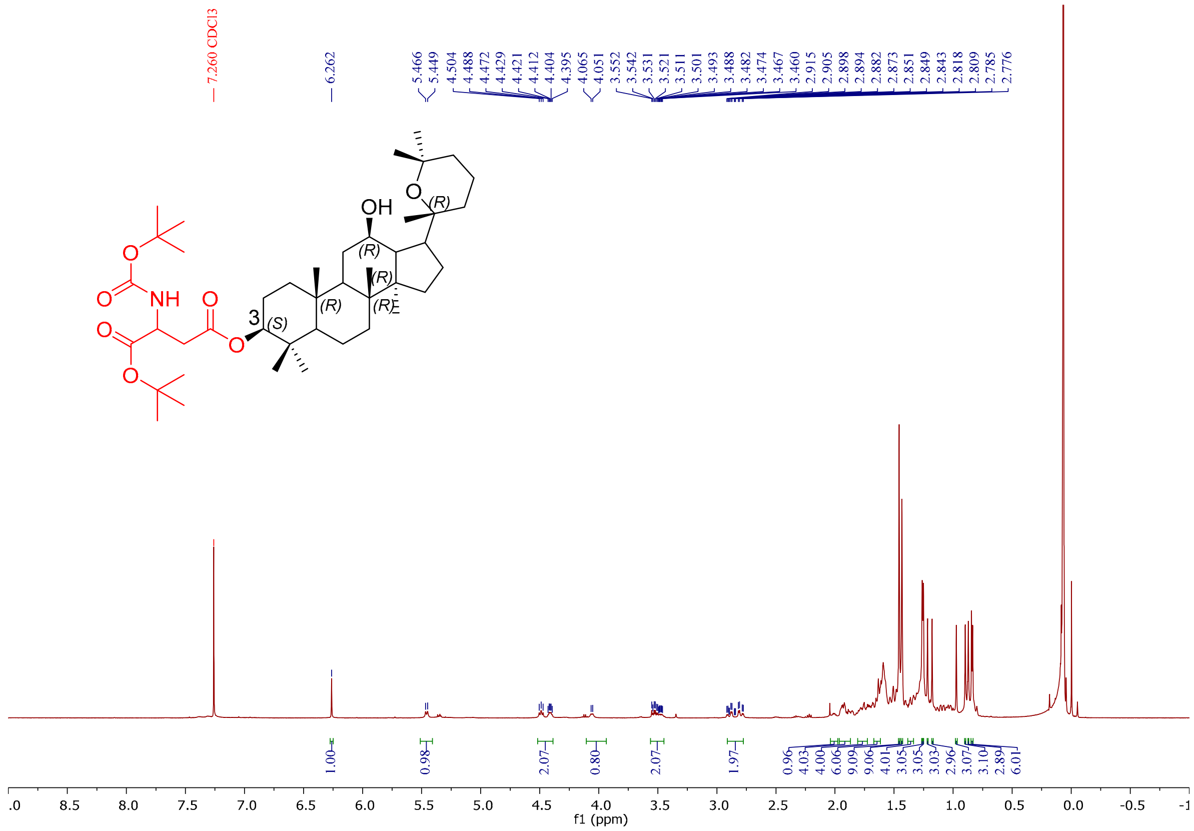

Figure S40. ^{13}C NMR Spectrum of compound **8** (126 MHz, $CDCl_3$)

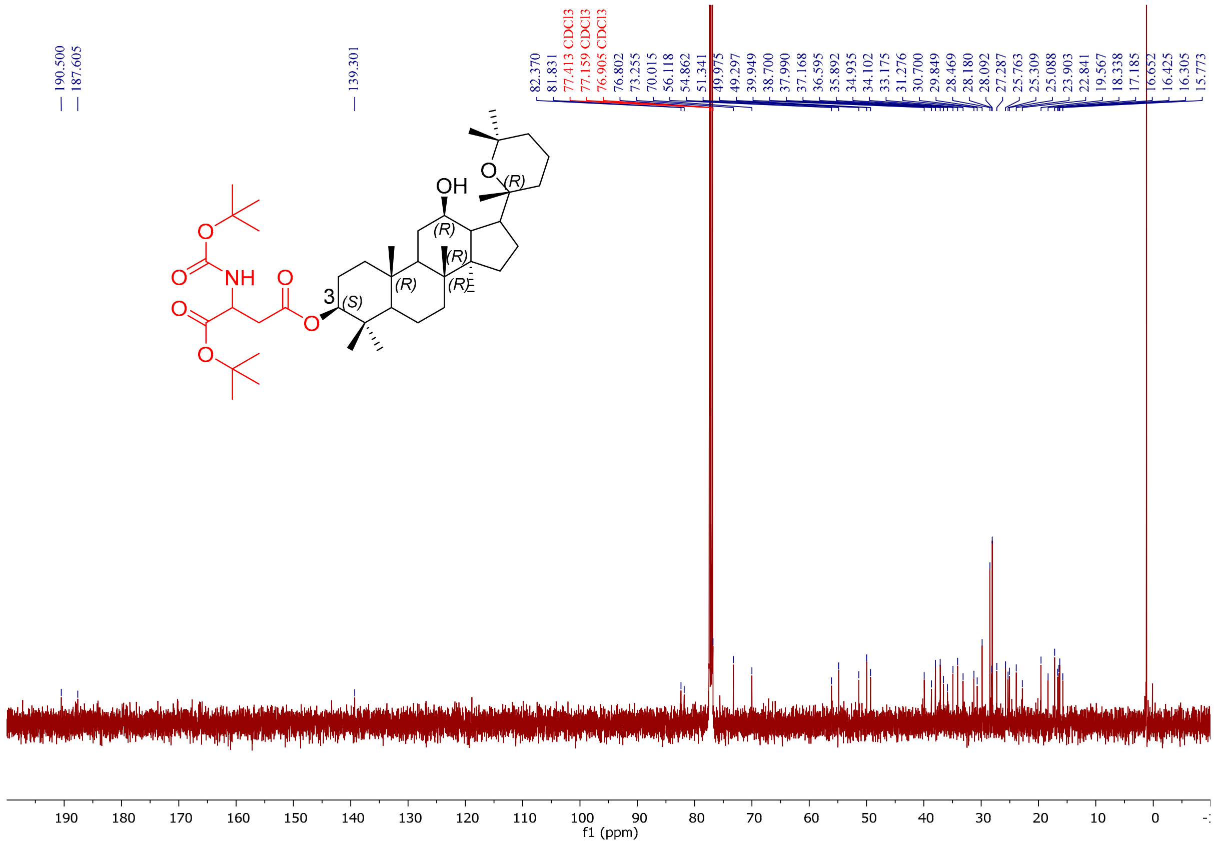

Figure S41. ^{1}H NMR Spectrum of compound **9** (500 MHz, $CDCl_3$)

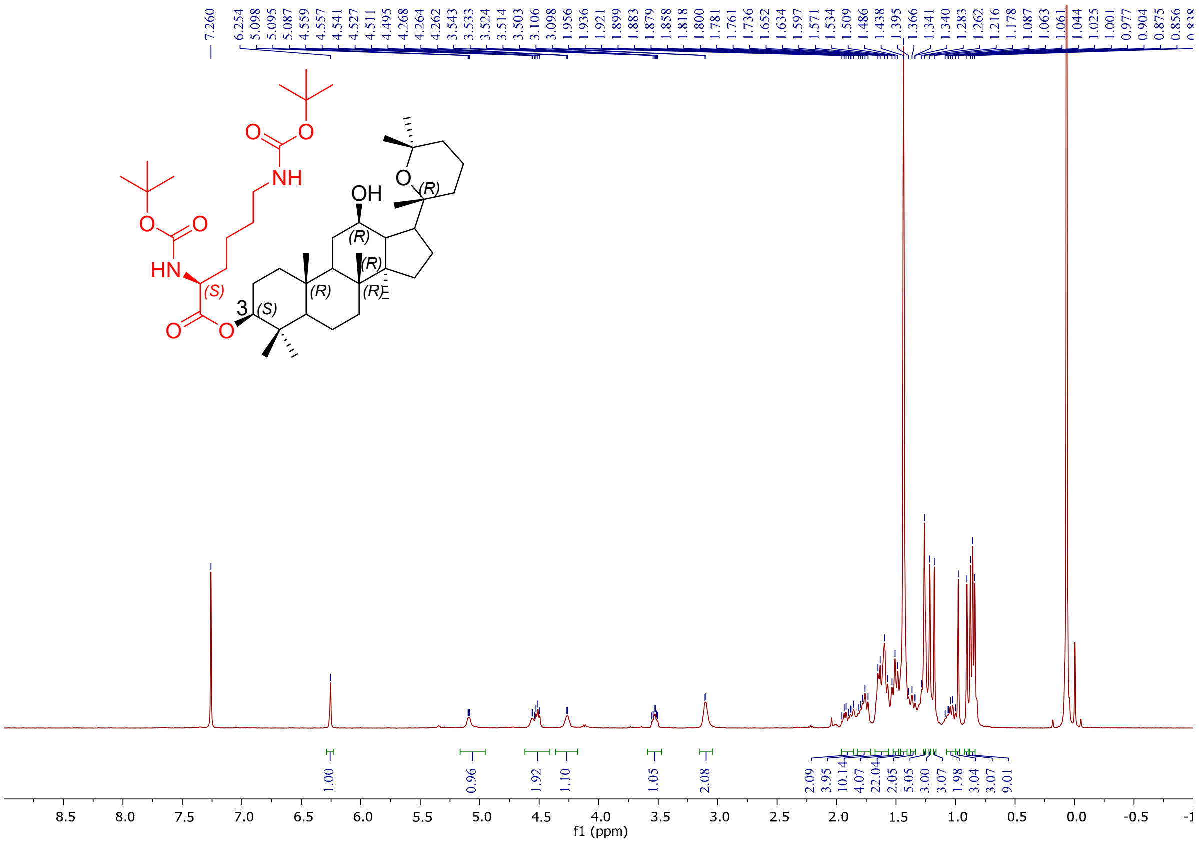

Figure S42. ^{13}C NMR Spectrum of compound **9** (126 MHz, $CDCl_3$)

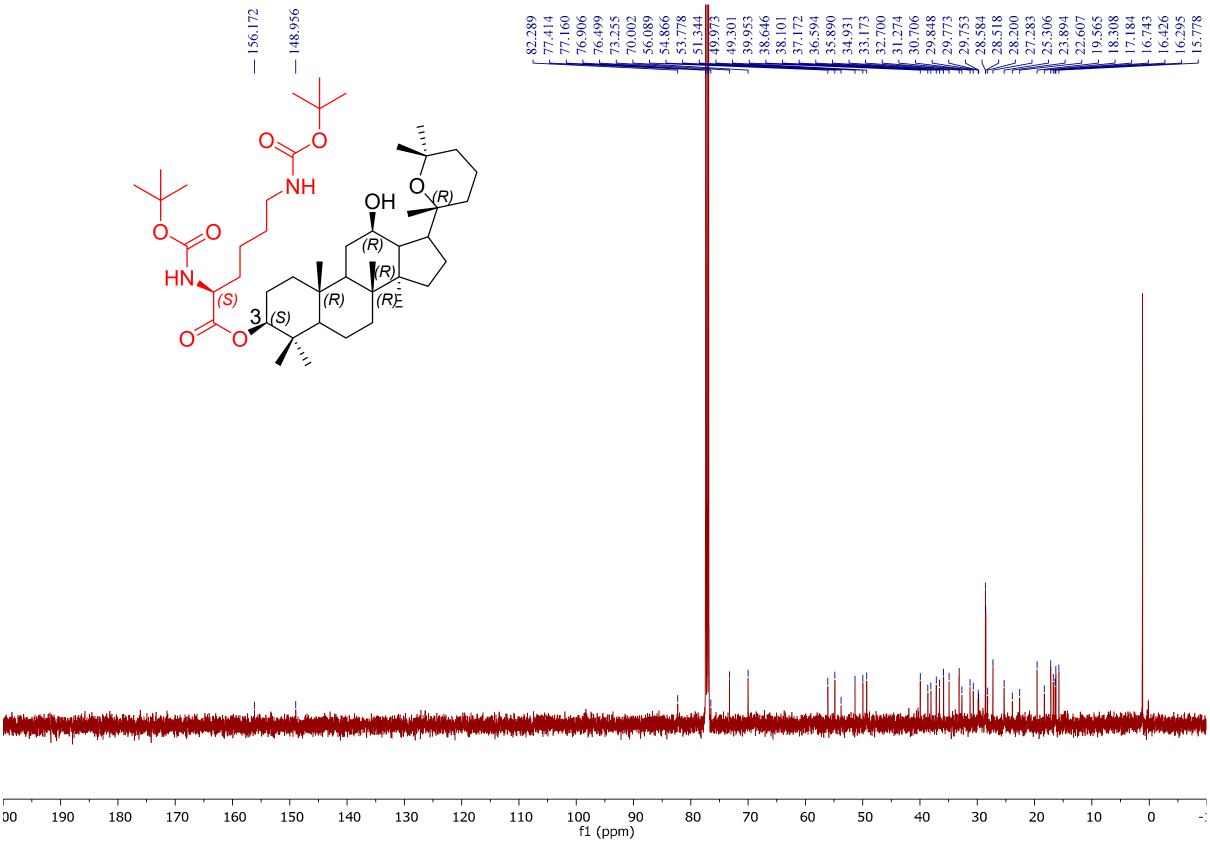

Figure S43. ^{1}H NMR Spectrum of compound **10** (500 MHz, $CDCl_3$)

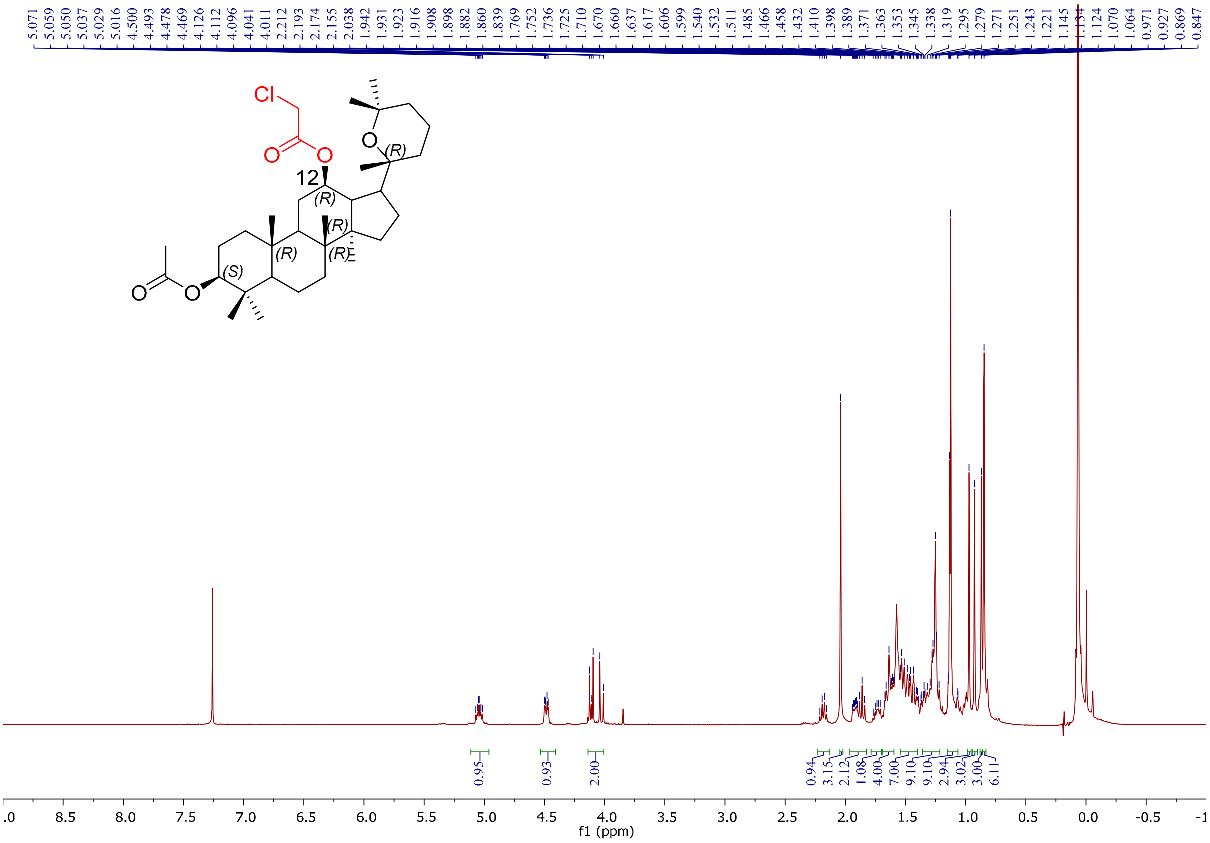

Figure S44. ^{13}C NMR Spectrum of compound **10** (126 MHz, $CDCl_3$)

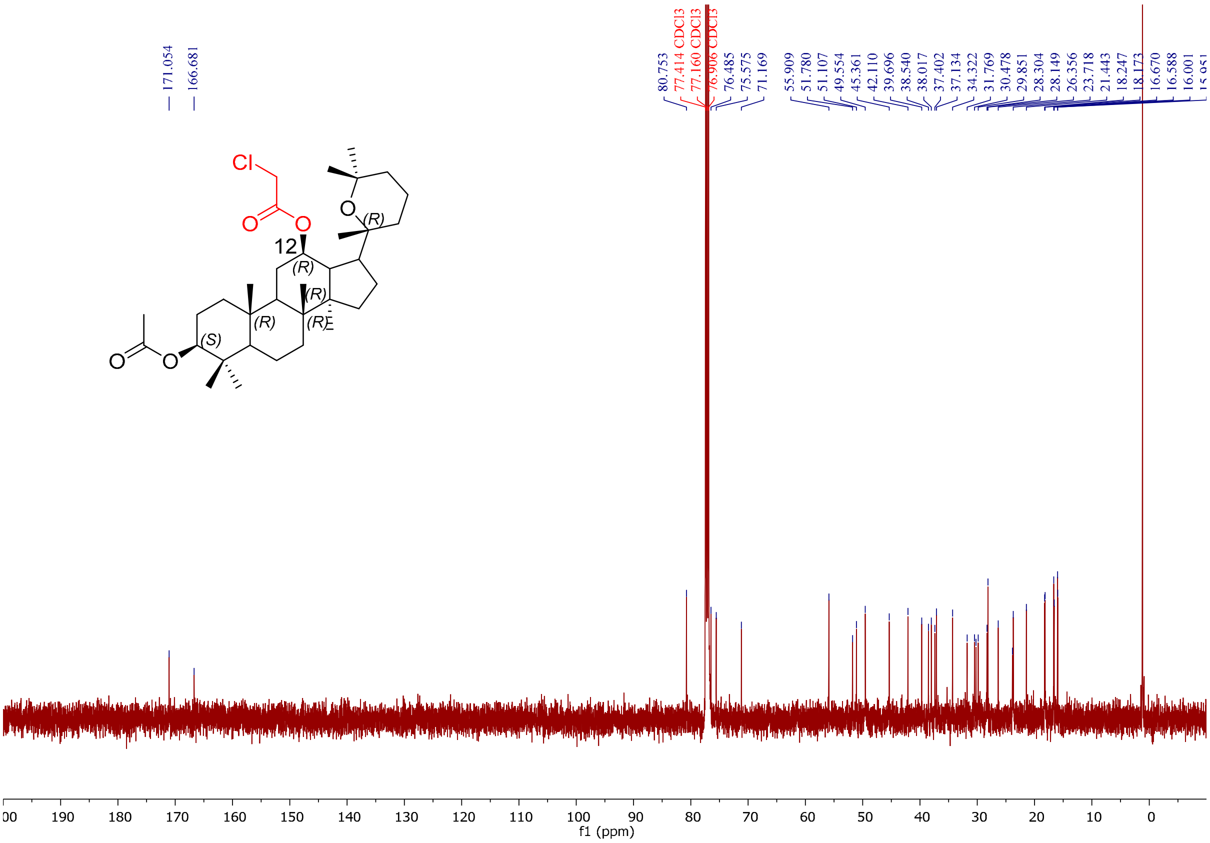

Figure S45. ^{1}H NMR Spectrum of compound **11** (500 MHz, $CDCl_3$)

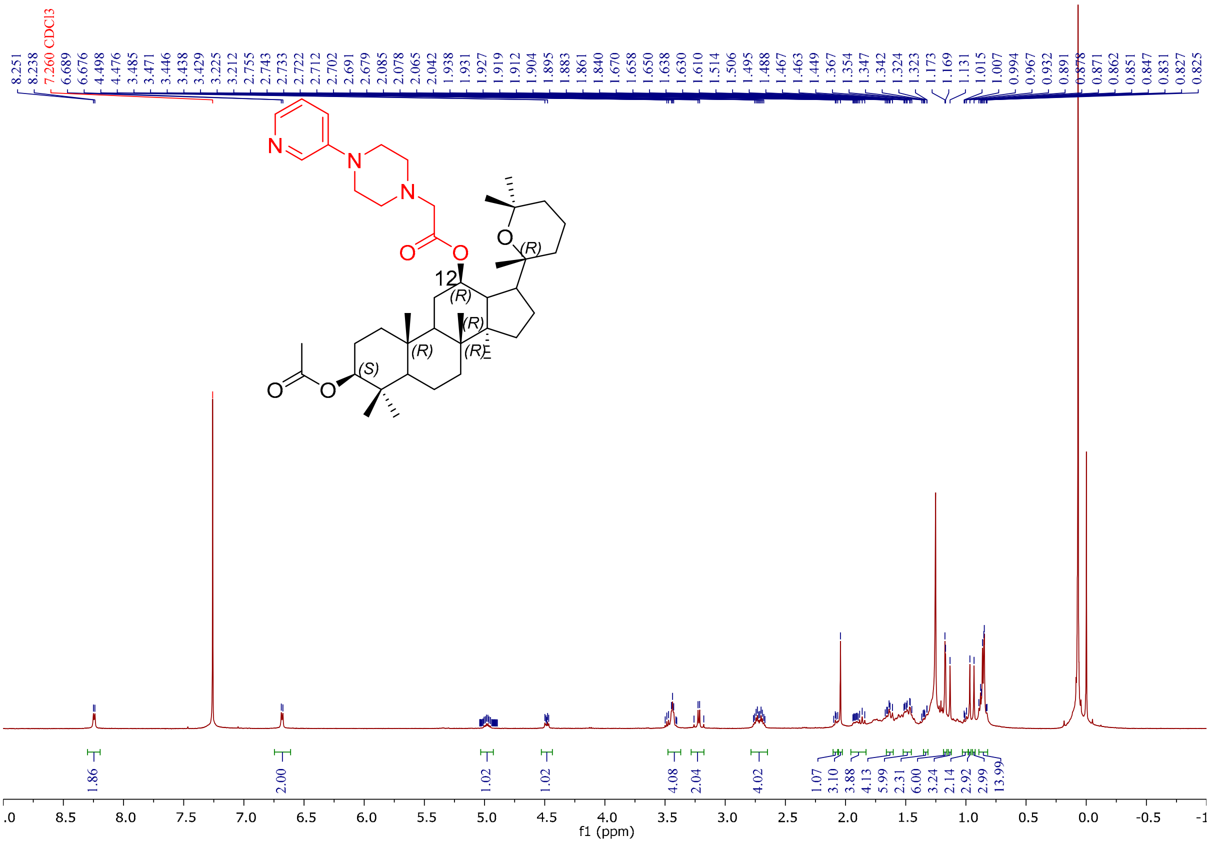

Figure S46. ^{13}C NMR Spectrum of compound **11** (126 MHz, $CDCl_3$)

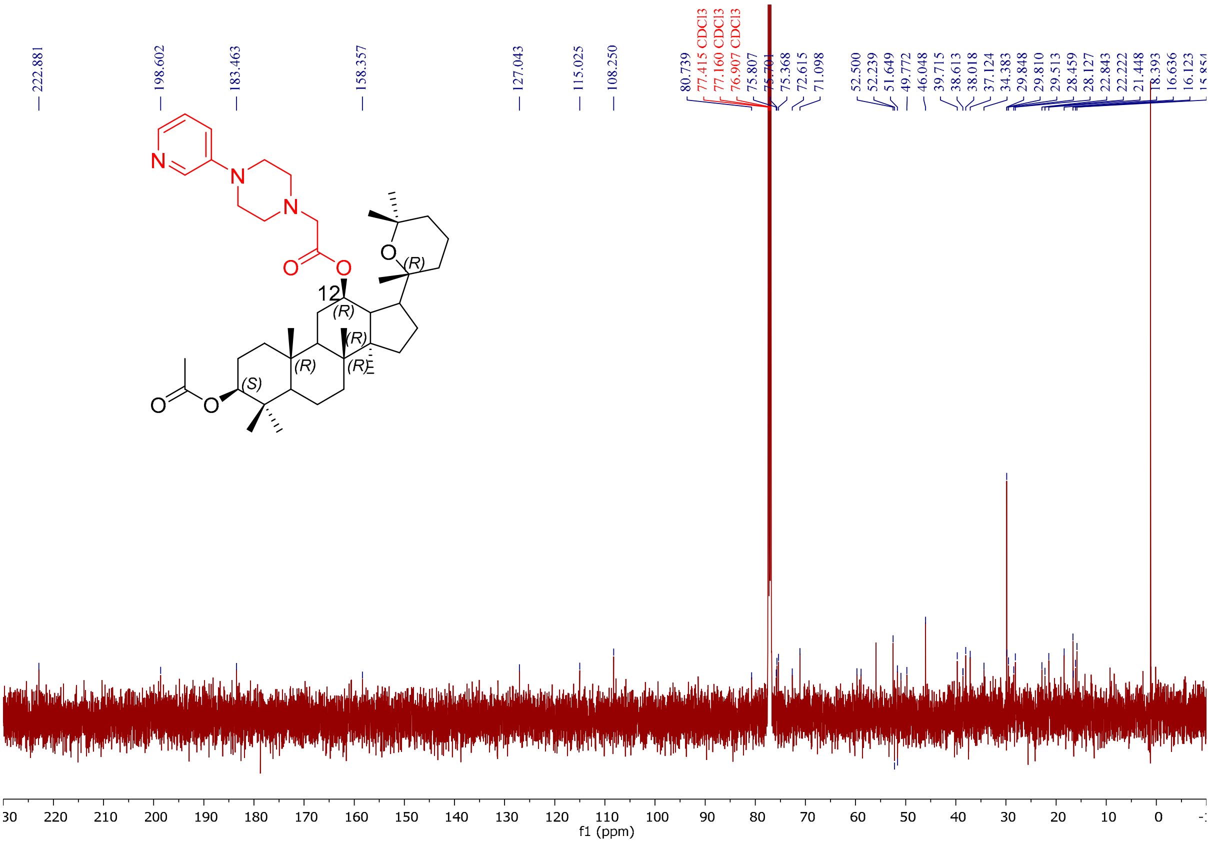

Figure S47. ^{1}H NMR Spectrum of compound **12** (500 MHz, $CDCl_3$)

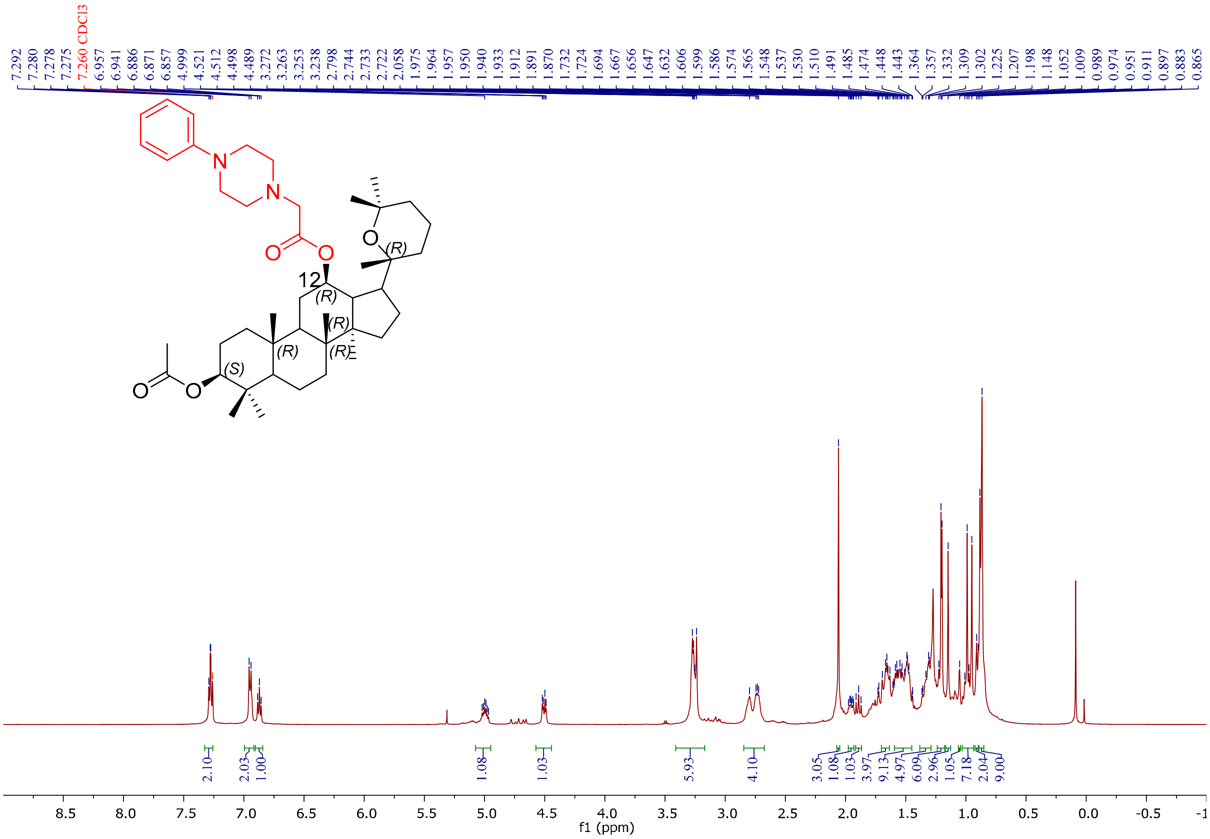

Figure S48. ^{13}C NMR Spectrum of compound **12** (126 MHz, $CDCl_3$)

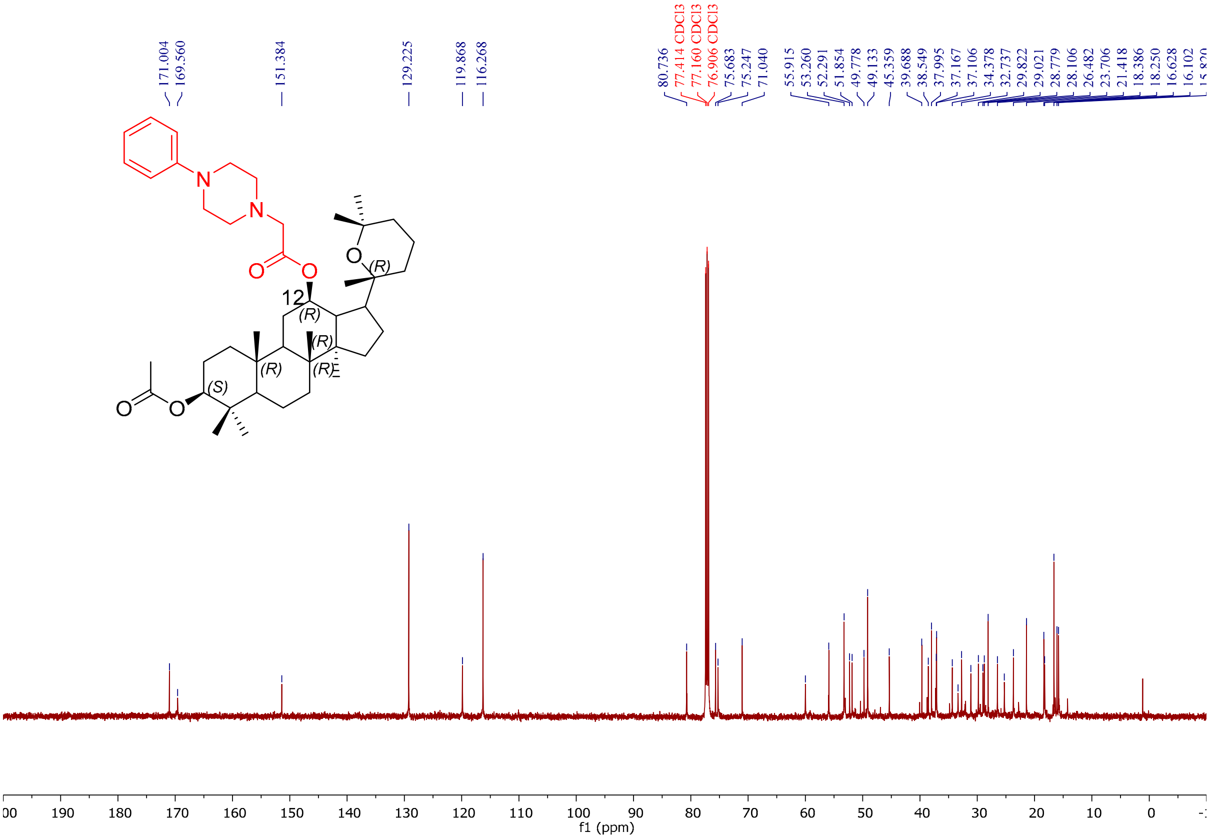

Figure S49. ^{1}H NMR Spectrum of compound **13** (500 MHz, $CDCl_3$)

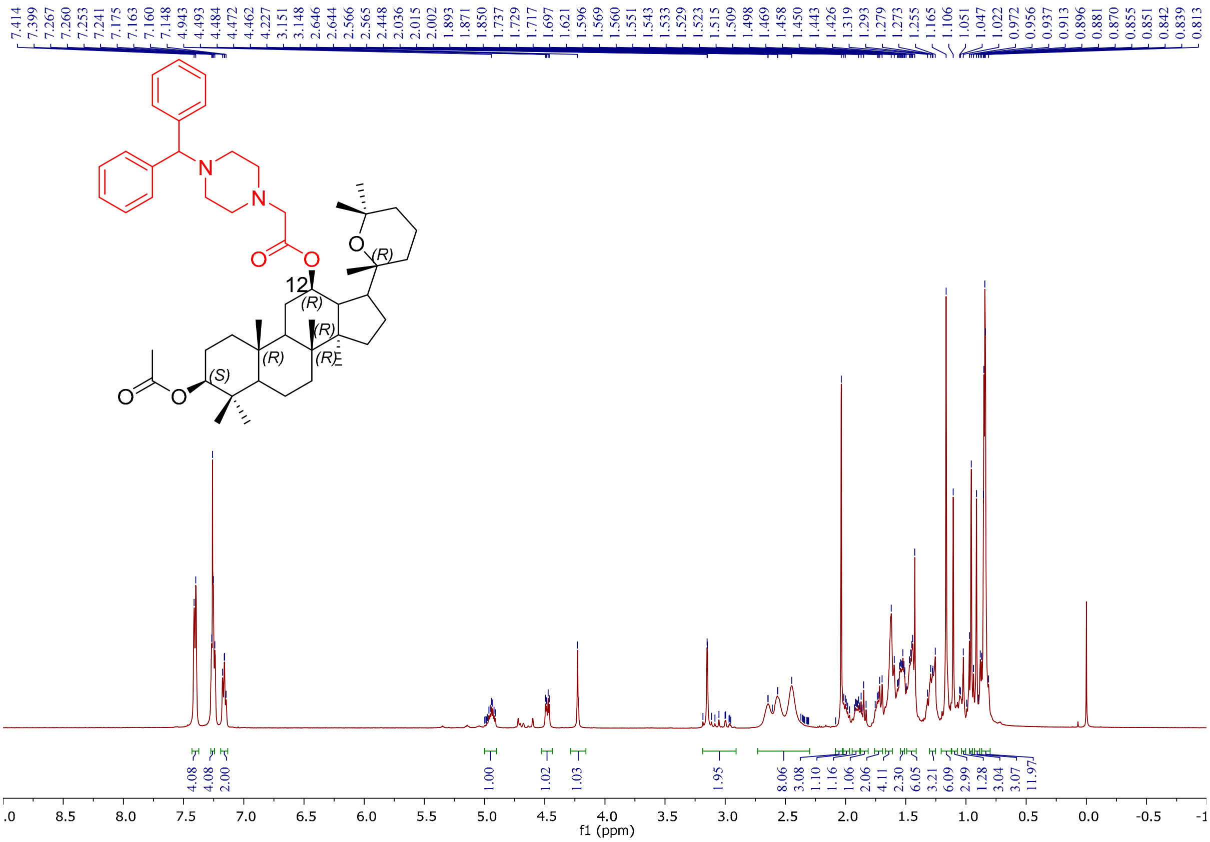

Figure S50. ^{13}C NMR Spectrum of compound **13** (126 MHz, $CDCl_3$)

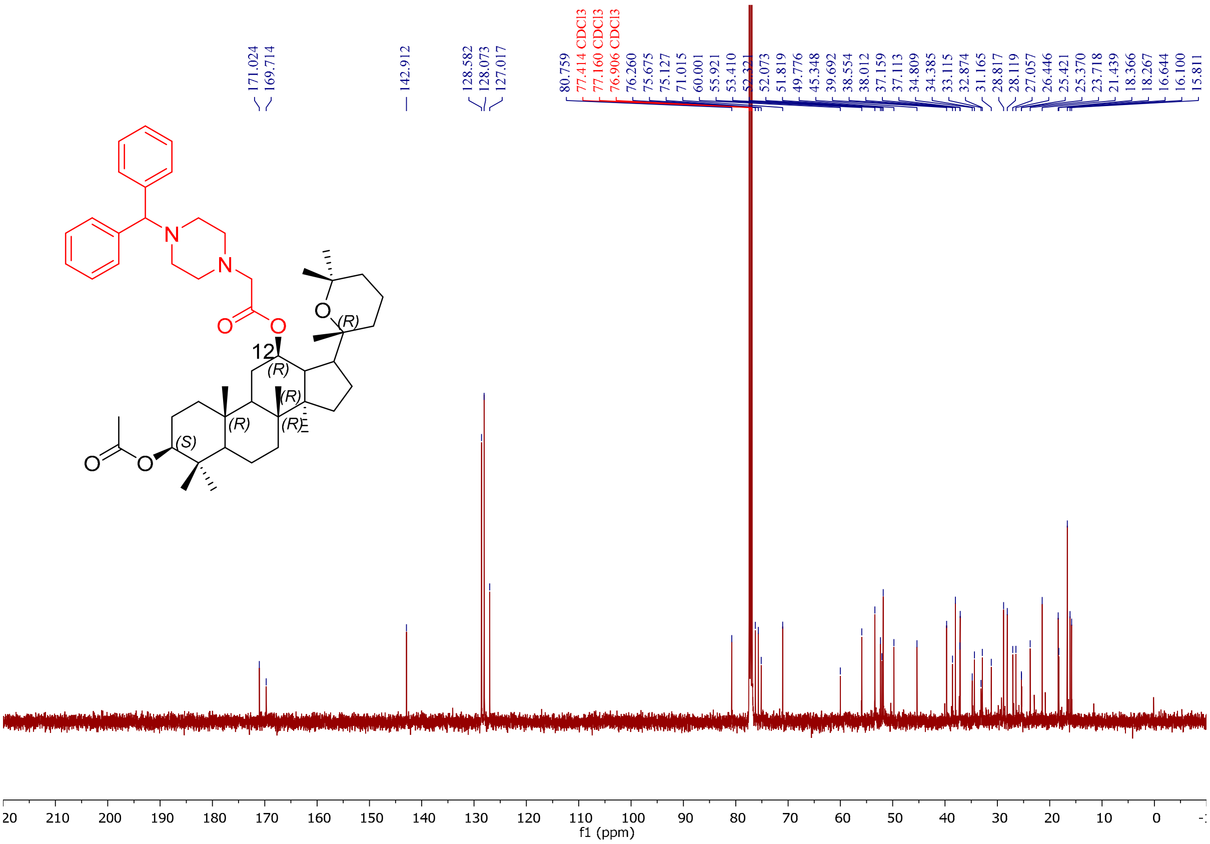

Figure S51. ^{1}H NMR Spectrum of compound **14** (500 MHz, $CDCl_3$)

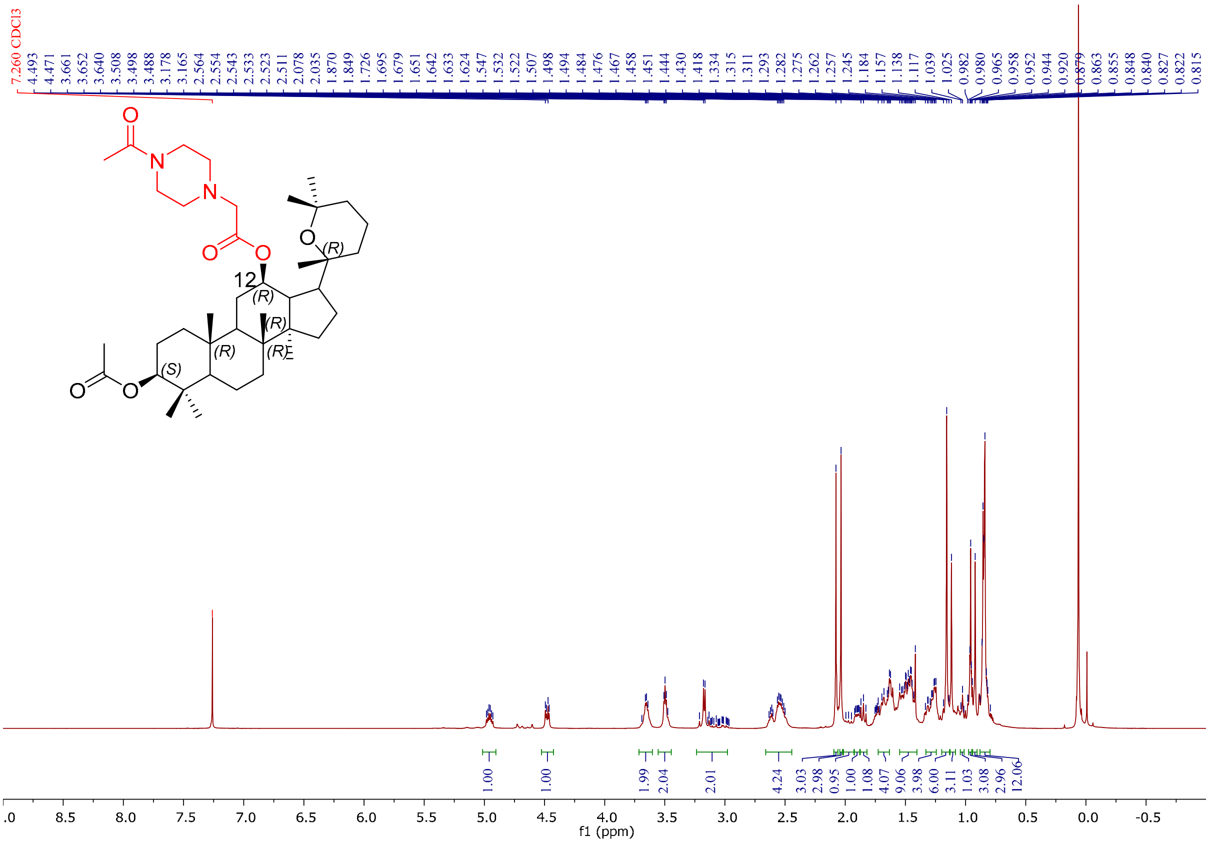

Figure S52. ^{13}C NMR Spectrum of compound **14** (126 MHz, $CDCl_3$)

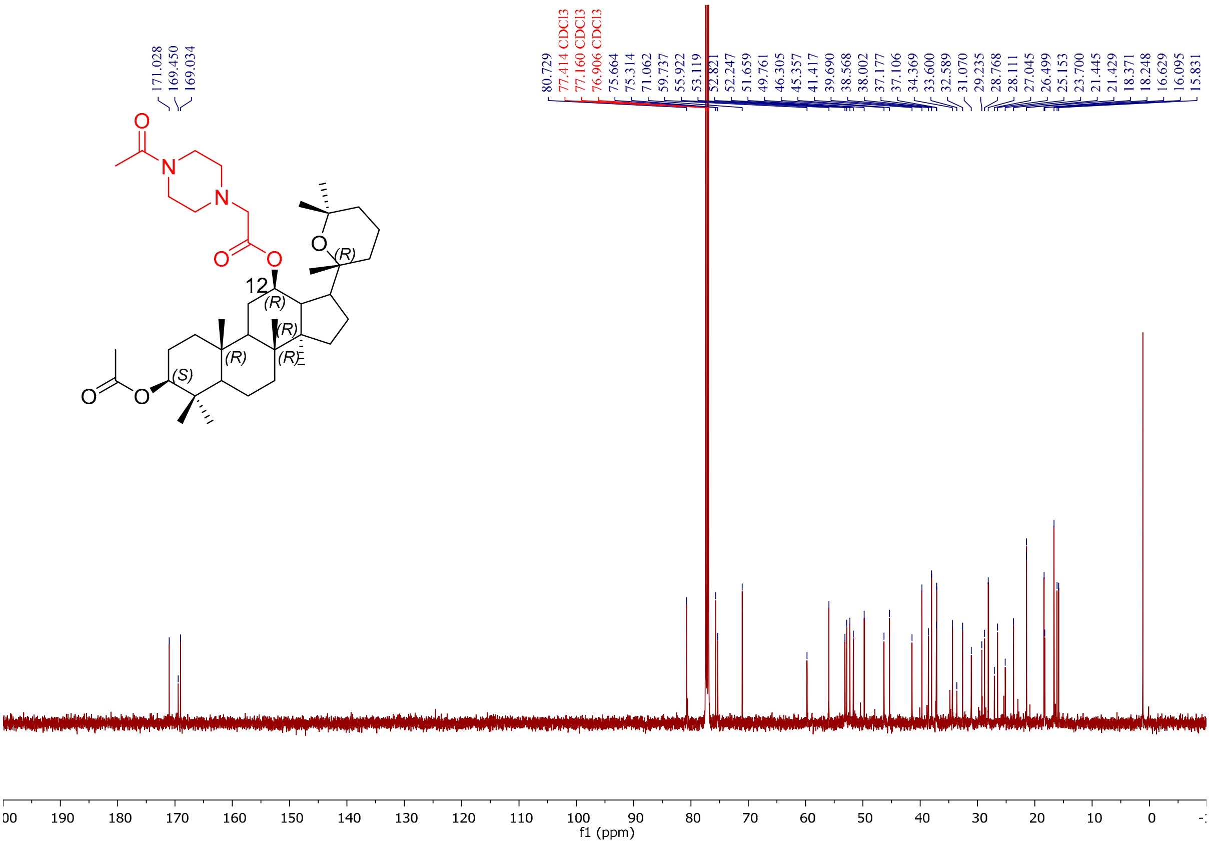

Figure S53. ^{1}H NMR Spectrum of compound **15** (500 MHz, $CDCl_3$)

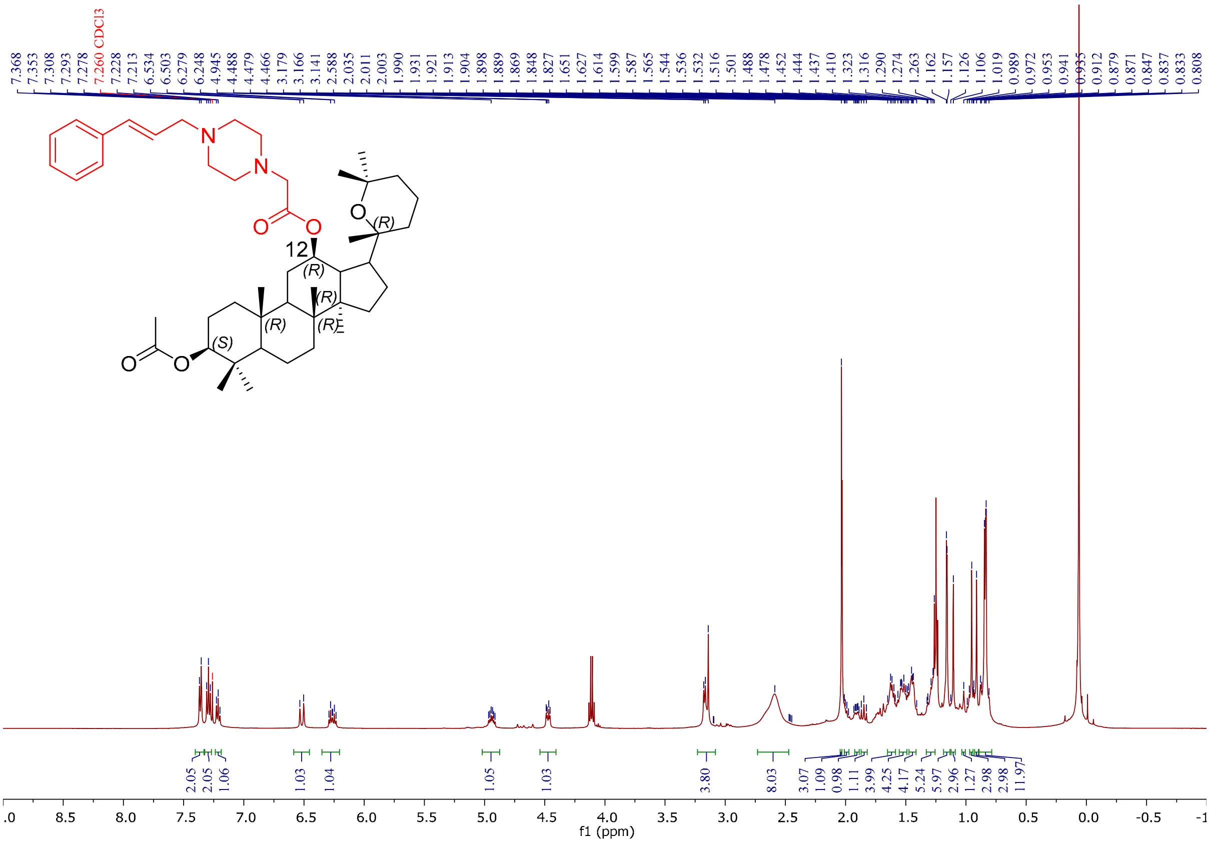

Figure S54. ^{13}C NMR Spectrum of compound **15** (126 MHz, $CDCl_3$)

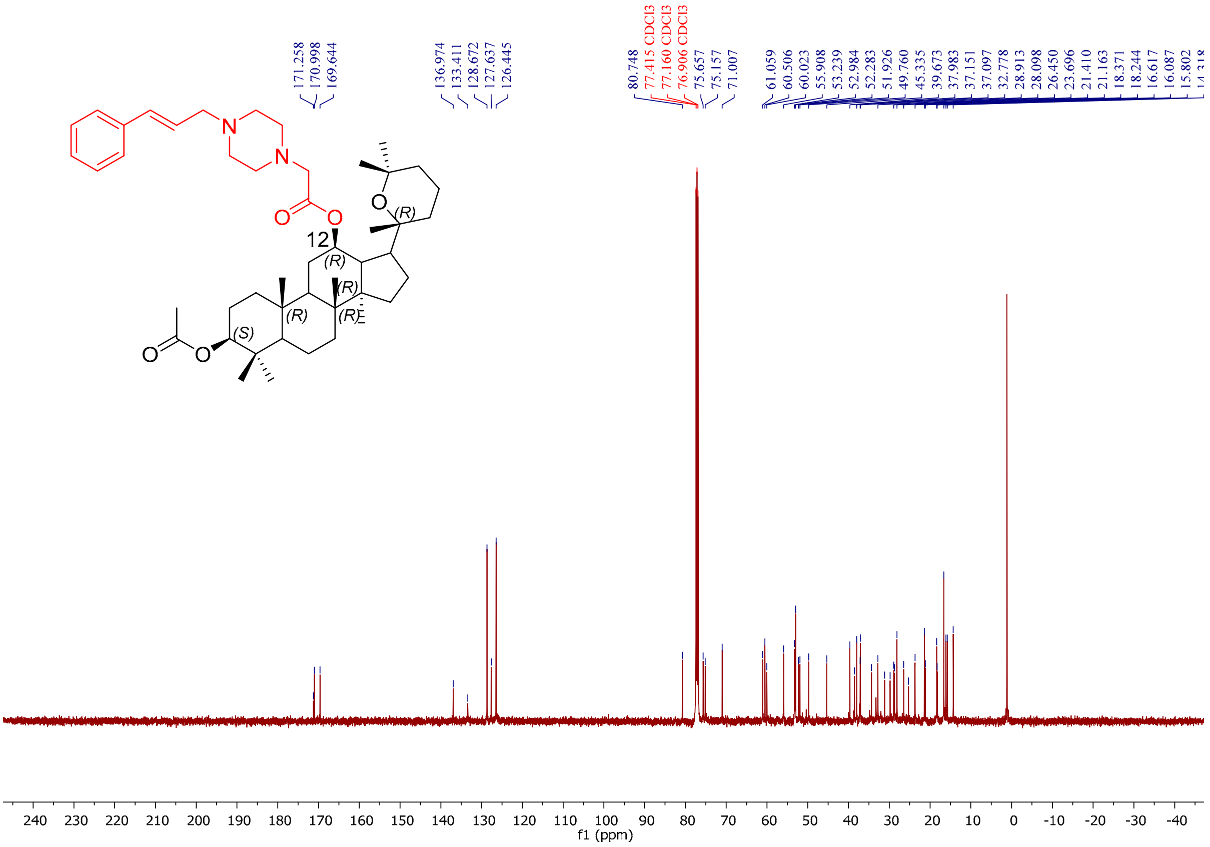

Figure S55. ^{1}H NMR Spectrum of compound **16** (500 MHz, $CDCl_3$)

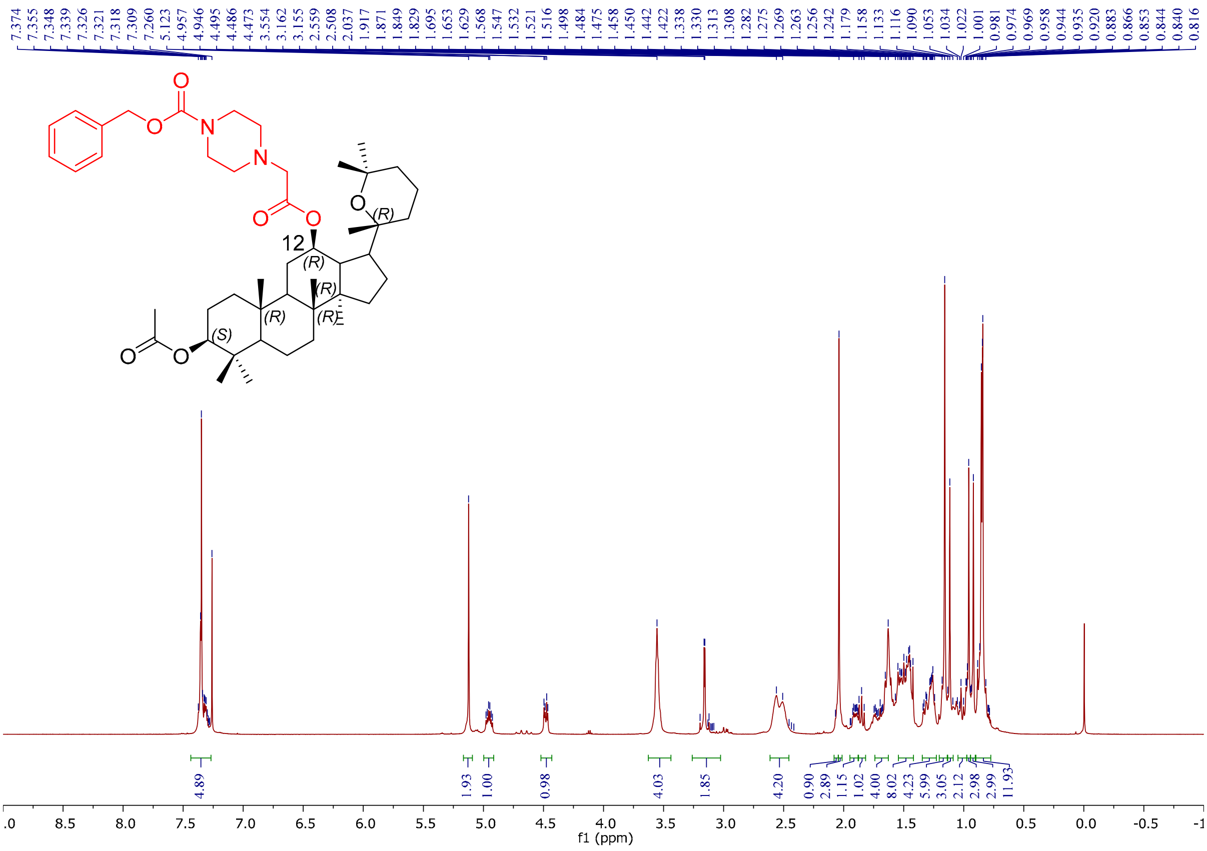

Figure S56. ^{13}C NMR Spectrum of compound **16** (126 MHz, $CDCl_3$)

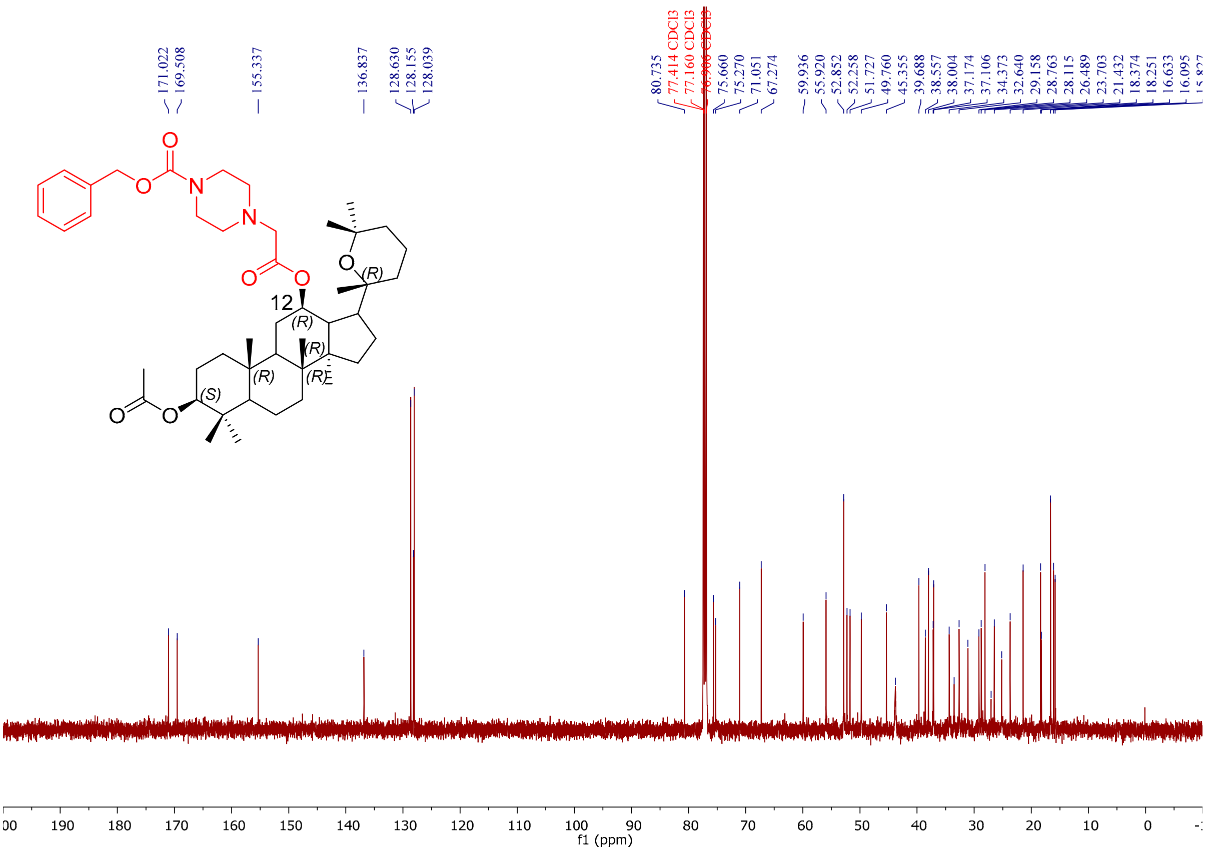

Figure S57. ^{1}H NMR Spectrum of compound **17** (500 MHz, $CDCl_3$)

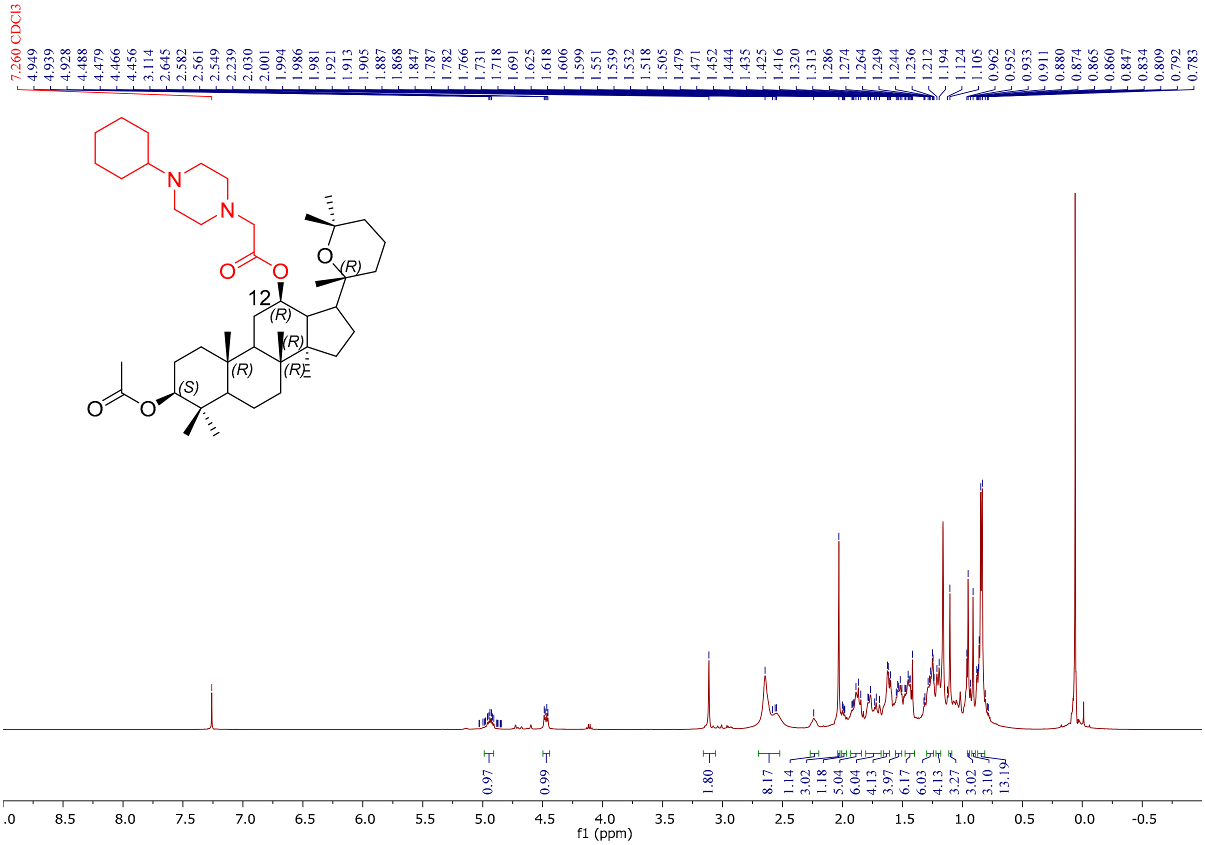

Figure S58. ^{13}C NMR Spectrum of compound **17** (126 MHz, $CDCl_3$)

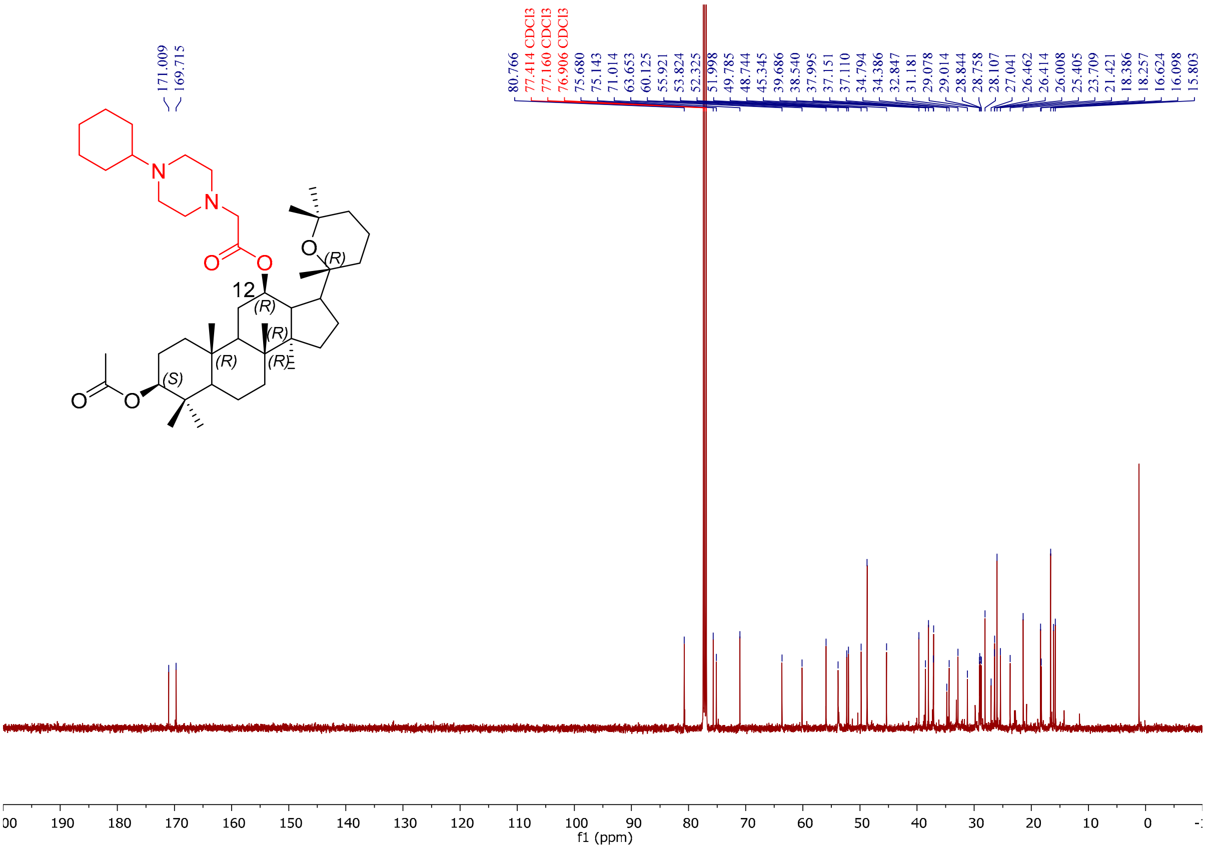

Figure S59. ^{1}H NMR Spectrum of compound **18** (500 MHz, CDCl$_3$)

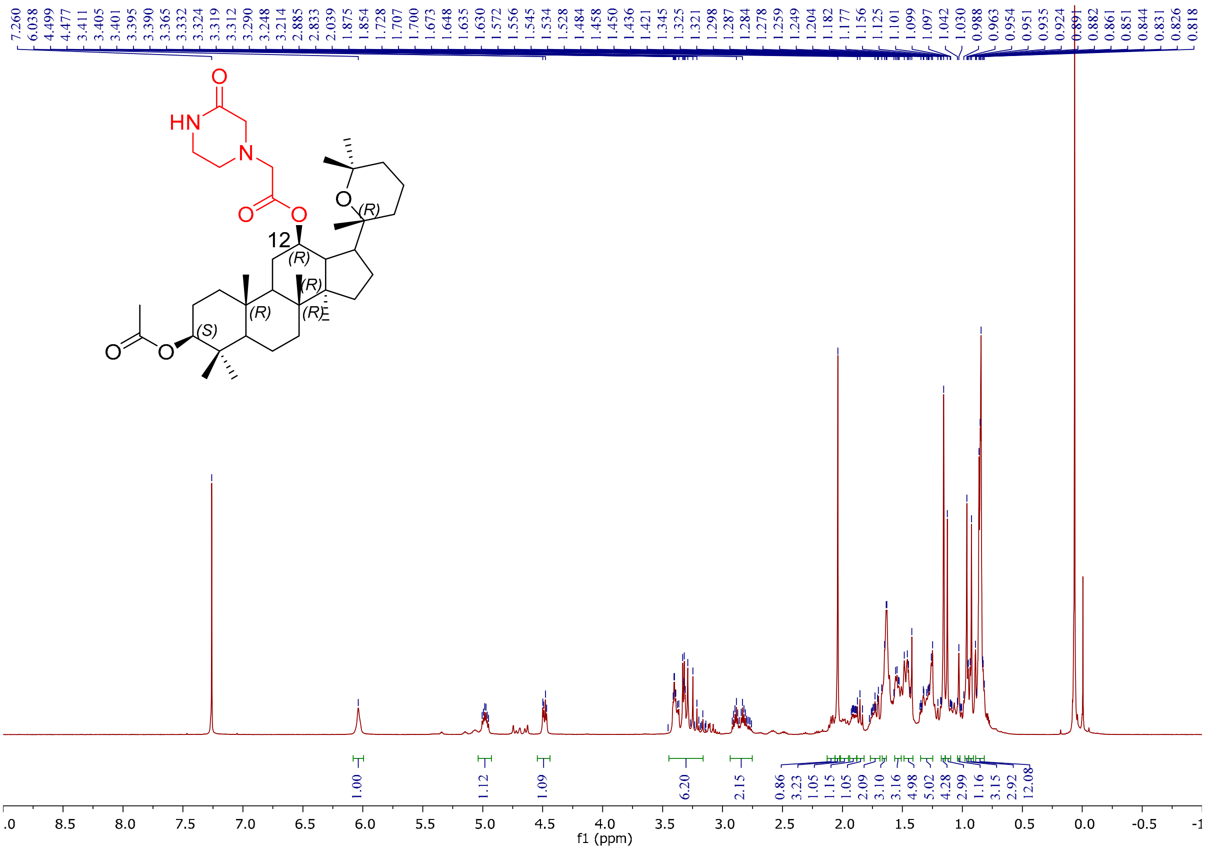

Figure S60. ^{13}C NMR Spectrum of compound **18** (126 MHz, CDCl$_3$)

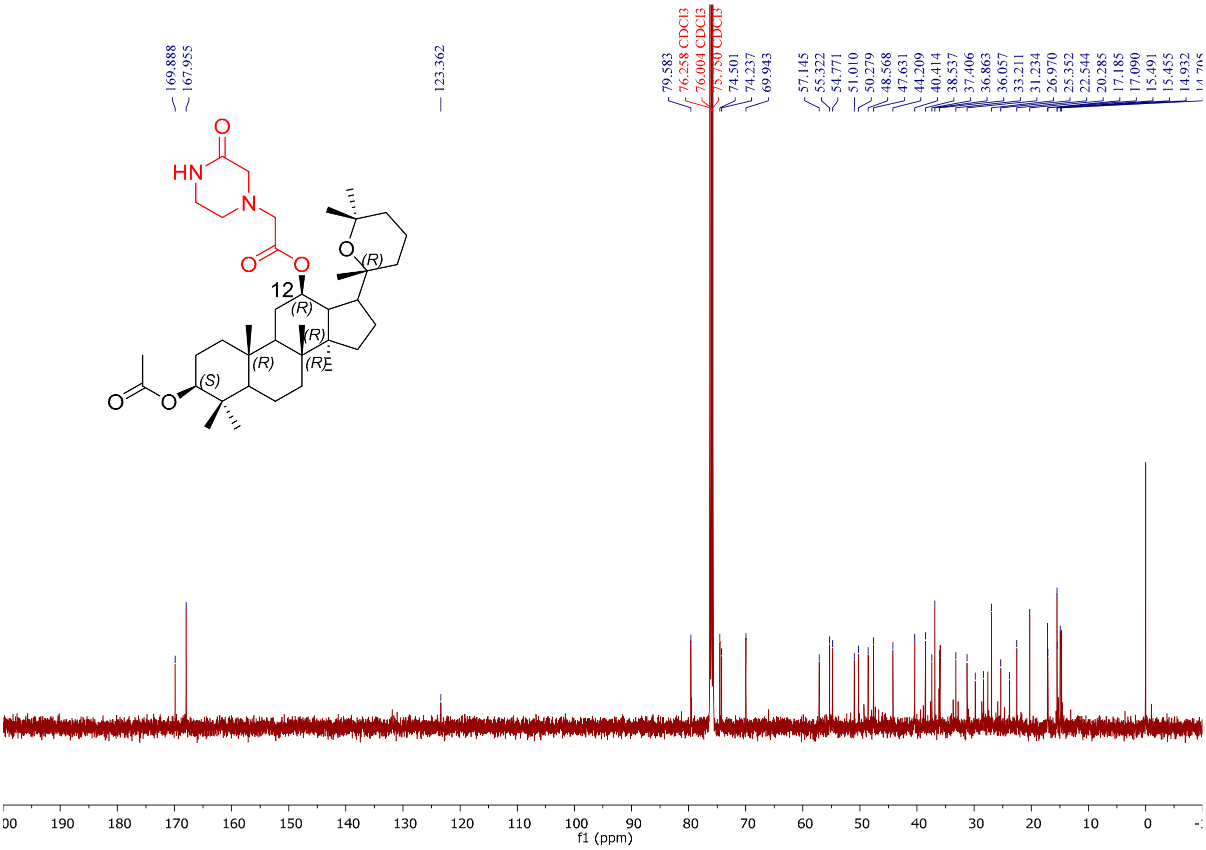

Figure S61. ^{1}H NMR Spectrum of compound **19** (500 MHz, $CDCl_3$)

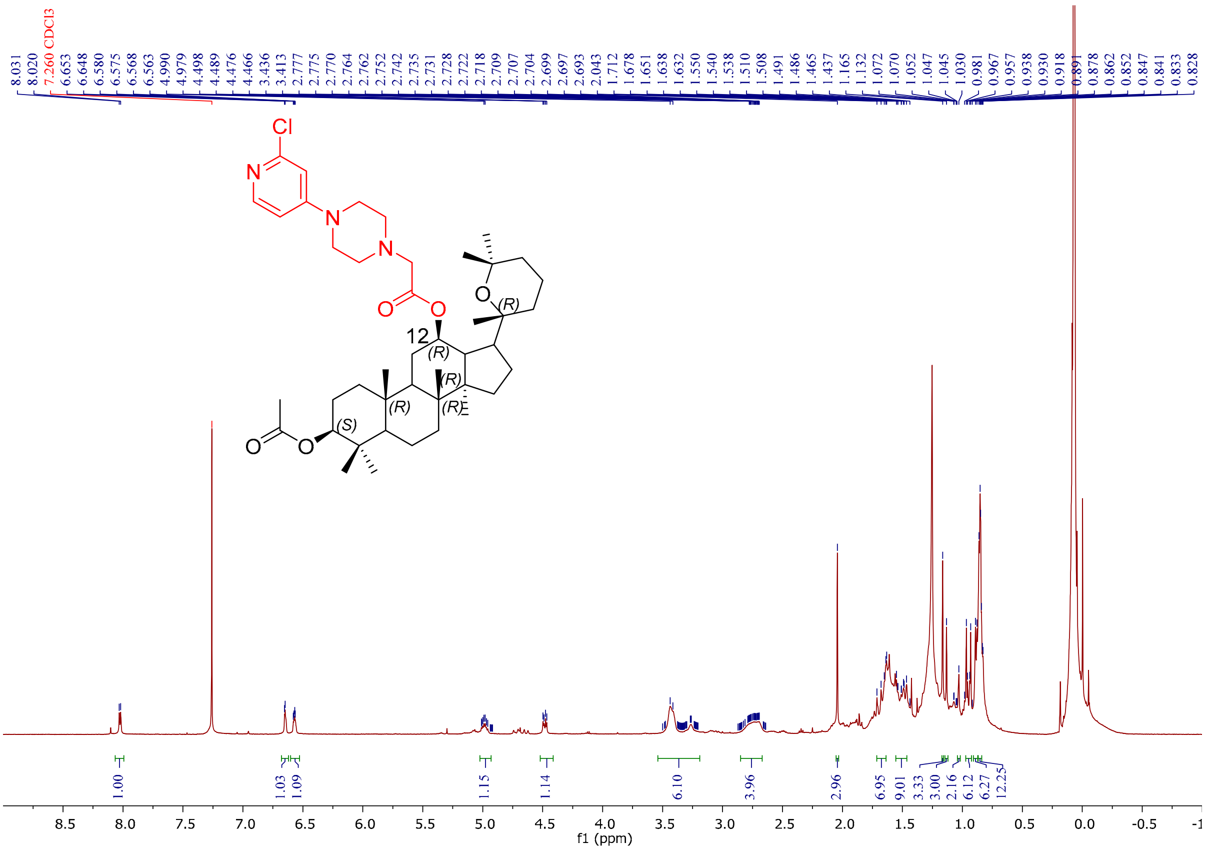

Figure S62. ^{13}C NMR Spectrum of compound **19** (126 MHz, $CDCl_3$)

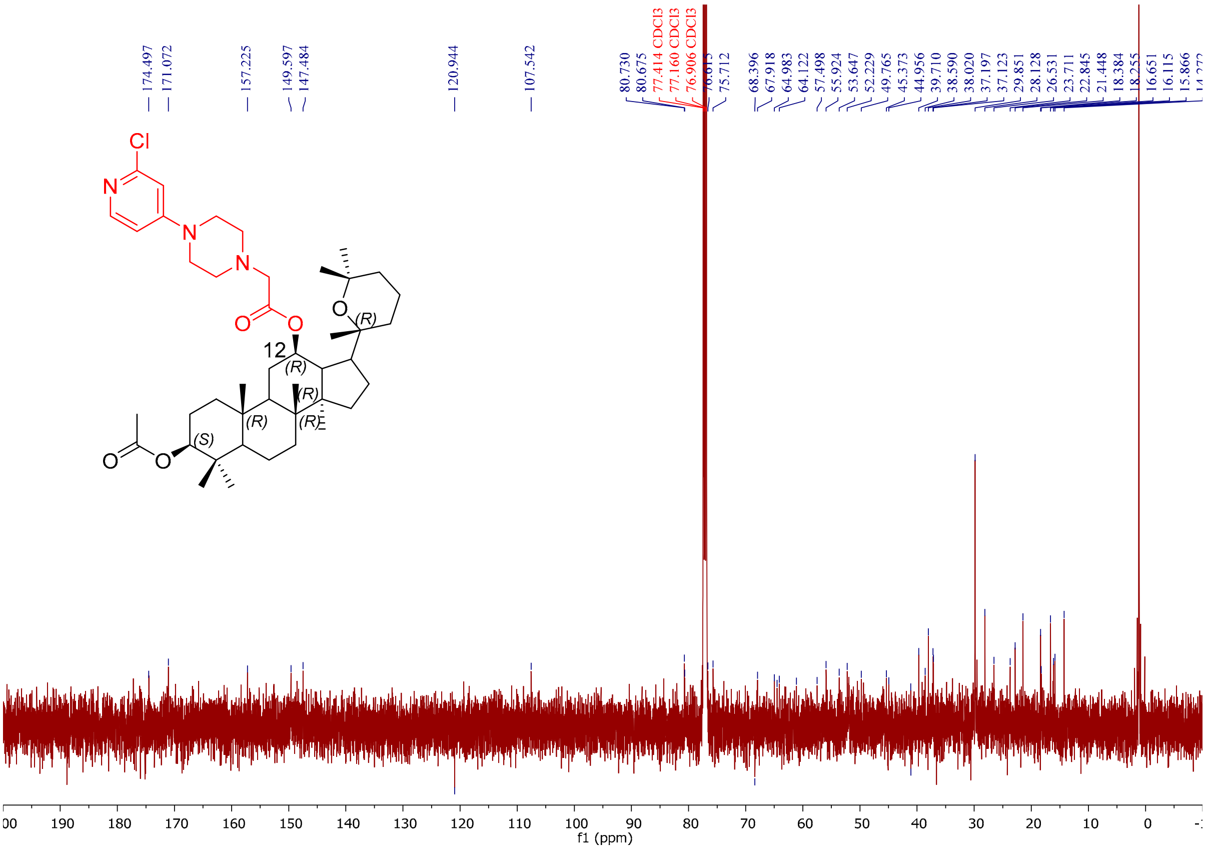

Figure S63. ^{1}H NMR Spectrum of compound **20** (500 MHz, $CDCl_3$)

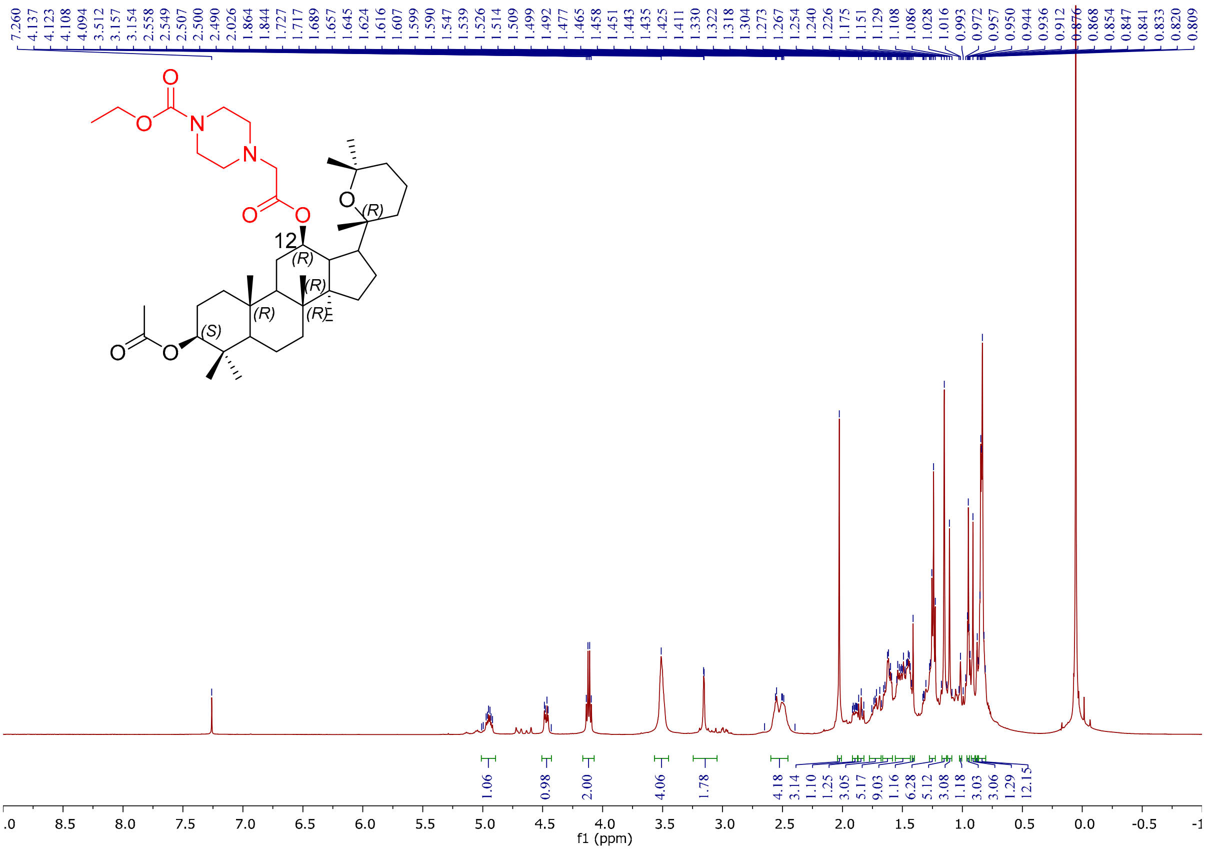

Figure S64. ^{13}C NMR Spectrum of compound **20** (126 MHz, $CDCl_3$)

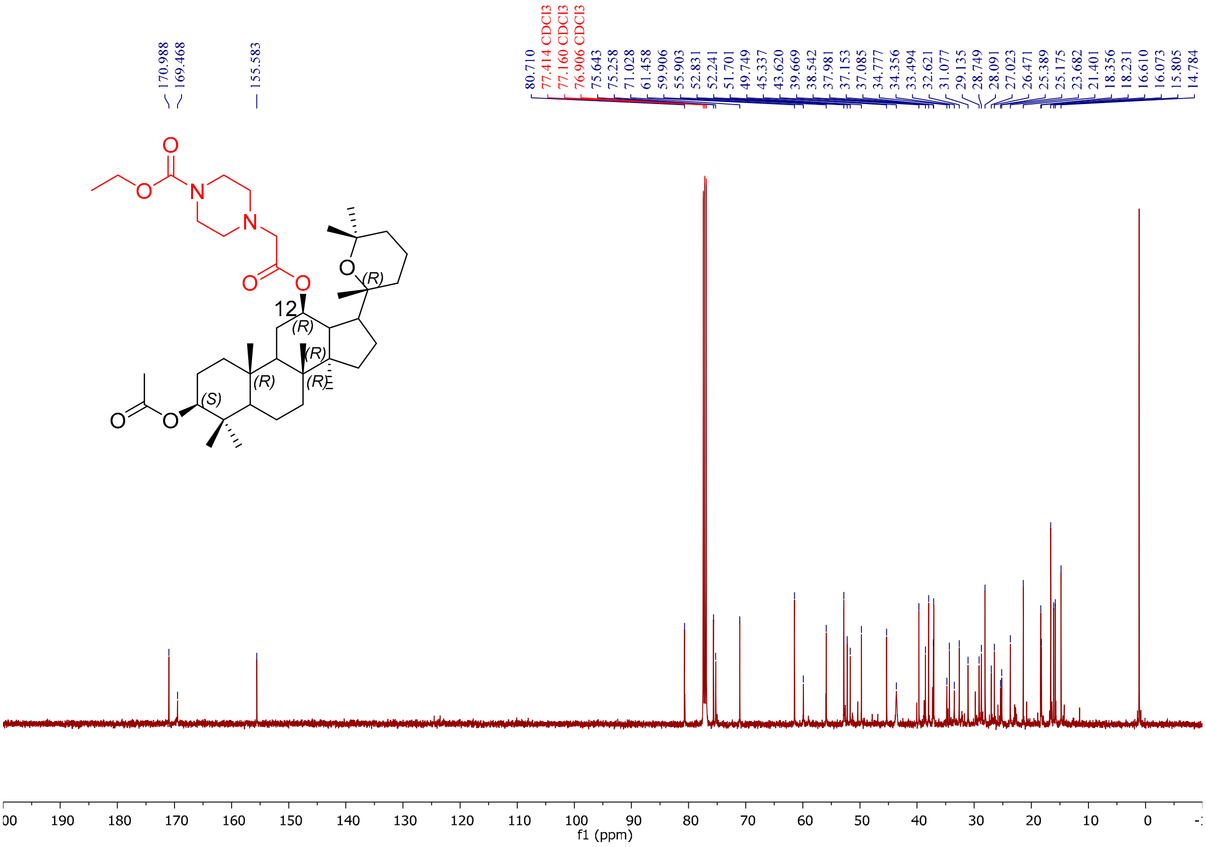

Figure S65. ^{1}H NMR Spectrum of compound **21** (500 MHz, $CDCl_3$)

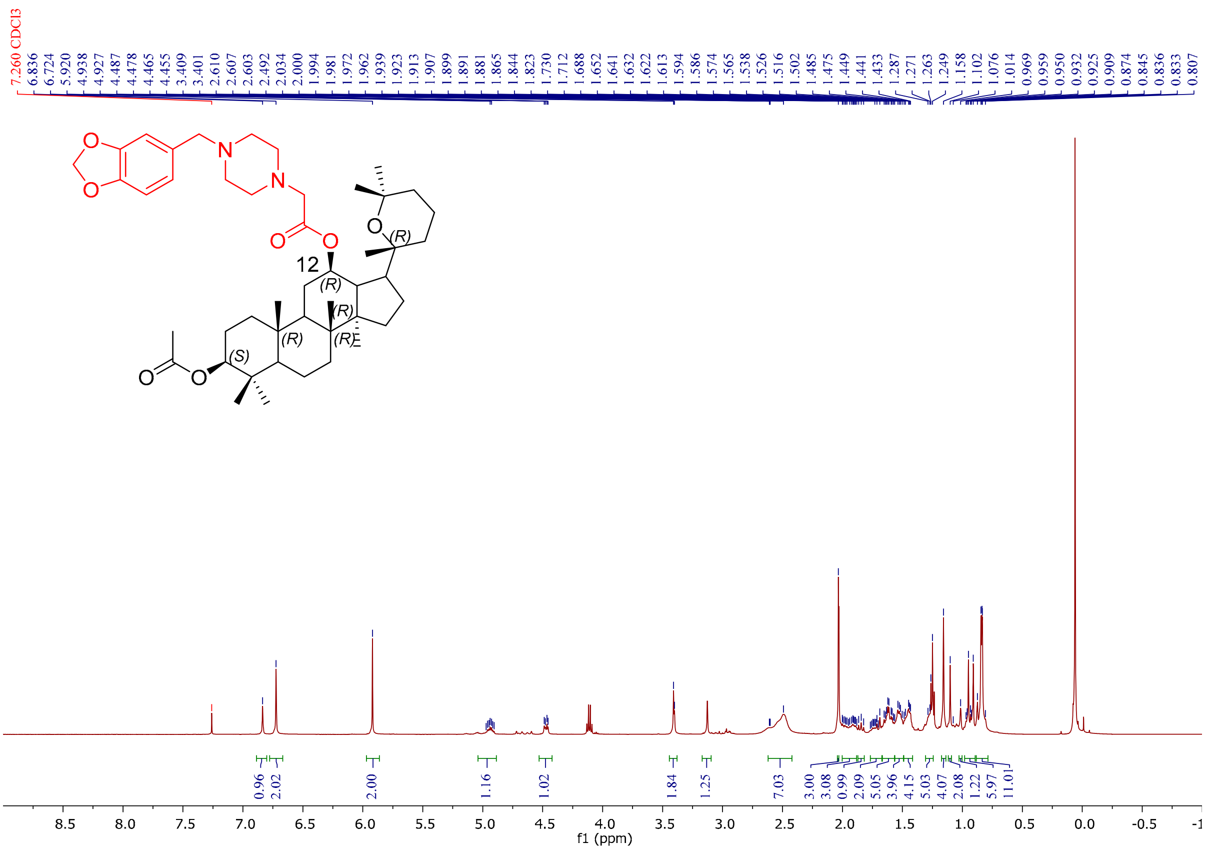

Figure S66. ^{13}C NMR Spectrum of compound **21** (126 MHz, $CDCl_3$)

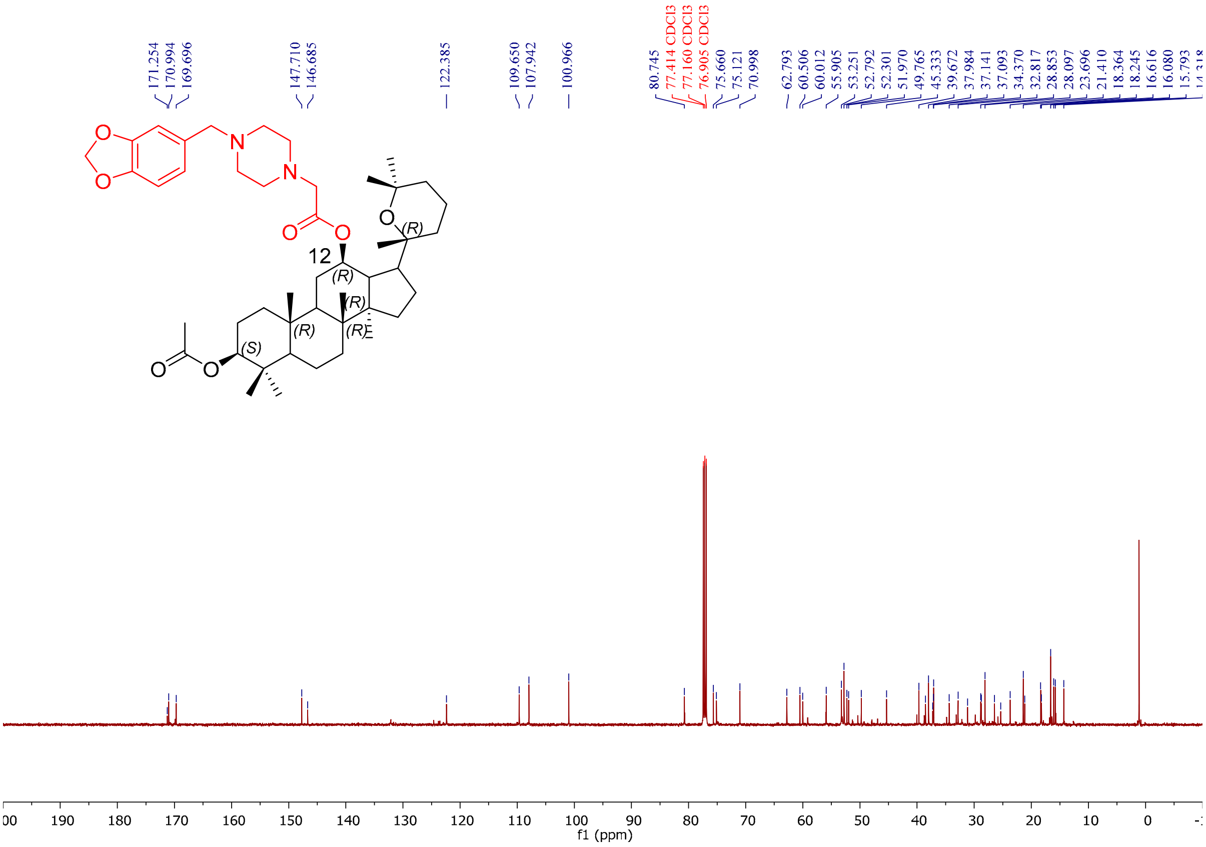

Figure S67. ^{1}H NMR Spectrum of compound **22** (500 MHz, $CDCl_3$)

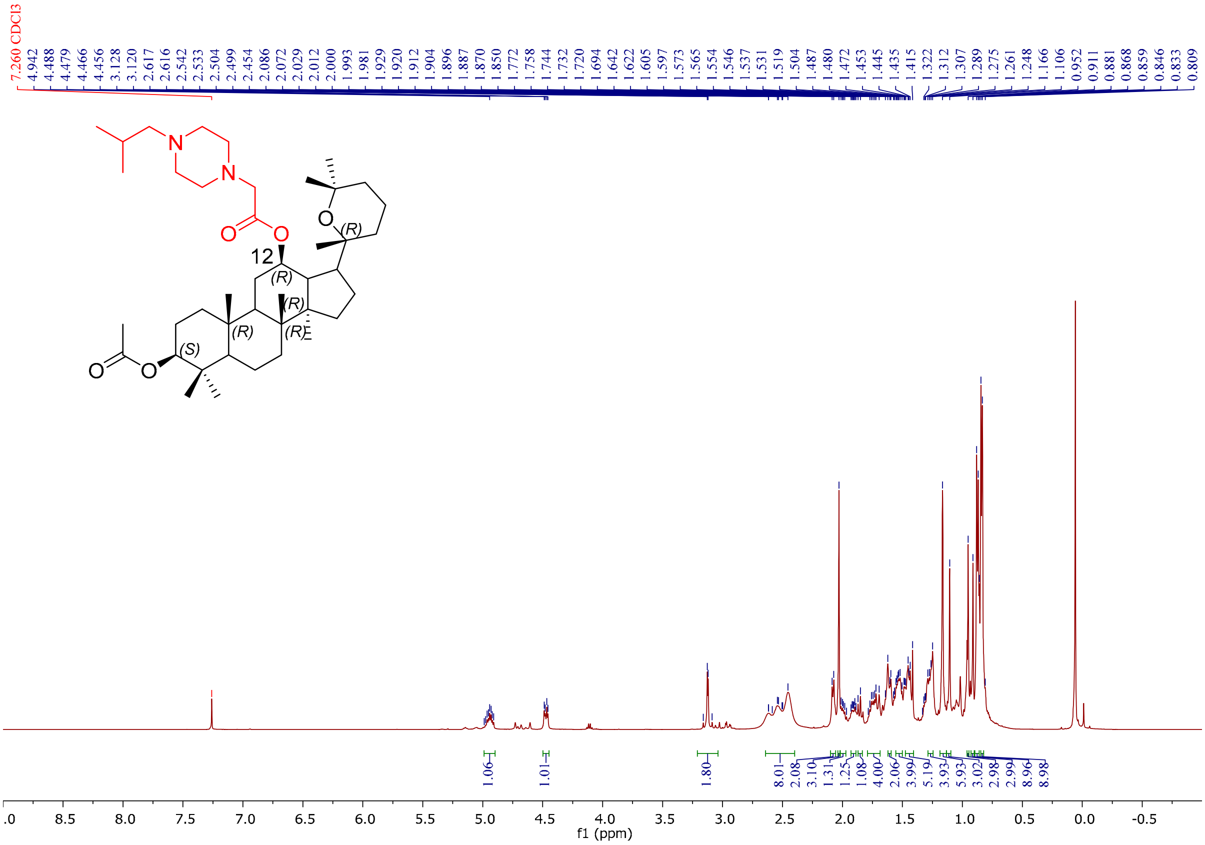

Figure S68. ^{13}C NMR Spectrum of compound **22** (126 MHz, $CDCl_3$)

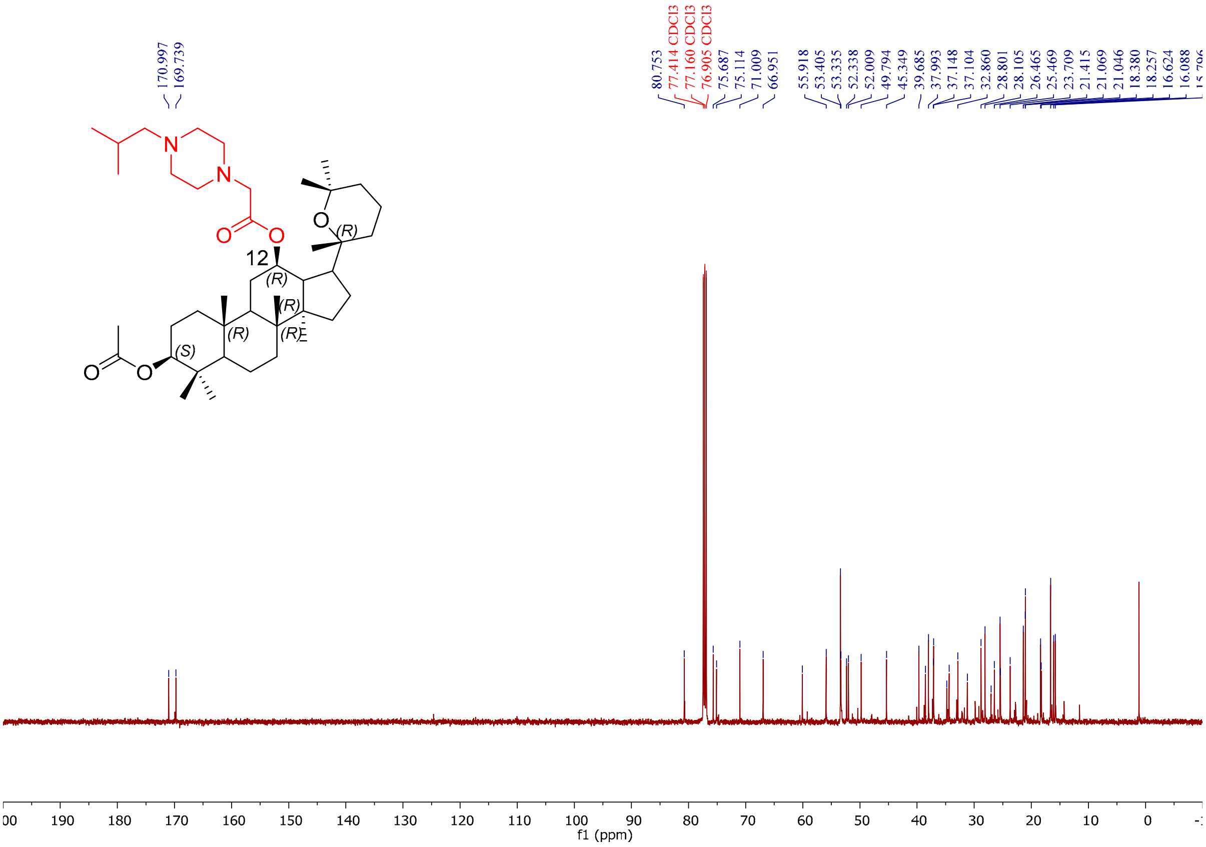

Figure S69. ^{1}H NMR Spectrum of compound **23** (500 MHz, $CDCl_3$)

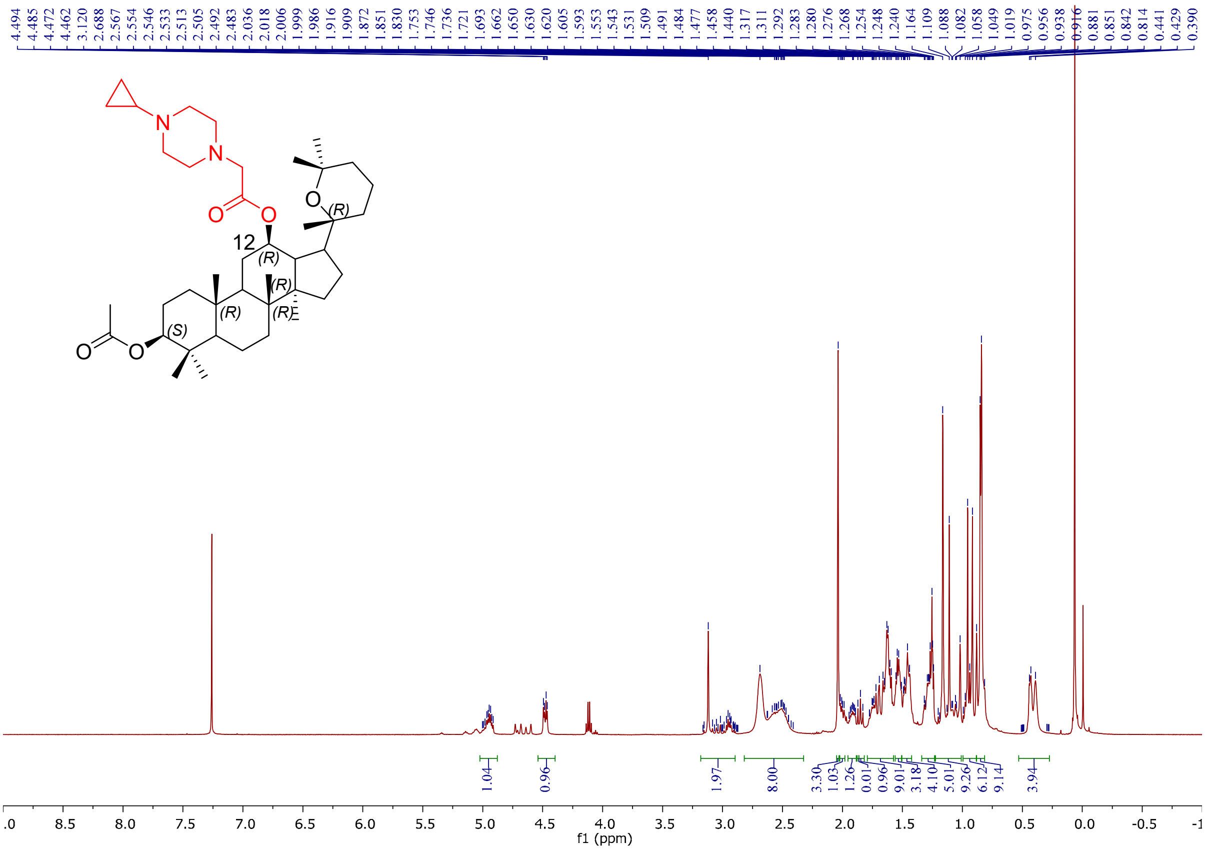

Figure S70. ^{13}C NMR Spectrum of compound **23** (126 MHz, $CDCl_3$)

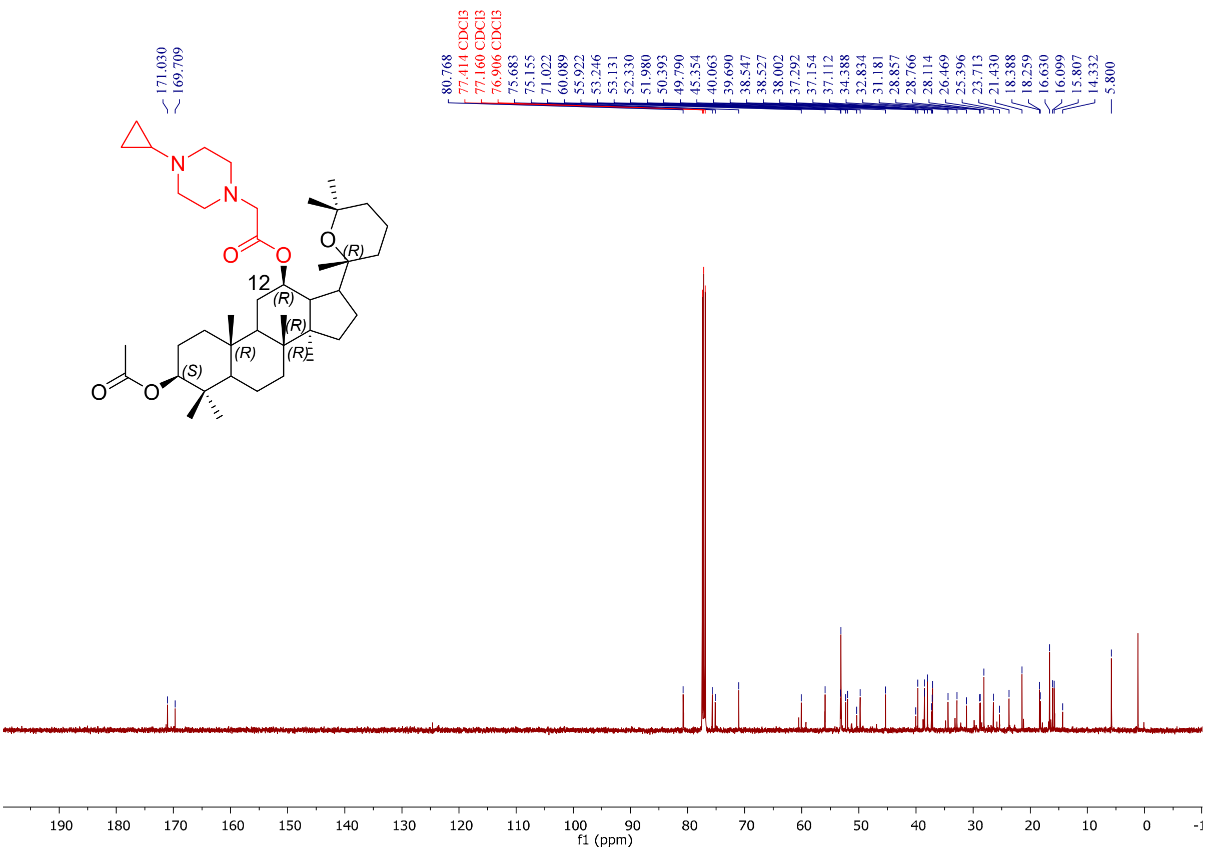

Supplement: Supplementary file 1 [file molecules-31-03225-s001.zip › molecules-4517150-supplementary.pdf]
